# Supplementary material for: Global certification of wild poliovirus eradication: insights from modelling hard-to-reach subpopulations and confidence about the absence of transmission
Source: BMJ Open. 2019 Jan 15;9(1):e023938. doi: 10.1136/bmjopen-2018-023938 (PMC6340450; doi:10.1136/bmjopen-2018-023938)

**APPENDIX for "Certification of global eradication: The role of hard-to-reach subpopulations and confidence about the absence of transmission"**

Radboud J. Duintjer Tebbens,[1] Dominika A. Kalkowsa,[1] Kimberly M. Thompson[1]

## ***Differential-equation based model and results***

The DEB model we use to examine the role of subpopulations [38] made simplifying assumptions about what a high-risk population might look like and otherwise adopted the comprehensive structure and setting-invariant model inputs of a previously developed and calibrated differential-equation based poliovirus transmission and OPV evolution model.[2 30] The following text from the appendix of a prior publication [45] (with references renumbered) briefly describes the model and Figures A1-2 and Table A1 cited in the text provide the model structure and generic inputs (i.e., model inputs that remain the same for all populations).

"The differential equation-based poliovirus transmission and OPV evolution model (DEB model) [2] tracks the movement of people between demographic age groups (grouped into mixing age groups that mix preferentially amongst themselves), and for each serotype between oropharyngeal and intestinal infection stages (resulting in potential oropharyngeal and fecal-oral transmission, respectively), immunity states, and waning stages. Figure A1 provides an overview of the model structure based on prior work.[2] Figure A1a depicts the immunity states with the flows that move individuals in and out of them and Figure A1b details how effectively vaccinated or infected individuals progress through different stages of infection and, in the event of infection with OPV, through OPV evolution stages. The model assumes that active immunity from prior vaccination or infection results in permanent protection from polio (disease), but only partial protection from subsequent infection and participation in transmission, depending on the nature of immunity (IPV-induced vs. LPV-induced or both) and time since the last exposure (i.e. waning stage). The model includes 5 waning stages, 6 fecal-oral and 6 oropharyngeal infection stages (2 latent and 4 infectious, with varying degrees of infectiousness), and also accounts for a delay between IPV receipt and development of the immune response that moves individuals to the next IPV immunity state. In Figure A1a, we note that the model assumes identical properties for "IPV and LPV" and "≥ 2 LPV infections" and that the recent waning stages of these immunity states represent the highest degree of immunity to transmission in the model. The model further tracks OPV evolution by moving individuals infected with the OPV parent strain (stage 0) through 20 successive reversion stages that can each transmit and that come with increasing paralysis-to-infection ratios and relative basic reproduction numbers ($R_0$ values) compared to homotypic WPVs. The last reversion stage (stage 19) represents fully-reverted VDPVs with assumed paralysis-to-infection ratio and $R_0$ equivalent to homotypic WPVs. For WPVs or any OPV reversion stage, the DEB model mimics die-out by setting the force-of-infection for the given strain to 0 whenever its effective prevalence of infections resides below a calibrated threshold of 5 per million people. Consequently, OPV-related viruses can only continue to transmit and thus evolve to cVDPVs through successive infections when low enough population immunity to transmission permits circulation of the OPV viruses introduced in the population through vaccination. We fixed the die-out process, model structure, and numerical

model inputs that characterize them across all populations we modeled and Table A1 includes the corresponding generic model inputs. […]

"Figure A2 summarizes the results of the model calibration process, based on prior work.[2] With the generic model inputs from Table A1 fixed, we compared our model behavior against i) data on children with non-polio acute flaccid paralysis who reported no receipt of OPV for northern India (modeled separately for Western Uttar Pradesh (WUP) and Bihar) and northwest (NW) Nigeria; ii) data on polio incidence and die-out of endemic WPV transmission for all situations and serotypes (shown in Figure A2 for WPV1 and WPV3 in northern India and northwest Nigeria and for all 3 WPV serotypes in the USA); iii) data from WPV importation outbreak behavior in the Netherlands, Tajikistan, and Albania; iv) data on age distributions of cases for all situations in which meaningful data was available (shown in Figure A2 for the Netherlands, Tajikistan, and Albania); v) available serogical data on the effect of secondary OPV immunity in the USA and Cuba (not shown); vi) indigenous emergence of cVDPVs (shown in Figure A2 for northern India, NW Nigeria (both serotype 2), Haiti, and Madura in Indonesia (both serotype 1); and vii) no indigenous emergence of cVDPVs in all other situations and serotypes (die-out of serotype 1 OPV-related viruses shows in Figure A2 for Cuba and Haiti). We subsequently applied the model to successfully reproduce the asymptomatic transmission of an imported WPV1 in Israel in 2013.[31]" [45, online supplement pp. 1-2]

Most critically in the context of certification questions, the DEB model approximates interruption of live poliovirus transmission (i.e., of an OPV, WPV, vaccine-derived poliovirus (VDPV), or OPV-related strain) in a population to occur when the effective infectiousness-weighted proportion of the population infectious with that poliovirus drops below 5 per million people (i.e., the transmission threshold $EPI^{*}$).[2] While this simplifies the true die-out behavior, which depends on local heterogeneity and chance, it appears capable of generating WPV die-out times consistent with observations in a broad range of settings.[2 30 31 41] Moreover, when applied to the persistence of OPV-related viruses that evolve to fully transmissible and neurovirulent circulating VDPVs (cVDPVs), the approximation produces cVDPV outbreaks for conditions in which they occurred (e.g., in Hispaniola[46] and Nigeria[47]) and no cVDPV outbreaks for conditions in which they did not occur despite OPV use and cessation (e.g., in Cuba[48] and the USA[49]).[2]

Use of the hypothetical model clarified that under-vaccinated subpopulations can sustain poliovirus transmission independently despite high coverage in the surrounding general population and showed how the minimum coverage needed to interrupt transmission depends on the degree of isolation and the relative size of the under-vaccinated subpopulation.[38] To explore the role of hard-to-reach under-vaccinated subpopulations for certification questions, we modified the hypothetical model in two ways and added a stochastic layer on top of the DEB model to simulate polio case detections. The first modification consisted of desynchronizing the time when vaccination starts in the general and under-vaccinated subpopulations to simulate the concept of a population that remains inaccessible for an extended period of time. Specifically, we run the model, which assumes equal birth and death rates and thus no population growth (Table 1), without vaccination for 30 years to settle into the endemic equilibrium, and then instantly change the routine immunization coverage in the general population with three OPV doses to 0.95, which lies well above the threshold of 0.92 needed to interrupt transmission in a closed population with similar characteristics.[38] However, we assume that the under-vaccinated subpopulation initially remains completely unreached by vaccination, with vaccine introduction

in the under-vaccinated subpopulation occurring 10 years after vaccine introduction in the general population. Desynchronizing the introduction of vaccination affects the dynamics and effectively makes it more difficult to interrupt transmission after introducing vaccination in the last subpopulation. To offset this effect, we consider a different hypothetical population with a slightly higher average per-dose take rate for OPV of 0.6 instead of 0.5 in the original analysis[38] (e.g., due to lower exposure to enteric viruses that interfere with vaccine take [50]). As in the original analysis,[38] we vary the coverage in the under-vaccinated subpopulation, the relative size of the under-vaccinated subpopulation compared to the total population, and the degree of preferential mixing, characterized by the proportion of potentially infectious contacts of individuals in the under-vaccinated subpopulations with other individuals in the same subpopulation ($p_{within}$).

Figure A3 shows the behavior of the incidence of infections in fully susceptible individuals and infants born with maternal immunity as a function of the varied DEB model inputs. Generally, the model yields incidence proportional to population size before vaccination starts. After the introduction of vaccination with high coverage in the general population, the initially still unvaccinated subpopulation becomes the main contributor to the total incidence. However, with less than 100% coverage in the general population and some interaction between the two populations (i.e., $p_{within}<1$), some incidence continues to occur in the general population as exported viruses find unvaccinated individuals. Lower values of $p_{within}$ imply more interaction between the two populations and result in more incidence in the general population before vaccination in the under-vaccinated subpopulation begins (middle column of Figure A3). The relative size of the under-vaccinated subpopulation also affects the extent to which the under-vaccinated subpopulation affects the general population (right column of Figure A3). With base case model inputs, the minimum coverage in the under-vaccinated subpopulation to interrupt transmission equals 0.82. Higher coverage values mean interruption occurs sooner after the introduction of vaccination in the under-vaccinated subpopulation, while lower coverage values mean that transmission continues and can eventually rebound and settle into a new equilibrium (left column in Figure A1).

While the prior approach used fully stochastic transmission models to randomly generate infections, die-out, and polio cases and detections,[19-22] for efficiency we use post-hoc processing of DEB model results to randomly generate only the times when polio cases and detections stochastically occur. Specifically, for each setting of the DEB model, we record the deterministic realization of the daily incidence of infections in fully susceptible individuals of any age and 50% of infants less than 3 months of age born with maternal immunity, which represent the only individuals at risk of becoming a polio case in the DEB model.[2] We then randomly determine the number of polio cases resulting from the infection incidence on each day using a Poisson draw with a rate equal to the infection incidence multiplied by the PIR. For each generated case, we use a separate uniform random draw to determine whether it results in a detection based on each of the detection probabilities in Table 1 (e.g., a random uniform draw of 0.45 would mean that the case results in a detection only for detection probabilities of more than 0.45). For each DEB model setting, we repeat the post-hoc stochastic process 10,000 times and we start generating cases 10 years before vaccination starts in the general population, which we assume starts vaccination 10 years earlier than the under-vaccinated subpopulation (see appendix). The precise choice of when to start randomly generating cases exerts negligible

influence on the results as long as it occurs before cases become rare (i.e., before the interval between cases becomes longer). For simplicity, although prior work showed the significant role of serotype differences and seasonality,[20, 22] the hypothetical model inputs reflect WPV1 and assumes no seasonality. A limitation arises from the direct scaling of the DEB model with absolute population size, such that die-out depends on the effective proportion of infectious individuals rather than the absolute number. Using the post-hoc stochastic analysis, the absolute population size affects the number of infections, which affects the typical interval between detected cases. We show that CNC95% increases substantially for smaller absolute population sizes.

Our initial findings motivated analysis of the minimum population size that can sustain WPV circulation on its own to determine whether the upper bound on the CNC95% of 9 years could occur in real populations. However, for population sizes far below 100,000, the DEB model becomes inadequate because it allows prevalence to remain above the die-out threshold even with only fractional numbers of infections (i.e., less than one infected person). Therefore, we used a fully stochastic model to explore questions of minimum population size. We run the model 10,000 times for different population sizes and initial conditions and report the distribution of the duration of circulation and the CNC95%.

**Exploration of the causal interactions relevant to global WPV certification decisions with an influence diagram (Figure 5)**

Table A2 provides indicative estimates of the key quantities in Figure 5, based on the literature. Figure 5 assumes that policy makers explicitly or implicitly set a *desired confidence about no WPV circulation at OPV cessation*. In reality, they may focus on the confidence at certification, but given that it takes some fixed *preparation time needed between certification and OPV cessation*, any set confidence at the time of certification corresponds to some *desired confidence about no WPV circulation at OPV cessation.* A higher desired confidence level implies a longer *time between last detection and certification.* This time decreases with increasing investments in immunization and surveillance from the *GPEI budget* through *population immunity maintenance* and the *perceived pre-certification surveillance quality*, respectively. The main drawback of a longer *time between last detection and OPV cessation* comes in the form of longer OPV use in most countries, which results in *planned immunization costs* and *OPV-related pre-cessation polio cases* (i.e., vaccine-associated paralytic polio and VDPVs). In addition, with some globally-recommended or nationally-preferred *duration of IPV after OPV cessation,* later OPV cessation would imply greater overall IPV costs, because global IPV use already started (i.e., only the end, and not the beginning of IPV use depends on the timing of cessation of the last OPV serotypes). These drawbacks together lead to *financial and societal costs of planned immunization*. This includes the monetary equivalent of the *OPV-related polio cases*, which depends on the country income-level-dependent *societal costs per polio case.*

On the left side of Figure 5, we see the benefits of setting a higher *desired confidence about no WPV circulation at OPV cessation.* A higher confidence implies a lower *probability of a WPV reemergence after OPV cessation* (all else being equal). However, this probability does not directly equal the reciprocal of the confidence in the event of a *gap between perceived and actual surveillance quality.* Specifically, if the *perceived pre-certification surveillance quality* exceeds

the *actual pre-certification surveillance quality*, then the true *probability of WPV reemergence after OPV cessation* equals more than 1 minus the *desired confidence about no WPV circulation at OPV cessation*, and vice versa. This potential discrepancy highlights the importance of continued assessment of surveillance quality and assurance of high surveillance quality. A lower *GPEI budget* also decreases *population immunity maintenance* and thus increases the *probability of WPV reemergence after OPV cessation*, which implies an increase in *expected WPV reemergence outbreaks.* Unlike other possible types of post-cessation outbreaks, a WPV reemergence would almost certainly occur in the most challenging populations. Any such reemergences would lead to *expected polio cases due to WPV reemergence outbreaks* and *expected costs to respond to WPV emergence outbreaks*. The expected costs and cases decrease with higher *post-cessation surveillance quality*, which affects the extent of viral spread at the time of outbreak detection (and beyond), and with a better *outbreak response quality and timeliness,* which both increase the probability of effective outbreak control.[51] However, the occurrence of any outbreaks comes with some probability of uncontrolled outbreaks, either by failing to control the original outbreak virus, or by creating new cVDPV outbreaks with the OPV vaccine used in the response. This implies some *probability of OPV restart due to WPV reemergences*, which would carry very significant *expected costs due to an OPV restart triggered by WPV reemergence* and *expected cases due to an OPV restart triggered by WPV emergence* (Table A2). For moderate or high *probability of OPV restart due to WPV reemergences*, the resulting *expected costs due to OPV restarts triggered by WPV reemergence* and *expected cases due to OPV restarts triggered by WPV reemergence* would likely dwarf the costs and cases associated with any controlled outbreaks due to WPV reemergences and would therefore drive the *expected financial and societal costs of possible WPV reemergences.*

Together with the *surveillance costs*, which act to moderate the costs of delayed OPV cessation or premature OPV cessation, the *expected financial and societal costs of possible WPV reemergences* and the *financial and societal costs of planned immunization* together make up the *total financial and societal costs (associated with any given desired confidence about no WPV circulation at OPV cessation).* The costs of possible WPV emergences and the costs of planned immunization move in opposite directions as a function of the *desired confidence about no circulation at OPV cessation.*

Figure 5 also highlights the consequences of the GPEI already scaling down some of its supplemental immunization and surveillance activities. While scaling down saves costs in the short term, doing so could lead to larger long-term costs by delaying certification and OPV cessation (i.e., requiring higher confidence about no circulation), which would imply that OPV cessation could occur in the context of lower global population immunity to transmission and lower ability to rapidly detect outbreaks. This ultimately implies an increase in the expected *total financial and societal costs (associated with any given desired confidence about no WPV circulation at OPV cessation)*. For visual simplicity, Figure 5 omitted some additional complexity involved in this decision. Furthermore, given that the confidence about no circulation increases with time after the last detection, we could have equivalently centered Figure 5 around finding the optimal time between the last detection and certification or OPV cessation. The amounts in Table A2 highlight the significant financial and humanitarian stakes involved in finding the optimal *desired confidence about no WPV circulation at OPV cessation.*

**Table A1: Generic inputs of the DEB model[2 30] (adopted from the online supplement of Duinter Tebbens et al., 2017[45])**

| Model input (symbol) | Best estimate | Source |
|---|---|---|
| Relative susceptibility ($\sigma$) of recent immunity states (for PV1;PV2;PV3) | | [52 53] |
| - Maternally immune | 0.78;0.79;0.77 | |
| - 1 successful IPV | 0.91;0.92;0.90 | |
| - 2 successful IPV | 0.80;0.80;0.79 | |
| - ≥ 3 successful IPV | 0.72;0.72;0.71 | |
| - 1 LPV infection | 0.42;0.43;0.41 | |
| - ≥ 2 LPV infections | 0.21;0.22;0.20 | |
| - IPV and LPV | 0.21;0.22;0.20 | |
| Duration of latent period ($\xi^{fec}$ or $\xi^{oro}$, in days) | ~ 3[a] | [52 53] |
| Duration of fecal infectiousness ($\gamma^{fec}$, in days) of recent immunity states (for PV1;PV2;PV3) | | [52 53] |
| - Fully susceptible | 28.0;27.8;28.3 | |
| - Maternally immune | 24.6;24.6;24.6 | |
| - 1 successful IPV, | 24.5;24.4;24.7 | |
| - 2 successful IPV | 21.1;20.8;21.3 | |
| - ≥ 3 successful IPV | 18.0;17.7;18.2 | |
| - 1 LPV infection | 11.6;10.5;10.5 | |
| - ≥ 2 LPV infections | 10.1;8.9;8.9 | |
| - IPV and LPV | 10.1;8.9;8.9 | |
| Duration of oropharyngeal infectiousness ($\gamma^{oro}$, in days) of recent immunity states (no serotype differences) | | [52 53] |
| - Fully susceptible | 13.4 | |
| - Maternally immune | 11.9 | |
| - 1 successful IPV | 9.9 | |
| - 2 successful IPV | 6.6 | |
| - ≥ 3 successful IPV | 6.1 | |
| - 1 LPV infection | 5.0 | |
| - ≥ 2 LPV infections | 3.7 | |
| - IPV and LPV | 3.7 | |
| Relative fecal infectiousness ($\pi^{fec}$) of recent immunity states (for PV1;PV2;PV3) | | [52 53] |
| - Maternally immune | 0.96;0.96;0.95 | |
| - 1 successful IPV | 0.92;0.92;0.91 | |
| - 2 successful IPV | 0.70;0.69;0.68 | |
| - ≥ 3 successful IPV | 0.61;0.59;0.59 | |
| - 1 LPV infection | 0.39;0.43;0.43 | |
| - ≥ 2 LPV infections | 0.20;0.23;0.23 | |
| - IPV and LPV | 0.20;0.23;0.23 | |
| Relative oropharyngeal infectiousness ($\pi^{oro}$) of recent immunity states (no serotype differences) | | [52 53] |
| - Maternally immune | 0.68 | |
| - 1 successful IPV | 0.30 | |
| - 2 successful IPV | 0.17 | |
| - ≥ 3 successful IPV | 0.12 | |
| - 1 LPV infection | 0.33 | |
| - ≥ 2 LPV infections | 0.21 | |
| - IPV and LPV | 0.21 | |
| Number of infection stages | | |
| - Latent period ($r$) | 2 | |
| - Infectious period ($s$) | 4 | |
| Relative weight of infection stages, compared to average weight over the infectious period ($\theta_j$, $j$=0,…,$r$+$s$-$1$) | | [52 53] |

| | | |
|---|---|---|
| - Infection stage 0 and 1 (latent stages)<br>- Infectious stage 2<br>- Infectious stage 3<br>- Infectious stage 4<br>- Infectious stage 5 | 0<br>12/17<br>40/17<br>12/17<br>4/17 | |
| IPV immunity delay ($\varphi$, in days) | 7 | 54 |
| Number of waning stages ($nw$) | 5 | |
| Shape of waning function ($z_w$) | 5 | 52 53 |
| Average time to reach last waning stage ($\rho$, in days)<br>- Type 1&2<br>- Type 3 | <br>4×365<br>3×365 | 52 53 |
| Average time for maternal immunes to wane to fully susceptible ($\rho_{MI}$, in days) | 0.25×365 | 52 53 |
| Relative susceptibility ($\sigma$) for last waning stage (no serotype differences)<br>- 1 successful IPV<br>- 2 successful IPV<br>- ≥ 3 successful IPV<br>- 1 LPV infection<br>- ≥ 2 LPV infections<br>- IPV and LPV | <br>1.0<br>1.0<br>1.0<br>0.8<br>0.7<br>0.7 | 52 53 |
| Duration of fecal infectiousness ($\gamma^{fec}$, in days) of last waning stage (for PV1;PV2;PV3)<br>- 1 successful IPV<br>- 2 successful IPV<br>- ≥ 3 successful IPV<br>- 1 LPV infection<br>- ≥ 2 LPV infections<br>- IPV and LPV | <br>26.6;26.4;26.9<br>25.2;25.0;25.5<br>23.8;23.6;24.1<br>14.0;13.9;14.1<br>11.4;11.4;11.6<br>11.4;11.4;11.6 | 52 53 |
| Duration of oropharyngeal infectiousness ($\gamma^{oro}$, in days) of last waning stage (no serotype differences)<br>- 1 successful IPV<br>- 2 successful IPV<br>- ≥ 3 successful IPV<br>- 1 LPV infection<br>- ≥ 2 LPV infections<br>- IPV and LPV | <br>11.4<br>6.7<br>6.6<br>6.7<br>4.0<br>4.0 | 52 53 |
| Relative fecal infectiousness ($\pi^{fec}$) of last waning stage (no serotype differences)<br>- 1 successful IPV<br>- 2 successful IPV<br>- ≥ 3 successful IPV<br>- 1 LPV infection<br>- ≥ 2 LPV infections<br>- IPV and LPV | <br>0.95<br>0.9<br>0.85<br>0.5<br>0.3<br>0.3 | 52 53 |
| Relative oropharyngeal infectiousness ($\pi^{oro}$) of last waning stage (no serotype differences)<br>- 1 successful IPV<br>- 2 successful IPV<br>- ≥ 3 successful IPV<br>- 1 LPV infection<br>- ≥ 2 LPV infections<br>- IPV and LPV | <br>0.43<br>0.25<br>0.13<br>0.5<br>0.3<br>0.3 | 52 53 |
| Number of reversion stages ($h$) | 20 | |
| Shape of reversion function with respect to:<br>- $R_0$ ($z_r$)<br>- ln(PIR) ($z_p$) | <br>1<br>2.5 | |

| | | |
|---|---|---|
| Average time to reach last reversion stage ($\varepsilon$, in days) (for PV1;PV2;PV3) | 620.5; 408; 620.5 | [30] |
| Paralysis-to-infection ratio for fully susceptible individuals infected with OPV ($PIR_0$) (for PV1; PV2;PV3) | $0.26\times10^{-6}$; $1.2\times10^{-6}$; $1.8\times10^{-6}$ | |
| Paralysis-to-infection ratio for fully susceptible individuals infected with FRPV ($PIR_{h\text{-}1}$) (for PV1; PV2;PV3) | 0.005; 0.0005; 0.001 | [2 14 54] |
| Relative $R_0$ of OPV vs. FRPV ($\tau_0$) (for PV1; PV2; PV3) | 0.37;0.55;0.25 | [2 52 53] |
| Effective infectious proportion below which we assume 0 force-of-infection (transmission threshold $EPI^*$) | 5/1,000,000 | |
| Relative PIR for maternally immunes compared to fully susceptible individuals ($RPIR_{MI}$) | 0.5 | |
| Ratio of $R_0$ by serotype in the same setting (PV1:PV2:PV3) | 1:0.9:0.75 | [30] |
| Average incubation period ($\delta$, in days) | 10 | [54 55] |
| Demographics for all situations | Time series 1950-2100 | [56] |

**Acronyms:** CDC = (U.S.) Centers for Disease Control and prevention; cVDPV = circulating vaccine-derived poliovirus; DEB = differential equation-based FRPV = fully-reverted poliovirus; GPLN = Global Polio Laboratory Network; IPV = inactivated poliovirus vaccine; LPV = live poliovirus; OPV = oral poliovirus vaccine; PIR = paralysis-to-infection ratio; PV(1,2,3) = poliovirus (type 1, 2, or 3, respectively); $R_0$ = basic reproductive number; UN = United Nations; USA = United States of America; VAPP = vaccine-associated paralytic poliomyelitis; VP1 = viral protein 1; WPV(1,2,3) = wild poliovirus (type 1, 2, or 3, respectively)

**Notes:** [a] Mean estimates obtained from experts and used in the model for the different immunity states, serotypes, and excretion modes vary between 2.85 and 3.37 days

**Table A2: Indicative estimates of key variables from Figure 5**

| Variable | Estimate | Notes and sources |
|---|---|---|
| Preparation time needed between certification and OPV cessation | Approximately 1 year | Depends on when setting of the OPV cessation date occurs relative to certification [57] |
| Planned immunization costs | $1 billion in external GPEI funds per year, plus internal contributions | Most of the $1.1 billion GPEI budget for 2016 was for immunization and coordination of activities;[58] Countries may internally contribute at a similar rate as the external contributions;[59] The current GPEI budget projects a decrease from 2018 forward, which would imply some offset of costs for maintenance of activities, or alternatively the activities previously supported by external contributions may end, which would imply declines in programmatic activities and quality |
| OPV-related polio cases | Hundreds per year | Vaccine-associated paralytic polio cases,[60] which depends on timing of IPV doses,[61] and presumably local cVDPV outbreaks[62] |
| Surveillance costs | Around $100 million per year | The 2016 GPEI budget included $67 million in external support for surveillance and laboratories,[58] with additional significant internal contributions by countries [59 63] |
| Probability of OPV restart due to WPV reemergence | Unknown | Prior studies estimated an approximately 5% chance of an OPV restart due primarily to OPV-associated risks, although the actual implementation of risk management policies was not as good as suggested by these models.[59 64] |
| Immunization costs associated with an OPV restart | $ billions (hundreds of millions per year) | An OPV restart would involve reintroduction of OPV vaccination in most countries in perpetuity, with supplemental immunization activities needed in countries with insufficient routine immunization coverage.[59] Significant uncertainty exists about what an OPV restart would look like in practice. |
| Expected cases due to an OPV restart | Up to thousands per year | Reintroduction of OPV in most countries would result in hundreds of vaccine-associated paralytic polio cases per year and could result in continued cVDPV outbreaks in countries with insufficient routine immunization coverage that do no conduct regular preventive supplemental immunization activities.[59 64] |

**Figure A1: Schematic of the DEB model structure, adopted from Duintjer Tebbens et al. (2013)[2, p. 706]**

**(a) Immunity states and flows between them due to epidemiological events**

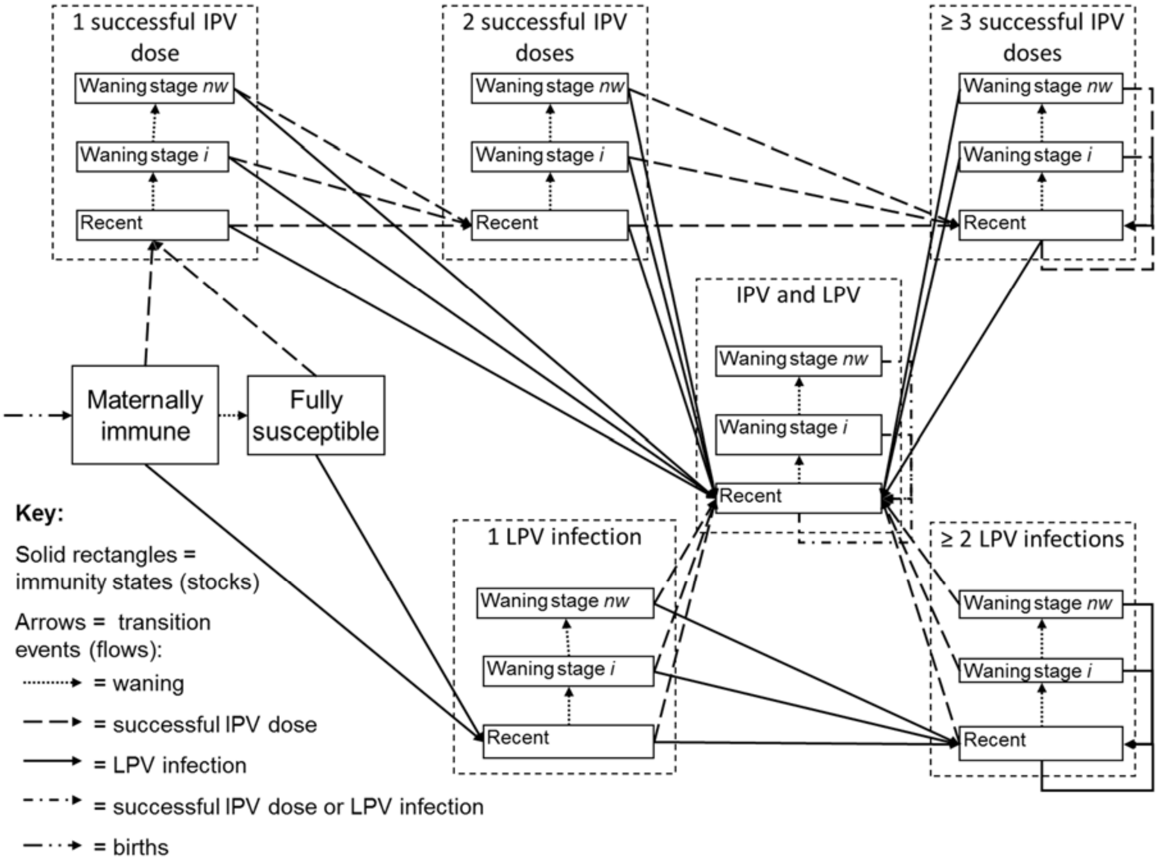

**(b) Progression through infection and reversion stages**

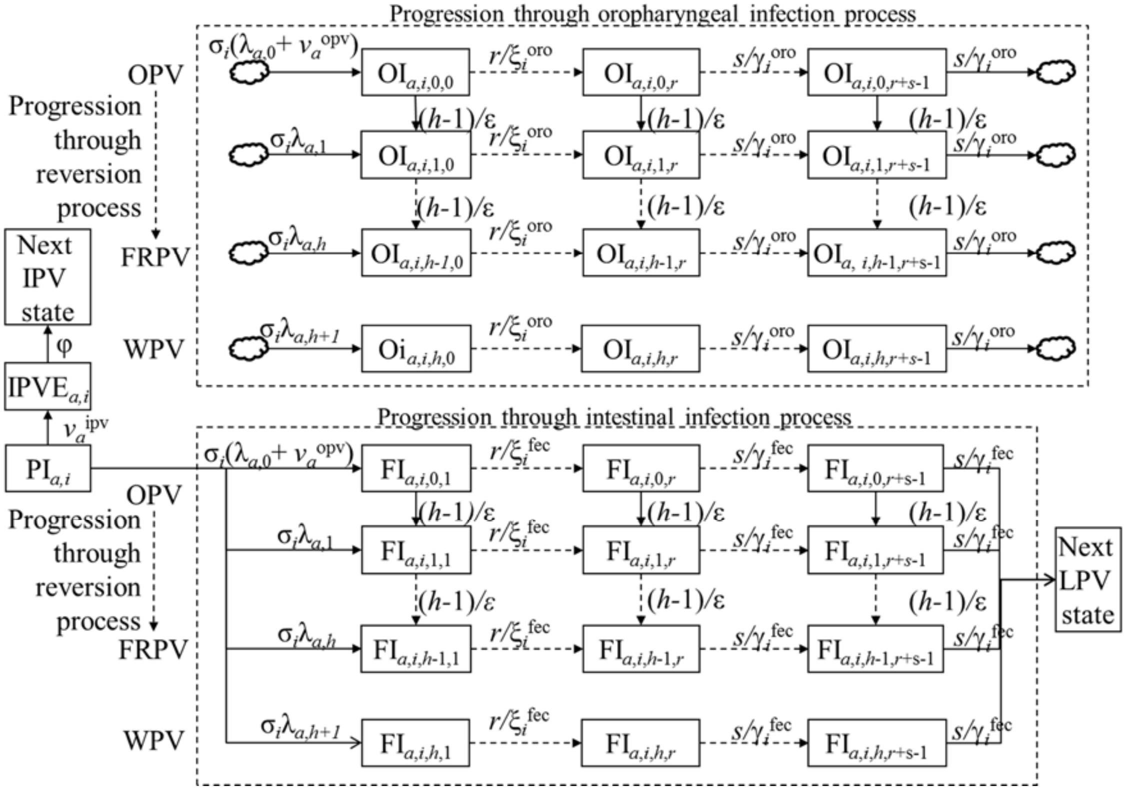


**"Acronyms:** FRPV = fully-reverted poliovirus; IPV = inactivated poliovirus vaccine; OPV = oral poliovirus vaccine; WPV = wild poliovirus**; Symbols:** $PI_{a,i}$ = partially infectible in age group *a* and immunity state *I*; $IPVE_{a,i}$ = IPV-exposed individual from immunity state *i* and age group *a*; $FI_{a,i,j,k}$ ($OI_{a,i,j,k}$) = individual in age group a from immunity state *i*, infected with virus strain *j* and in fecal (oropharyngeal) infection stage *k*; $\lambda_{a,j}$ = force-of–infection to age group *a* for virus strain *j;* $\nu_a^{ipv}$ ($\nu_a^{opv}$) = force-of-IPV(OPV)-vaccination to age group *a* as a result of routine and supplementary immunization; $\sigma_i$ = relative susceptibility for immunity state *i*; $\xi_i^{fec}$ ($\xi_i^{oro}$) = average duration of the fecal (oropharyngeal) latent period for immunity state *i*; $\gamma_i^{fec}$ ($\gamma_i^{oro}$) = average duration of the fecal (oropharyngeal) infectious period for immunity state *i*; *φ* = IPV immunity delay; *h* = number of reversion stages; *r* = number of latent stages; *s* = number of infectious stages" [2, p. 706]

**Figure A2: Summary results from the model calibration process, adapted from Duintjer Tebbens et al. (2013)[2]**

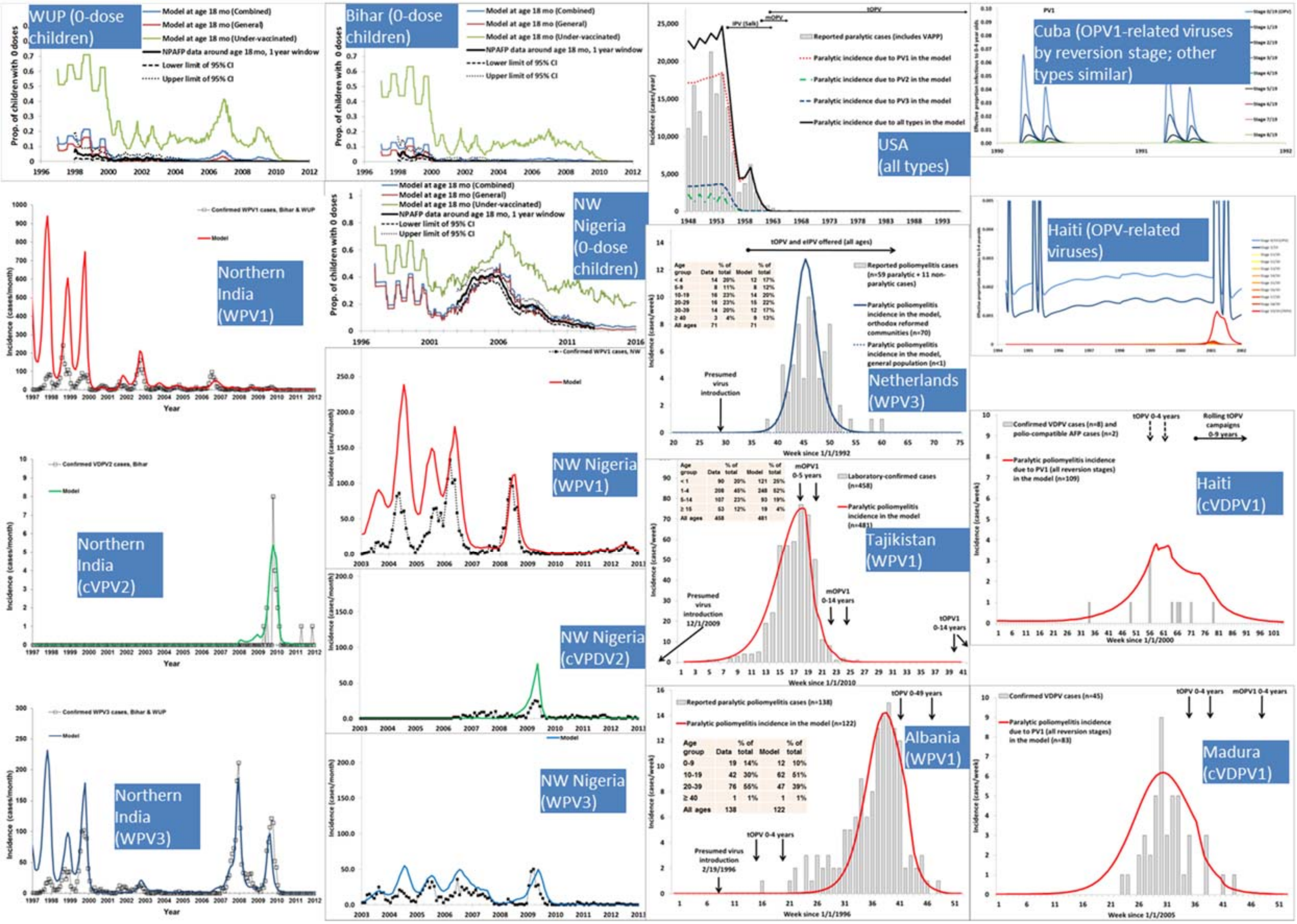

**Figure A3: Differential-equation based model results for base case model inputs and varied coverage (left column), varied degree of isolation with coverage 0.82 (middle column), and varied relative size with coverage of 0.82 (right column). The y-axis scales linearly with total population size (all figures assume a total population size of 1 million).**

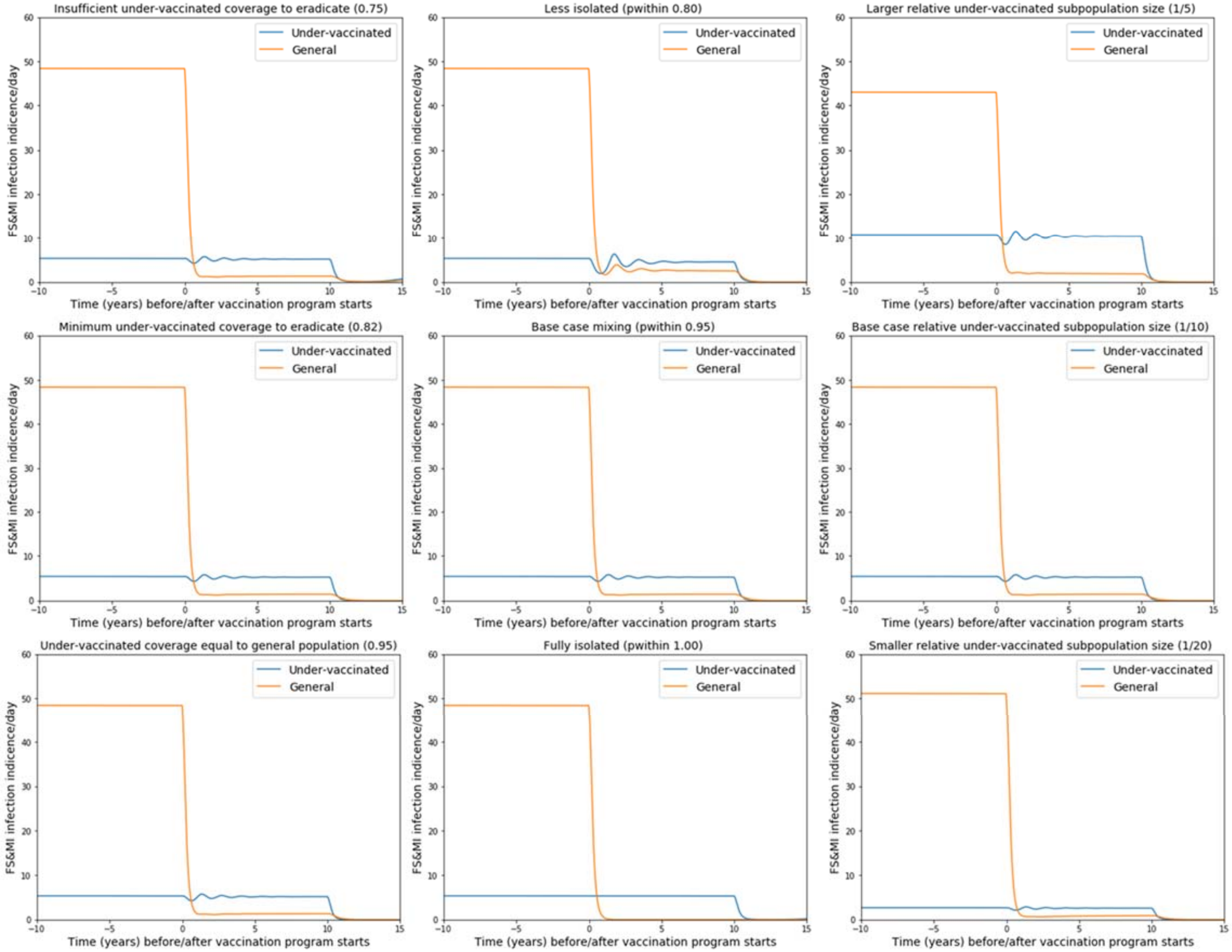

Supplement: Supplementary file 1 [file bmjopen-2018-023938supp001.pdf]
