# Supplementary material for: Global certification of wild poliovirus eradication: insights from modelling hard-to-reach subpopulations and confidence about the absence of transmission
Source: BMJ Open. 2019 Jan 15;9(1):e023938. doi: 10.1136/bmjopen-2018-023938 (PMC6340450; doi:10.1136/bmjopen-2018-023938)
Supplement: Author's manuscript [file bmjopen-2018-023938.draft_revisions.pdf]

BMJ Open

BMJ Open is committed to open peer review. As part of this commitment we make the peer review history of every article we publish publicly available.

When an article is published we post the peer reviewers' comments and the authors' responses online. We also post the versions of the paper that were used during peer review. These are the versions that the peer review comments apply to.

The versions of the paper that follow are the versions that were submitted during the peer review process. They are not the versions of record or the final published versions. They should not be cited or distributed as the published version of this manuscript.

BMJ Open is an open access journal and the full, final, typeset and author-corrected version of record of the manuscript is available on our site with no access controls, subscription charges or pay-per-view fees (http://bmjopen.bmj.com).

If you have any questions on BMJ Open's open peer review process please email info.bmjopen@bmj.com

BMJ Open

Certification of global poliovirus eradication: The role of hard-to-reach subpopulations and confidence about the absence of transmission

Journal:	*BMJ Open*
Manuscript ID	bmjopen-2018-023938
Article Type:	Research
Date Submitted by the Author:	02-May-2018
Complete List of Authors:	Duintjer Tebbens, Radboud; Kid Risk, Inc.<br>Kalkowska, Dominika; Kid Risk, Inc.<br>Thompson, KM; Kid Risk, Inc.,
Keywords:	polio, eradication, certification, modeling

SCHOLARONE™
Manuscripts

For peer review only

Certification of global poliovirus eradication: The role of hard-to-reach subpopulations and confidence about the absence of transmission

Radboud J. Duintjer Tebbens,[1] Dominika A. Kalkowsa,[1] Kimberly M. Thompson[1]

1. Kid Risk, Inc., Columbus, OH, USA

Running title: **Poliovirus certification confidence**

Correspondence to: Kimberly M. Thompson, Kid Risk, Inc., 605 N High St, #253, Columbus, OH 43215, USA, Email: kimt@kidrisk.org

Abstract:

Objective: To explore the extent to which under-vaccinated subpopulations may influence the confidence about no circulation of wild poliovirus (WPV) after the last detected case.

Design and participants: We used a hypothetical model to examine the extent to which the existence of an under-vaccinated subpopulation influences the confidence about no WPV circulation after the last detected case as a function of different characteristics of the subpopulation (e.g., size, extent of isolation). We also used the hypothetical population model to inform the bounds on the maximum possible time required to reach high confidence about no circulation in a completely-isolated and unvaccinated subpopulation starting either at the endemic equilibrium or with a single infection in an entirely susceptible population.

Results: It may take over three years to reach 95% confidence about no circulation for this hypothetical population despite high surveillance sensitivity and high vaccination coverage in the surrounding general population if: (1) ability to detect cases in the under-vaccinated subpopulation remains exceedingly small, (2) the under-vaccinated subpopulation remains small and highly isolated from the general population, and (3) the coverage in the under-vaccinated subpopulation remains very close to the minimum needed to eradicate. Fully-isolated hypothetical populations of 4,000 people or less cannot sustain endemic transmission for more than 5 years, with at least 20,000 people required for a 50% chance of at least 5 years of sustained transmission in a population without seasonality that starts at the endemic equilibrium.

For peer review only - http://bmjopen.bmj.com/site/about/guidelines.xhtml

Notably, however, the population size required for persistent transmission increases significantly for realistic populations that include some vaccination and seasonality and/or that do not begin at the endemic equilibrium.

Conclusions: Significant trade-offs remain inherent in global polio certification decisions, which underscore the need for making and valuing investments to maximize population immunity and surveillance quality in all remaining possible WPV reservoirs.

Strengths and limitations of this study:

- Demonstrates the somewhat limited but important role of under-vaccinated subpopulations in the time required to achieve high confidence about no WPV transmission after the last reported case.
- Highlights competing trends as time increases such that for smaller population sizes continued transmission becomes exceedingly unlikely, while for larger population sizes undetected circulation becomes less likely due to the higher frequency of cases and greater chances of detection.
- Results underscore the importance of continued investments to maximize population immunity and surveillance quality.
- Analyses remain limited by model assumptions, but in abstract provide insights relevant to likely last poliovirus reservoirs.

Keywords: polio, eradication, certification, modeling

For peer review only

For peer review only - http://bmjopen.bmj.com/site/about/guidelines.xhtml

Background

Achieving the 1988 World Health Assembly polio eradication goal of ending all cases of poliomyelitis [1] requires a successful transition from the interruption of the current low level of wild poliovirus (WPV) transmission through coordinated cessation of all use of live attenuated oral poliovirus vaccine (OPV) to effective long-term risk management. The Global Polio Laboratory Network supports the Global Polio Eradication Initiative (GPEI) by testing stool samples from acute flaccid paralysis (AFP) cases and sewage samples for polioviruses. As the GPEI approaches success, the transition to the polio endgame has begun. The endgame involves significant complexity, because all countries must achieve and maintain sufficient population immunity [2-4] to stop and prevent the transmission of three separate poliovirus serotypes (i.e., 1, 2, and 3) and globally coordinate cessation of each OPV serotype.[5-7] In September 2015, the Global Certification Commission declared successful eradication of serotype 2 WPV (WPV2),[8] which represented a prerequisite to the globally-coordinated cessation of all serotype 2-containing OPV use. Global cessation of serotype 2-containing OPV occurred in late April and early May 2016, during which time over 150 countries stopped using trivalent OPV (tOPV, which contains all three serotypes) and switched to bivalent OPV (bOPV, which contains only serotypes 1 and 3 OPV).[9]

The Global Polio Laboratory Network reported the lowest number of annual paralytic serotype 1 WPV (WPV1) cases in 2017,[10] and no serotype 3 WPV (WPV3) cases since November 2012.[11] Successful WPV eradication requires stopping all transmission, which manifests as an absence of detected WPVs despite actively looking. With increasing time of not seeing cases (while actively looking), confidence increases about WPV die-out. However, the absence of evidence is not evidence of absence. Extended silent transmission can occur, because most poliovirus infections do not lead to symptoms and surveillance gaps can exist. For example, a WPV3 resurfaced in Sudan/Chad in 2004 after no reported cases during 1997-2003 [12] and a WPV1 resurfaced in Borno, Nigeria in 2016 after nearly 3 years with no reported cases [13]. The average paralysis-to-infection ratio (PIR), defined as the fraction of infections in fully susceptible individuals that leads to paralytic poliomyelitis (polio) symptoms, equals approximately 1/200, 1/2000, and 1/1000, for serotype 1, 2, and 3 WPV, respectively.[14] The last reported naturally-

For peer review only - http://bmjopen.bmj.com/site/about/guidelines.xhtml

occurring WPV2 case occurred in India in 1999,[15] and since then, only two episodes of WPV2 infections occurred that traced back to laboratory strains.[16 17] Despite the possibility of silent circulation, the absence of any naturally-occurring WPV2 cases for over 15 years (and in many countries for many decades) led to very high confidence about the die-out of WPV2 transmission.

Multiple prior mathematical modeling studies explored the probability of undetected circulation of WPVs in the absence of reported cases or other poliovirus detections. Polio eradication efforts in the Americas, which reported the last indigenous WPV case of any serotype in Peru in 1991,[18] motivated the first analysis and discussion of certification requirements. A statistical analysis of Pan American Health Organization epidemiological data reported less than a 5% chance of undetected indigenous WPV circulation after 4 years since the last reported confirmed case.[19] A simple, stochastic model of poliovirus transmission and die-out characterized the probability of undetected poliovirus circulation in a hypothetical, homogeneously mixed population of 200,000 people in a relatively low-income country, and estimated that not observing a case for 3 years provided 95% confidence about local extinction of WPV infections.[20] This seminal paper provided the foundation for appropriate characterization of the probability of undetected circulation as a function of the time since the last detected case.[20] Related modeling also explored theoretical thresholds to stop transmission [21] and estimated a minimum population size for persistent transmission of 50,000-100,000 in developing countries and over 200,000 in developed countries required to achieve at least 95% probability of poliovirus persistence for 5 years or more in the absence of vaccination.[22] These studies supported the 2004-8 GPEI Strategic Plan requirement of at least 3 years of no polio cases detected by AFP surveillance for certification,[23] which remains the current minimum requirement.[24] A 2012 study [25] relaxed some of the assumptions of the prior theoretical model [20] and highlighted that the probability of undetected circulation varied for different poliovirus serotypes, places, and conditions, which suggested the need to focus on appropriate characterization of conditions in the last likely WPV reservoirs.[25] A 2015 study [26] also used the prior model [20] to show that in the context of an instantaneous introduction of vaccination, the time of the last case relative to vaccine introduction further informs the confidence about the absence of circulation.

For peer review only - http://bmjopen.bmj.com/site/about/guidelines.xhtml

Subsequent analyses focused on modeling the conditions in specific and more realistic populations. A 2015 study [27] used a previously-developed poliovirus dynamic transmission model [2] applied to: recently-endemic transmission in two states in northern India,[28] endemic transmission in northwest Nigeria,[29] a 2010 outbreak in Tajikistan,[30] and transmission following a 2013 WPV1 introduction into Israel detected by environmental surveillance.[31] The study characterized the confidence about no undetected poliovirus circulation by serotype as a function of time without reported polio cases or environmental detections considering realistic assumptions for surveillance, immunization, and other national inputs.[27] The results suggested that time periods of 0.5 to 3 years without detected polio cases provided 95% confidence about the interruption of transmission in the context of perfect AFP surveillance depending on situation-specific characteristics (e.g., the overall population immunity, endemic versus outbreak conditions, and virus serotype).[27] This model also suggested longer times required for less-than-perfect AFP surveillance and potentially shorter times using highly-sensitive environmental surveillance based on the experience in Israel.[27] A recent statistical analysis of the 2013 WPV1 outbreak in Israel demonstrated a rapid increase in confidence about no undetected local transmission following outbreak response immunization after repeated negative environmental surveillance samples in a city.[32] A non-dynamic, statistical model [33] estimated a shorter time (compared to [27]) of 14 months required to reach high confidence about no undetected circulation. For its most conservative assumptions about surveillance and force-of-infection, the study estimated a probability of 93% of a WPV-free Africa in the absence of any new WPV cases reported by the end of 2015,[33] shortly before the WPV reemerged.[13] Contrasting with all other modeling studies, a recent study [34] suggested a relatively high probability of undetected circulation after more than 3 years without any polio cases in small populations, although a correction to that analysis emphasized the unrealistic nature of one of the assumptions.[35] Remarkably, the analysis reported that closed populations of 10,000 people or fewer could support many years of transmission in the absence of vaccination, and experience gaps between polio cases of over 5 years.[34] A reanalysis of this hypothetical model identified issues with the analysis and its framing, and reported results consistent with the prior literature after correcting for some errors.[36]

For peer review only - http://bmjopen.bmj.com/site/about/guidelines.xhtml

Although the modeling results demonstrated the critical importance of sustaining high population immunity through immunization programs and high-quality surveillance to obtain high confidence about no undetected circulation, the current GPEI strategic plan only covers 2013-2018,[6] which leads to uncertainty about the ability to sustain high program performance after 2018. As of mid-2018, questions continue to arise about when the GPEI will cease to exist and what resources will be available to support the polio endgame, including the certification of eradication of WPV1 and WPV3 with high confidence. The GPEI partners already began transition planning, and this process already led to some downsizing of national poliovirus programs, including the reduction of some AFP surveillance activities.[37] Thus, while the prior modeling assumed strong GPEI and national polio program performance up through the end of the polio endgame, this assumption now appears optimistic, and further analyses that explore the impact of lower quality surveillance may prove useful in the context of global certification decisions for WPV1 and WPV3 eradication. Further motivation for developing models to support certification decisions comes from the re-appearance of WPV1 in security-compromised areas in Borno, Nigeria after apparent interruption, which raised questions about the ability of poliovirus circulation without detection in communities not (or poorly) accessed by immunization and surveillance efforts within larger populations with high immunity and good surveillance.

This study aims to support future decisions about WPV certification by: (1) informing confidence about the absence of circulation by modeling the role of hard-to-reach populations, (2) examining the minimum population size required to sustain poliovirus transmission, and (3) developing a conceptual framework to provide some structure for future certification decisions,.

Methods

To inform confidence about the absence of circulation by modeling the role of hard-to-reach populations, we explored the impact of key assumptions using an existing model of a hypothetical population comprised of a well-vaccinated general population and an under-vaccinated subpopulation.[38] Table 1 lists the model inputs used to characterize this hypothetical population and explore the role of key assumptions (see appendix for model details). To explore

For peer review only - http://bmjopen.bmj.com/site/about/guidelines.xhtml

different population characteristics, we varied the total population size, the size of the under-vaccinated subpopulation, and the degree of mixing between the under-vaccinated and general population around a base case indicated by the bold values in Table 1. In addition, for each variation around the base case, we simultaneously varied the routine immunization coverage and detection probability per polio case in the under-vaccinated subpopulation. We interpret the total hypothetical population as one epidemiological block (e.g., a country) and therefore compute the confidence about no circulation based on all detections that occur in the general population and under-vaccinated subpopulation combined. However, we fix the detection probability in the general population at 95% to characterize high-quality national surveillance while considering lower detection probabilities only in the under-vaccinated subpopulation (Table 1).[38] To estimate the confidence about no circulation in this conceptual model, we use a simplified version (see appendix) of the stochastic approach developed by Eichner and Dietz (1996) [20] and adopted by others.[25-27] We define the probability of undetected circulation after a given period of *t* months without a detection as the number of times in multiple stochastic simulations that *t* months went by without a detection despite continued circulation, divided by the total number of times that *t* months went by without a detection (i.e., with or without continued circulation). Intuitively, the fraction of all time periods of *t* months without a detection but with transmission still ongoing should decrease as *t* increases, corresponding to an increasing probability of no circulation. Confidence about no circulation equals one minus the probability of undetected circulation. To visualize the impact of varying the model inputs, we focus on the time without a detection until the confidence about no circulation first exceeds 95% (CNC95%).

We revisit the question of silent transmission in small populations [22 34 36] using the hypothetical population model [38] in an attempt to inform the bounds on the maximum possible CNC95%. To do so, we ignore the general population and effectively assume a completely-isolated and unvaccinated subpopulation and otherwise adopt the hypothetical population assumptions from Table 1. We transform the DEB model to a stochastic form using the Gillespie algorithm,[39] as described elsewhere, [27] and start either at the endemic equilibrium [34] or with a single infection in an entirely susceptible population. Instead of modeling die-out using the transmission threshold,[2 27] we allow transmission to continue until the infection prevalence becomes 0. This complements the existing work [22 34 36] by providing a comparison to the same situation with a

For peer review only - http://bmjopen.bmj.com/site/about/guidelines.xhtml

more comprehensive model for poliovirus transmission,[2] adding consideration of the impact of the initial conditions, and adding the impact on confidence about no circulation.

Finally, recognizing the complexity and inter-related nature of certification decisions, we developed an influence diagram to relate certification timing decisions to outcomes. The diagram provides a conceptual framework to support certification decisions and formulate decisions about the timing of certification as an optimization problem. The diagram uses conventions from causal loop diagrams [40] and specifies the directionality of relationships between variables using unidirectional arrows. The polarity or sign at the arrow head indicates whether increasing the variable at the base of the arrow increases (+) or decreases (-) the variable that the arrow points to with all else being equal.

Patient and Public Involvement

This survey did not involve patients or public opportunities for engagement.

Results

Figure 1 illustrates how the confidence about no circulation increases with time after the last detection as a function of the surveillance quality in the under-vaccinated subpopulation (i.e., the detection probability). Clearly, higher confidence implies the need to wait longer after the last detected case, and lower detection probabilities further increase the time required to reach a certain level of confidence (e.g., the 95% line). Figure 1 shows a relatively modest effect of the detection probability in the under-vaccinated subpopulation for this hypothetical model due to continued occurrence of cases in the general population for the assumed degree of mixing (see appendix).

Figure 2 shows the CNC95% values as a function of coverage and detection probability for the under-vaccinated subpopulation. The figure shows longer times required to reach CNC95% values with increasingly more isolated under-vaccinated subpopulations (left column, top to bottom), with decreasing relative sizes of the under-vaccinated subpopulation (middle column,

For peer review only - http://bmjopen.bmj.com/site/about/guidelines.xhtml

top to bottom), and decreasing absolute sizes of a fully-isolated under-vaccinated subpopulation (right column, top to bottom, note increased y-axis ranges). The panels in Figure 2 omit curves for coverage values that do not result in eradication, because they do not allow for calculation of any confidence about eradication. The panels also omit the data point for 0 detection probability in the event of a fully-isolated under-vaccinated subpopulation, because that would imply no ability to detect the virus. Consistent with previous findings,[27] all panels in Figure 2 show higher CNC95% values with higher coverage in the under-vaccinated subpopulation. In each panel, the lowest shown coverage value may result in the longest period of undetected circulation before interruption and therefore result in the longest time to achieve high confidence about no circulation.

Looking more closely at the differences between the columns, the left column of Figure 2 shows a very strong influence of the degree of isolation on the CNC95%. With little isolation and no surveillance in the under-vaccinated subpopulation, the general population with high surveillance quality can still detect transmission because of relatively frequent spillover of polio cases (see appendix). Thus, the results do not depend much on the detection probability in the under-vaccinated subpopulation for p_{within}=0.8. In contrast, for a fully isolated under-vaccinated subpopulation (p_{within}=1), the detection probability in this population becomes a more important driver of the CNC95% than the coverage (i.e., for detection probability of 0.1 or very poor surveillance and all other inputs at the base case, the CNC95% equals almost 6 years regardless of coverage). The middle column of Figure 2 shows CNC95% values of approximately 5 years with no surveillance in a relatively small under-vaccinated subpopulation. Although the relative size of the under-vaccinated subpopulation affects the mixing dynamics and incidence of cases in both populations, much of the observed effect comes from the implied change in the absolute size of the under-vaccinated subpopulation, which directly affects the typical time between cases. As shown in the right column of Figure 2, changing the absolute size of the under-vaccinated subpopulation in the event of full isolation from the general population and a detection probability of 0.1 dramatically affects the CNC95%, which ranges from slightly over 2 years for 500,000 people to approximately 9 years for 50,000 people (i.e., a 4-fold increase in CNC95% for a 10-fold increase in population size).

For peer review only - http://bmjopen.bmj.com/site/about/guidelines.xhtml

Considering the relatively high CNC95% observed for small, isolated populations in Figure 2, Figure 3a uses a stochastic model to show the distribution of the duration of circulation in a single population not reached by vaccination at all. Figure 3a shows the results as a function of population size for a model initialized at the endemic equilibrium. For very small population sizes (e.g., hundreds), not surprisingly poliovirus infections typically die-out within a year, with a maximum duration of circulation of one year and 4 months for a closed population of 1,000 people (based on 10,000 iterations). The maximum duration of circulation increases rapidly for larger populations. For a population of 5,000 people, circulation continues for 3 or more years in 50 of 10,000 (0.5%) iterations. With population sizes of 10,000, 20,000, 30,000, 40,000 and 50,000, circulation continues for at least 10 years for 3%, 34%, 63%, 79%, and 88% of iterations, respectively.

Figure 3b shows the same analysis as Figure 3a except that it changes the initial conditions by assuming a population with no prior exposure to any polioviruses. In this context, a single introduction rapidly burns through the entire susceptible population and quickly exhausts susceptible individuals, leading to die-out and a maximum duration of circulation of less than 2 years for all population sizes considered in Figure 3b. Together, Figures 3a-b encompass the bounds on the possible duration of circulation for different initial conditions. In reality, small, completely isolated populations are unlikely to remain at the endemic equilibrium because of random fluctuations in the incidence, seasonality, and die-out, and no completely naïve populations likely exist. In a separate analysis using the same model, we verified that the addition of seasonality decreases the typical duration of circulation and increases the probability of eradication within 5 years. For example, for a population size of 20,000 people, the probability of eradication within 5 years increased from approximately 64% without seasonality to 78%-92% with a seasonal amplitude of 10% (applied to the basic reproduction number of 10), depending on the timing of the seasonal peak.

While Figure 3 implies that increasing the population size results in an increasing probability of persistent circulation (i.e., a greater probability of sustained undetected transmission), Figure 2 implies that increasing population size decreases the typical time interval between cases (i.e., lower probabilities of sustained undetected circulation). Figure 4 shows the net effect of these

For peer review only - http://bmjopen.bmj.com/site/about/guidelines.xhtml

two opposing trends and suggests that an optimal population size exists around 20,000 people. For smaller population sizes, continued transmission becomes exceedingly unlikely (Figure 3), while for larger population sizes, undetected circulation becomes less likely due to the higher frequency of cases. This non-linear behavior suggests a maximum CNC95% of approximately 2.5 years for a detection probability of 1, although the maximum increases to up to 9 years for a very low detection probability of 0.1 and a population size of 20,000 to 30,000 people.

Figure 5 shows how the desired confidence about no circulation may influence certification timing and key health economic outcomes (see appendix for details). Earlier certification and OPV cessation may increase the risk of undetected circulation after OPV cessation (and therefore the possibility of needing to restart OPV use) but may decrease the costs until OPV cessation (and therefore the overall global costs for planned polio immunization). Therefore, the fundamental optimization problem consists of finding the desired confidence about no WPV circulation at OPV cessation that minimizes the resulting total financial and societal costs. Figure 5 also shows that the costs and risks both depend on the GPEI budget until and after OPV cessation, with a lower budget saving costs in the short term but increasing the time of OPV cessation at a given confidence level and the risks of OPV restarts, which may ultimately result in greater overall costs. Optimization of the desired confidence about no WPV circulation depends critically on how the confidence about no circulation increases with time after the last detected event from the surveillance system.

Discussion

Hard-to-reach subpopulations may play a key role in deliberations about WPV circulation and decisions about WPV certification. The timing of WPV certification and subsequent OPV cessation involves high stakes and largely depends on the desired confidence about the absence of circulation. Surveillance quality emerges as a key factor that affects both the confidence about the absence of circulation and the ability to detect and control any outbreaks after OPV cessation. However, national surveillance indicators may not suffice to measure the overall surveillance system quality because gaps in surveillance at the level of tens of thousands of people may influence confidence. Our modeling suggests that high quality surveillance suffices

For peer review only - http://bmjopen.bmj.com/site/about/guidelines.xhtml

to detect transmission in the context of a relatively well-mixed under-vaccinated subpopulation (e.g., in Pakistan and Afghanistan),[41] while local gaps may miss transmission for several years in the context of highly-isolated under-vaccinated subpopulations. With respect to global certification of WPV eradication, this implies a need to address any such gaps in isolated populations that experienced WPV transmission during the last decade. The recent experience in Borno and previously in Chad and Sudan demonstrated the ability of WPVs to circulate undetected for many years in sub-populations missed by both surveillance and immunization efforts.[12 13] However, one of the main contributions of this work is that is shows that very small, isolated subpopulations cannot sustain transmission indigenously, while in the context of even very limited surveillance, persistent undetected transmission becomes increasingly unlikely for increasing population sizes. To our knowledge, the existence of a worst-case population size for undetected circulation has not yet been demonstrated for polioviruses. Our analysis confirms that with high-quality surveillance, 3 years without a detected WPV case suffices to attain high confidence about no circulation for serotype 1, even considering possible persistence in very small population sizes.

Explicit consideration of the decision to certify WPV eradication (Figure 5) suggests that if we remain confident that we can prevent the need to restart OPV due to uncontrolled outbreaks resulting from a possible WPV reemergence, then we should accept a lower confidence about the absence of circulation to certify sooner, because the costs of delaying OPV cessation would outweigh the risk of premature certification. Earlier OPV cessation particularly represents the best option if diminishing GPEI financial and/or global OPV supply resources limit our ability to maintain population immunity and/or respond effectively to post-cessation outbreaks. However, this choice depends on a willingness to accept the reputational risk of finding out that WPV still circulates despite its certification. With WPV3 not detected anywhere since 2012 [11] and in many places for decades, the confidence about no WPV3 circulation continues to grow. Although confidence about no circulation increases more slowly for WPV3 than WPV1 due to the lower PIR,[25 27] assuming 1-2 years to prepare for coordinated global OPV cessation, starting the process of removing serotype 3 OPV now would imply at least 7 years of no detection since the last WPV3 case and synchronized cessation of serotype 3 OPV use (i.e., 2012 to 2019-2020). The transition of GPEI resources already occurring leads to expected decreases in population

For peer review only - http://bmjopen.bmj.com/site/about/guidelines.xhtml

immunity for serotype 3 in some areas. Combined with on-going serotype 3 vaccine-associated paralytic poliomyelitis, this should motivate careful consideration of the costs, benefits, and risks of globally certifying WPV3 eradication and synchronizing serotype 3 OPV cessation before completing WPV1 eradication and serotype 1 OPV cessation, which now appears at least 4 years away.

Our results related to minimum population sizes appear consistent with a prior study [22] that found an average of approximately 5 years of circulation for a population of 20,000 people in a high-R_0 setting and an exponential increase in the average duration of circulation with increasing population size. The prior study also reported a higher probability of virus persistence as the degree of mixing between subpopulations increases.[22] Our study suggests that more mixing between subpopulations may not lead to a higher probability of undetected circulation because surveillance can more easily detect persistent viruses for higher degrees of mixing. Using a more realistic model than another prior analysis,[36] we similarly do not find a high probability of persistent transmission for populations of 10,000 people or less.

Like all models, our model makes simplifying assumptions that affect its behavior.[2] Specifically, we characterized a stylized, hypothetical population to systematically explore key assumptions, used a simplified semi-stochastic approach to compute CNC95% that does not fully account for all stochastic variability, and deterministically characterized die-out. However, for the analysis of small population sizes that depend most on stochastic variability, we accounted for stochastic variability and die-out at the individual level.

While this study highlights the importance of ensuring high surveillance quality in all subpopulations, it also reiterates the role of immunization in accelerating confidence about no circulation after the last detection.[27] Achieving and maintaining high population immunity to transmission represents a mission critical component of the GPEI. Populations with immunity near the threshold experience increased risk of prolonged undetected transmission. Thus, if ensuring high-quality surveillance in all subpopulations remains an elusive goal, then achieving better coverage in those subpopulations would still result in higher confidence about no

For peer review only - http://bmjopen.bmj.com/site/about/guidelines.xhtml

circulation. In contrast, high quality surveillance in the context of poor immunization still leaves the population and the world at risk.

Poliovirus environmental surveillance can detect polioviruses even in the absence of symptomatic polio cases [42 43] and offers the potential to fill some local gaps in symptomatic poliovirus surveillance. However, despite the potential for high sensitivity of environmental surveillance to detect infected individuals excreting into the catchment area, its sensitivity remains zero outside of the catchment area and depends on sampling frequency (e.g., one sample every year provides little increase in confidence over AFP alone).[44] Environmental surveillance system designs generally depend on access to a centralized sewage network,[43] which hard-to-reach subpopulations (i.e., those most likely to sustain undetected poliovirus transmission) may not possess. Further research should help to explore the ability of environmental surveillance to increase confidence about no circulation in specific areas, and the value of the information obtained from environmental surveillance relative to its costs requires evaluation.

List of abbreviations: AFP, acute flaccid paralysis; CNC95%, Time until the confidence about no circulation reaches 95%; cVDPV, circulating VDPV DEB, differential-equation based; GPEI, Global Polio Eradication Initiative; IPV, inactivated poliovirus vaccine; OPV, oral poliovirus vaccine; PIR, paralysis-to-infection ratio; VDPV, vaccine-derived poliovirus; WPV(1,2,3), wild poliovirus (of serotype 1, 2, 3, respectively)

DECLARATIONS

Authors' contributions

All authors (RDT, DAK, KMT) contributed to the study design, model development, interpretation of results, manuscript writing, and revisions. The first and second authors (RDT, DAK) performed the modeling and analyses, and the last author (KMT) secured the funding for the study.

Ethics approval and consent to participate

Not applicable

For peer review only - http://bmjopen.bmj.com/site/about/guidelines.xhtml

Consent to publish

Not applicable

Competing interests

None

Funding

This work was funded by the Bill and Melinda Gates Foundation [OPP1129391].

Acknowledgments

We thank the Bill and Melinda Gates Foundation for supporting the completion of this work [OPP1129391].

Data sharing statement

Technical appendix available on request from the authors.

References

1. World Health Assembly. Global eradication of poliomyelitis by the year 2000 (resolution 41.28). Geneva: World Health Organization, 1988.
2. Duintjer Tebbens RJ, Pallansch MA, Kalkowska DA, et al. Characterizing poliovirus transmission and evolution: Insights from modeling experiences with wild and vaccine-related polioviruses. *Risk Anal* 2013;23(4):703-49.
3. Thompson KM, Pallansch MA, Duintjer Tebbens RJ, et al. Modeling population immunity to support efforts to end the transmission of live polioviruses. *Risk Anal* 2013;33(4):647-63.
4. Thompson KM, Kalkowska DA, Duintjer Tebbens RJ. Managing population immunity to reduce or eliminate the risks of circulation following the importation of live polioviruses *Vaccine* 2015;33(3):1568-77.
5. Thompson KM, Duintjer Tebbens RJ. The case for cooperation in managing and maintaining the end of poliomyelitis: Stockpile needs and coordinated OPV cessation. *Medscape J Med* 2008;10(8):190.
6. World Health Organization. Global Polio Eradication Initiative: Polio Eradication and Endgame Strategic Plan (2013-2018). Geneva, 2013.
7. World Health Assembly. Poliomyelitis: mechanism for management of potential risks to eradication (resolution 61.1). Geneva: World Health Organization, 2008.
8. Global Polio Eradication Initiative. Global eradication of wild poliovirus type 2 declared 2015 [updated September 20, 2015. Available from:

For peer review only - http://bmjopen.bmj.com/site/about/guidelines.xhtml

http://www.polioeradication.org/mediaroom/newsstories/Global-eradication-of-wild-poliovirus-type-2-declared/tabid/526/news/1289/Default.aspx accessed November 30 2015.

9. Hampton LM, Farrell M, Ramirez-Gonzalez A, et al. Cessation of trivalent oral poliovirus vaccine and introduction of inactivated poliovirus vaccine - Worldwide, 2016. *MMWR Morb Mortal Wkly Rep* 2016;65(35):934-38. doi: 10.15585/mmwr.mm6535a3

10. World Health Organization. Polio this week as of 3 January 2018 2018 [Available from: http://polioeradication.org/polio-today/polio-now/this-week/ accessed January 10 2018.

11. Kew OM, Cochi SL, Jafari HS, et al. Possible eradication of wild poliovirus type 3--worldwide, 2012. *MMWR Morb Mortal Wkly Rep* 2014;63(45):1031-33.

12. World Health Organization. Laboratory surveillance for wild and vaccine-derived polioviruses, January 2004-June 2005. *Wkly Epidemiol Rec* 2005;80(39):335-40.

13. Nnadi C, Damisa E, Esapa L, et al. Continued endemic wild poliovirus transmission in security-compromised areas - Nigeria, 2016. *MMWR Morb Mortal Wkly Rep* 2017;66(7):190-93. doi: 10.15585/mmwr.mm6607a2

14. Nathanson N, Kew OM. From emergence to eradication: the epidemiology of poliomyelitis deconstructed. *Am J Epidemiol* 2010;172(11):1213-29. [published Online First: 2010/10/28]

15. World Health Organization. Transmission of wild poliovirus type 2 - Apparent global interruption. *Wkly Epidemiol Rec* 2001;76:95-97.

16. Deshpande JM, Nadkarni SS, Siddiqui ZA. Detection of MEF-1 laboratory reference strain of poliovirus type 2 in children with poliomyelitis in India in 2002 & 2003. *Indian Journal of Medical Research* 2003;118:217-23.

17. Duizer E, Ruijs WL, van der Weijden CP, et al. Response to a wild poliovirus type 2 (WPV2)-shedding event following accidental exposure to WPV2, the Netherlands, April 2017. *Euro Surveill* 2017;22(21):pii=30542. doi: 10.2807/1560-7917.ES.2017.22.21.30542

18. Centers for Disease Control and Prevention. Certification of poliomyelitis eradication--the Americas, 1994. *MMWR Morb Mortal Wkly Rep* 1994;43(39):720-2.

19. Debanne SM, Rowland DY. Statistical certification of eradication of poliomyelitis in the Americas. *Mathematical Biosciences* 1998;150(1):83-103.

20. Eichner M, Dietz K. Eradication of poliomyelitis: when can one be sure that polio virus transmission has been terminated? *Am J Epidemiol* 1996;143(8):816-22.

21. Eichner M, Hadeler KP. Deterministic models for the eradication of poliomyelitis: vaccination with the inactivated (IPV) and attenuated (OPV) polio virus vaccine. *Mathematical Biosciences* 1995;127(2):149-66.

22. Eichner M, Hadeler KP, Dietz K. Stochastic models for the eradication of poliomyelitis: minimum population size for polio virus persistence. In: Isham V, Medley GF, eds. Models for Infectious Human Diseases: their structure and relation to data. New York: Cambridge University Press 1996:315-27.

23. World Health Organization. Global Polio Eradication Initiative: Strategic plan 2004-2008. Geneva, 2004.

24. Bahl S, Kumar R, Menabde N, et al. Polio-free certification and lessons learned - South-East Asia region, march 2014. *MMWR Morb Mortal Wkly Rep* 2014;63(42):941-46.

For peer review only

25. Kalkowska DA, Duintjer Tebbens RJ, Thompson KM. The probability of undetected wild poliovirus circulation after apparent global interruption of transmission. *Am J Epidemiol* 2012;175(9):936-49.
26. Houy N. The probability of undetected wild poliovirus circulation: Can we do better? *Journal of Theoretical Biology* 2015;382:272-78.
27. Kalkowska DA, Duintjer Tebbens RJ, Pallansch MA, et al. Modeling undetected live poliovirus circulation after apparent interruption of transmission: Implications for surveillance and vaccination. *BMC Infect Dis* 2015;15(66):1. doi: 10.1186/s12879-015-0791-5
28. Kalkowska DA, Duintjer Tebbens RJ, Thompson KM. Modeling strategies to increase population immunity and prevent poliovirus transmission in two high-risk areas in northern India. *J Infect Dis* 2014;210(Suppl 1):S398-S411.
29. Kalkowska DA, Duintjer Tebbens RJ, Thompson KM. Modeling strategies to increase population immunity and prevent poliovirus transmission in the high-risk area of northwest Nigeria. *J Infect Dis* 2014;210(Suppl 1):S412-S23.
30. Duintjer Tebbens RJ, Kalkowska DA, Wassilak SGF, et al. The potential impact of expanding target age groups for polio immunization campaigns. *BMC Infect Dis* 2014;14:45.
31. Kalkowska DA, Duintjer Tebbens RJ, Grotto I, et al. Modeling options to manage type 1 wild poliovirus imported into Israel in 2013. *J Infect Dis* 2015;211(11):1800-12.
32. Berchenko Y, Manor Y, Freedman LS, et al. Estimation of polio infection prevalence from environmental surveillance data. *Science translational medicine* 2017;9(383) doi: 10.1126/scitranslmed.aaf6786
33. Famulare M. Has wild poliovirus been eliminated from Nigeria? . *PLoS One* 2016;10:e0135765.
34. Vallejo C, Keesling J, Koopman JS, et al. Silent circulation of poliovirus in small populations. *Infectious Disease Modelling* 2017;2(4):431-40. doi: 10.1016/j.idm.2017.11.001
35. Vallejo C, Keesling J, Koopman JS, et al. Corrigendum to "Silent circulation of poliovirus in small populations" (in press). *Infectious Disease Modelling* 2018 doi: 10.1016/j.idm.2017.11.001
36. Kalkowska DA, Duintjer Tebbens RJ, Thompson KM. Another look at silent circulation of poliovirus in small populations (in preparation).
37. World Health Organization. Report on polio transition planning - advance draft as of 18 April, 2017 2017 [Available from: http://www.who.int/mediacentre/events/2017/advance-report-polio-transmission.pdf?ua=1 accessed March 12 2018.
38. Thompson KM, Duintjer Tebbens RJ. Lessons from the polio endgame: Overcoming the failure to vaccinate and the role of subpopulations in maintaining transmission. *J Infect Dis* 2017;216(Suppl 1):S176-S82.
39. Gillespie DT. A general method for numerically simulating the stochastic time evolution of coupled chemical reactions. *Journal of Computational Physics* 1976;22:403-34.
40. Sterman J. Business dynamics: Systems thinking and modeling for a complex world. Boston, MA: McGraw-Hill 2000.

For peer review only - http://bmjopen.bmj.com/site/about/guidelines.xhtml

41. Duintjer Tebbens RJ, Pallansch MA, Cochi SL, et al. Modeling poliovirus transmission in Pakistan and Afghanistan to inform vaccination strategies in undervaccinated subpopulations. *Risk Anal* 2018 doi: 10.1111/risa.12962
42. Global Polio Eradication Initiative. Guidelines on environmental surveillance for detection of polioviruses - working draft - March 2015, 2015.
43. Duintjer Tebbens RJ, Zimmermann M, Pallansch MA, et al. Insights from a systematic search for information on designs, costs, and effectiveness of poliovirus environmental surveillance systems. *Food and Environmental Virology* 2017;9(4):361-82. doi: 10.1007/s12560-017-9314-4
44. Kalkowska DA, Duintjer Tebbens RJ, Thompson KM. Environmental surveillance impacts on confidence about no undetected serotype 1 wild poliovirus circulation in Pakistan and Afghanistan (in preparation).
45. Duintjer Tebbens RJ, Thompson KM. Costs and benefits of including inactivated in addition to oral poliovirus vaccine in outbreak response after cessation of oral poliovirus vaccine use. *Medical Decision Making Policy & Practice* 2017;2:1-13.
46. Kew O, Morris-Glasgow V, Landaverde M, et al. Outbreak of poliomyelitis in Hispaniola associated with circulating type 1 vaccine-derived poliovirus. *Science* 2002;296(5566):356-59.
47. Wassilak SGF, Pate MA, Wannemuehler K, et al. Outbreak of type 2 vaccine-derived poliovirus in Nigeria: emergence and widespread circulation in an underimmunized population. *J Infect Dis* 2011;203(7):898-909. [published Online First: 2011/03/16]
48. Más Lago P, Cáceres VM, Galindo MA, et al. Persistence of vaccine-derived poliovirus following a mass vaccination campaign in Cuba: implications for stopping polio vaccination after global eradication. *International Journal of Epidemiology* 2001;30:1029-34.
49. Alexander LN, Seward JF, Santibanez TA, et al. Vaccine policy changes and epidemiology of poliomyelitis in the United States. *JAMA* 2004;292(14):1696-701.
50. Patriarca PA, Wright PF, John TJ. Factors affecting the immunogenicity of oral poliovirus vaccine in developing countries: review. *Rev Infect Dis* 1991;13:926-39.
51. Duintjer Tebbens RJ, Pallansch MA, Wassilak SGF, et al. Characterization of outbreak response strategies and potential vaccine stockpile needs for the polio endgame. *BMC Infect Dis* 2016;16:137. doi: 10.1186/s12879-016-1465-7
52. Duintjer Tebbens RJ, Pallansch MA, Chumakov KM, et al. Review and assessment of poliovirus immunity and transmission: Synthesis of knowledge gaps and identification of research needs. *Risk Anal* 2013;33(4):606-46.
53. Duintjer Tebbens RJ, Pallansch MA, Chumakov KM, et al. Expert review on poliovirus immunity and transmission. *Risk Anal* 2013;33(4):544-605.
54. Duintjer Tebbens RJ, Pallansch MA, Kew OM, et al. A dynamic model of poliomyelitis outbreaks: Learning from the past to help inform the future. *Am J Epidemiol* 2005;162(4):358-72.
55. Horstmann DM, Paul JR. The incubation period in human poliomyelitis and its implications. *JAMA* 1947;135(1):11-4.
56. Population Division of the Department of Economic and Social Affairs of the United Nations Secretariat. World Population Prospects. The 2012 revision. Volume I: Comprehensive tables, 2013.

For peer review only - http://bmjopen.bmj.com/site/about/guidelines.xhtml

57. Hampton LM, du Châtelier GM, Fournier-Caruana J, et al. Considerations for the full global withdrawal of oral polio vaccine after eradication of polio. *J Infect Dis* 2017;216(Suppl 1):S217-S25.
58. Global Polio Eradication Initiative. Financing 2018 [Available from: http://polioeradication.org/financing/ accessed February 12 2018.
59. Duintjer Tebbens RJ, Pallansch MA, Cochi SL, et al. An economic analysis of poliovirus risk management policy options for 2013-2052. *BMC Infect Dis* 2015;15:389. doi: 10.1186/s12879-015-1113-7
60. Platt LR, Estivariz CF, Sutter RW. Vaccine-associated paralytic poliomyelitis: a review of the epidemiology and estimation of the global burden. *J Infect Dis* 2014;210(Suppl 1):S380-89.
61. Thompson KM, Duintjer Tebbens RJ. National choices related to inactivated poliovirus vaccine, innovation, and the end game of global polio eradication. *Exp Rev Vaccines* 2014;13(2):221-34.
62. World Health Organization. Global circulating vaccine-derived poliovirus cases, 2000-2017 2018 [updated February 6, 2018. Available from: http://polioeradication.org/polio-today/polio-now/this-week/circulating-vaccine-derived-poliovirus/ accessed February 12 2018.
63. de Gourville EM, Sangrujee N, Duintjer Tebbens RJ, et al. Global Surveillance and the value of information: The case of the global polio laboratory network. *Risk Anal* 2006;26(6):1557-69.
64. Duintjer Tebbens RJ, Thompson KM. Comprehensive screening for immunodeficiency-associated vaccine-derived poliovirus: an essential OPV cessation risk management strategy. *Epidemiology and Infection* 2017;145(2):217-26. doi: 10.1017/S0950268816002302

For peer review only

For peer review only - http://bmjopen.bmj.com/site/about/guidelines.xhtml

Table 1: Model inputs to characterize a hypothetical population that contains an under-vaccinated subpopulation.

Model input	Value(s)[a]	Source/notes
Total population size	500,000;**1 million**;5 million	No effect on DEB model behavior, but required for stochastic analysis of detections
Time until vaccination starts, years<br>General population<br>Under-vaccinated subpopulation	 30<br>40	Assumption to characterize hard-to-reach subpopulation within well-vaccinated general population
Initial age distribution<br>0-2 months<br>3-59 months<br>5-14 years<br>≥ 15 years	 0.01<br>0.15<br>0.25<br>0.59	Equilibrium age distribution[38]
Birth rate, births/person/year	0.02	[38]
Death rate, deaths/person/year	0.02	[38]
Basic reproduction number (R_0)	10	[38]
Proportion of transmissions via oropharyngeal route	0.3	[38]
Proportion of contacts reserved for individuals within the same mixing age group	0.4	Same value as used in [38] (not explicitly listed)
Average per-dose take rate for serotype 1 OPV	0.6	Increased from 0.5 to maintain similar coverage thresholds with different run-up [38]
Routine immunization coverage<br>General population<br>Under-vaccinated subpopulation	 0.95<br>0.75;0.82;0.85;0.90;0.95[b]	Represents coverage with exactly 3 OPV doses; general population based on [38], under-vaccinated varied around threshold to eradicate, which equals 0.82 for the bolded values in the middle column
Proportion of contacts with under-vaccinated subpopulation (p_{within})	0.8;**0.95**;1.00	Selected values from [38]
Size of under-vaccinated subpopulation compared to total population	1/20;**1/10**;1/5	Selected values from [38]
Paralysis-to-infection ratio (PIR)	1/200	Average for serotype 1 wild poliovirus[2 14]
Detection probability per polio case<br>General population<br>Under-vaccinated subpopulations	 0.95<br>0;0.1;0.2;0.3;0.4;0.5;0.6;0.7;0.8;0.9;0.95[b]	Assumption to characterize hard-to-reach subpopulation within general population with high acute flaccid paralysis surveillance quality

Abbreviations: DEB, differential-equation based; OPV, oral poliovirus vaccine

[a] Values shown in bold represent values that we held fixed when varying other values in sensitivity analyses

[b] All values considered jointly in all sensitivity analysis (hence no single value bolded)

For peer review only - http://bmjopen.bmj.com/site/about/guidelines.xhtml

For peer review only

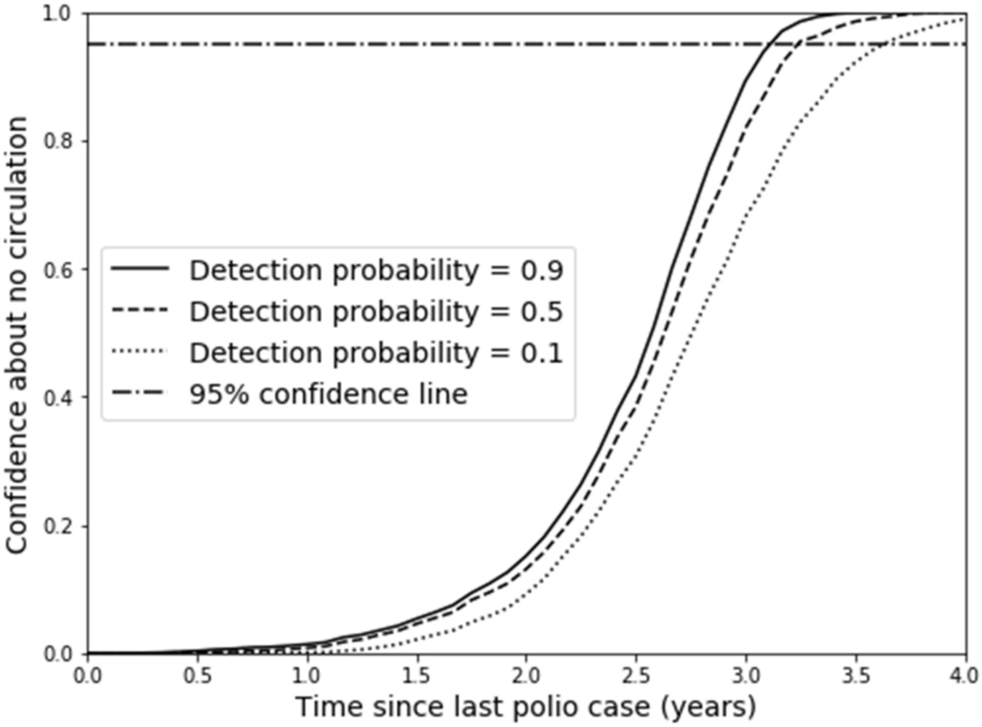

Figure 1: Confidence about no circulation as a function of time since the last detection for different detection probability values for the hypothetical model base case, with coverage at the corresponding minimum to eliminate WPV (i.e., 0.82).

179x133mm (300 x 300 DPI)

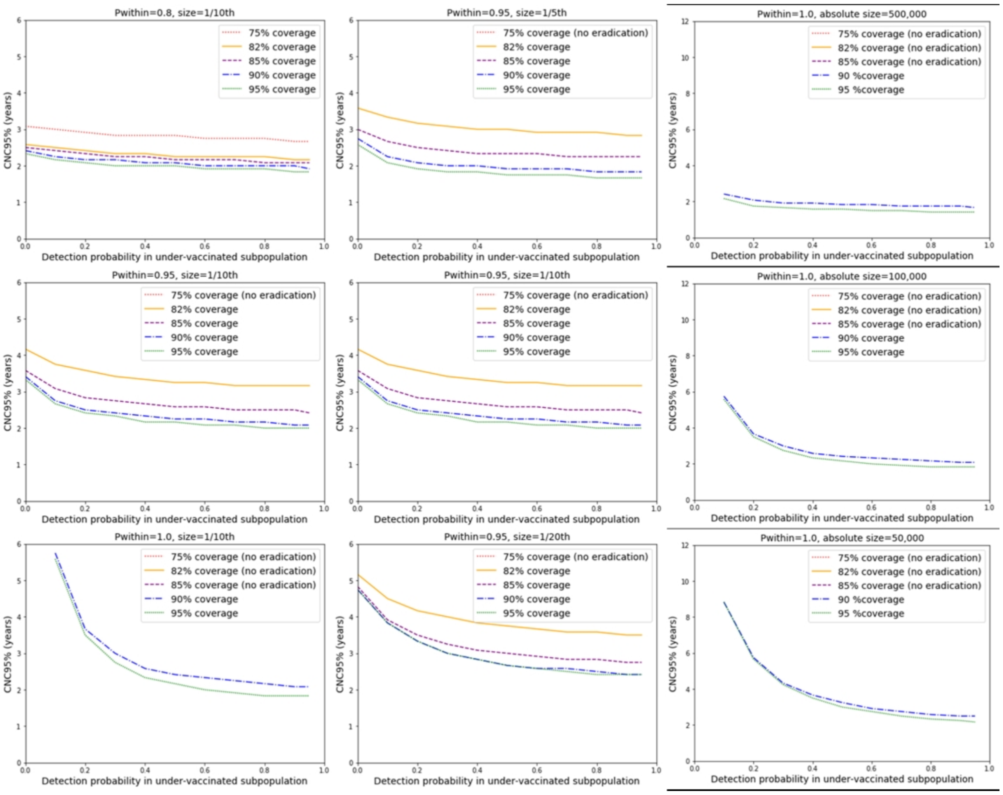

Figure 2: Time until the confidence about no circulation reaches 95% (CNC95%) from the stochastic analysis for different degrees of isolation of the under-vaccinated subpopulation (left column), relative sizes of the under-vaccinated subpopulation (middle column), and absolute sizes of a fully-isolated under-vaccinated subpopulation (right column, note doubled y-axis ranges).

250x196mm (300 x 300 DPI)

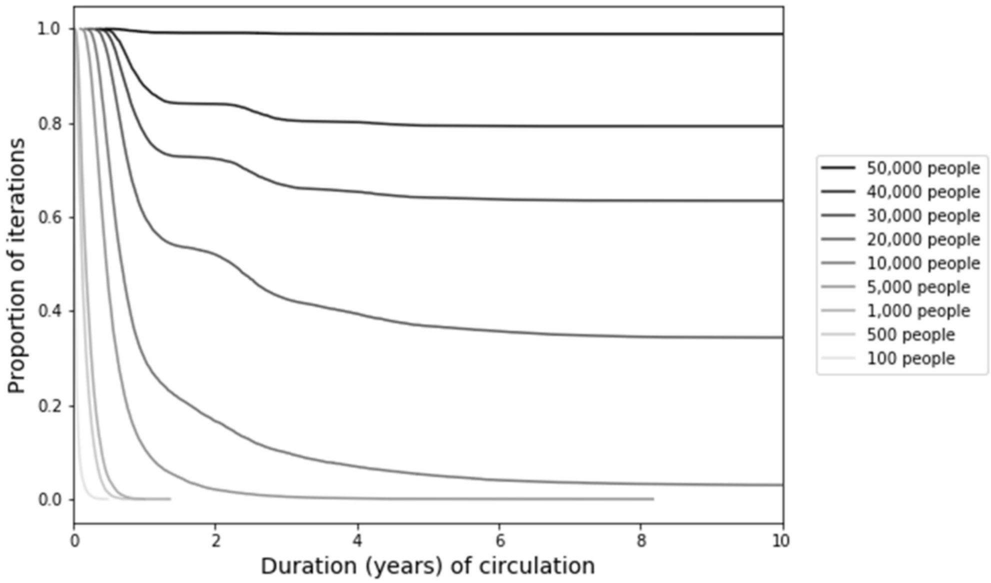

Results from the analysis of the relationship between population size and persistence of circulation of serotype 1 wild poliovirus transmission in the fully stochastic model (a) Model starts at the endemic equilibrium

222x133mm (300 x 300 DPI)

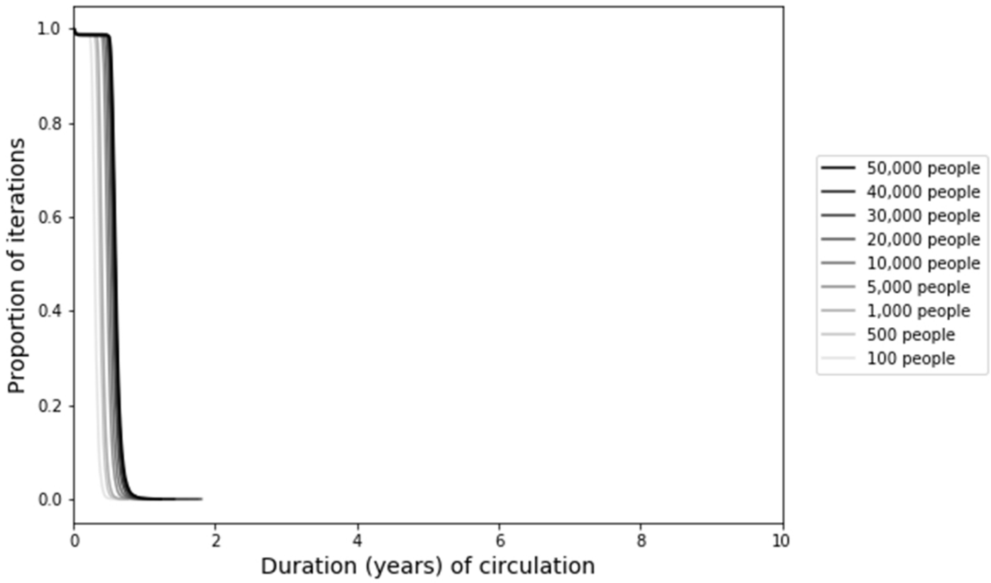

Figure 3: Results from the analysis of the relationship between population size and persistence of circulation of serotype 1 wild poliovirus transmission in the fully stochastic model (b) Model starts with a single infection in a fully susceptible population

222x133mm (300 x 300 DPI)

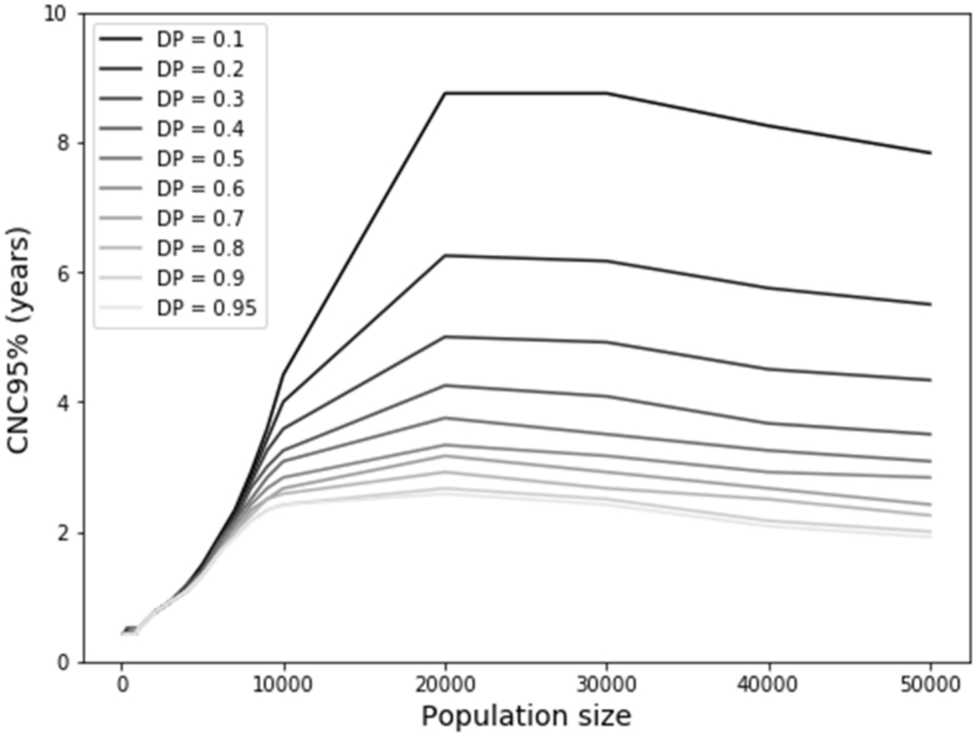

Figure 4: Time until the confidence about no circulation reaches 95% (CNC95%) for small population sizes in the fully stochastic model that starts at the endemic equilibrium, by detection probability (DP)

177x133mm (300 x 300 DPI)

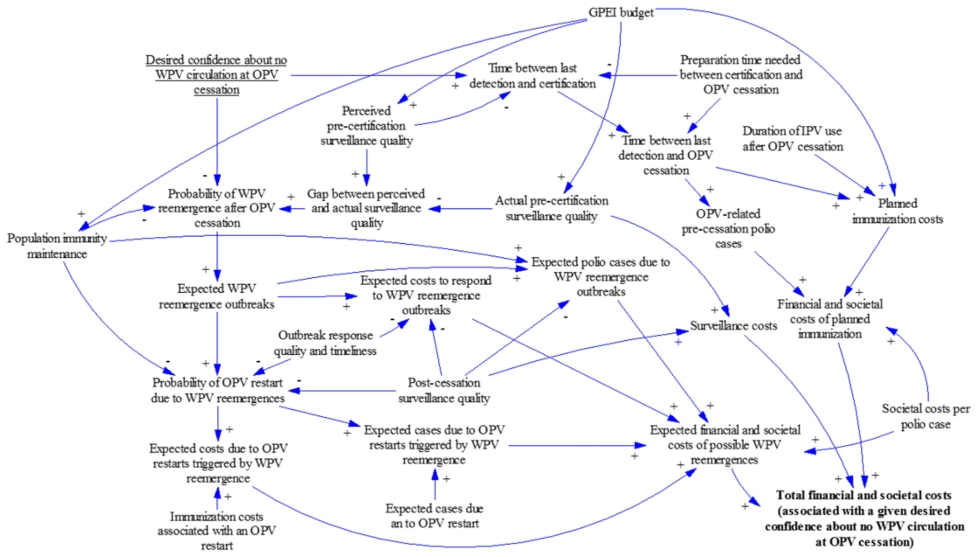

Figure 5: Conceptual diagram for the implications of choices about the timing of certification of eradication of a wild poliovirus serotype on total financial and societal costs

228x135mm (300 x 300 DPI)

APPENDIX for "Certification of global eradication: The role of hard-to-reach subpopulations and confidence about the absence of transmission"

Radboud J. Duintjer Tebbens,[1] Dominika A. Kalkowsa,[1] Kimberly M. Thompson[1]

Differential-equation based model and results

The DEB model we use to examine the role of subpopulations [38] made simplifying assumptions about what a high-risk population might look like and otherwise adopted the comprehensive structure and setting-invariant model inputs of a previously developed and calibrated differential-equation based poliovirus transmission and OPV evolution model.[2 30] The following text from the appendix of a prior publication [45] (with references renumbered) briefly describes the model and Figures A1-2 and Table A1 cited in the text provide the model structure and generic inputs (i.e., model inputs that remain the same for all populations).

"The differential equation-based poliovirus transmission and OPV evolution model (DEB model) [2] tracks the movement of people between demographic age groups (grouped into mixing age groups that mix preferentially amongst themselves), and for each serotype between oropharyngeal and intestinal infection stages (resulting in potential oropharyngeal and fecal-oral transmission, respectively), immunity states, and waning stages. Figure A1 provides an overview of the model structure based on prior work.[2] Figure A1a depicts the immunity states with the flows that move individuals in and out of them and Figure A1b details how effectively vaccinated or infected individuals progress through different stages of infection and, in the event of infection with OPV, through OPV evolution stages. The model assumes that active immunity from prior vaccination or infection results in permanent protection from polio (disease), but only partial protection from subsequent infection and participation in transmission, depending on the nature of immunity (IPV-induced vs. LPV-induced or both) and time since the last exposure (i.e. waning stage). The model includes 5 waning stages, 6 fecal-oral and 6 oropharyngeal infection stages (2 latent and 4 infectious, with varying degrees of infectiousness), and also accounts for a delay between IPV receipt and development of the immune response that moves individuals to the next IPV immunity state. In Figure A1a, we note that the model assumes identical properties for "IPV and LPV" and "≥ 2 LPV infections" and that the recent waning stages of these immunity states represent the highest degree of immunity to transmission in the model. The model further tracks OPV evolution by moving individuals infected with the OPV parent strain (stage 0) through 20 successive reversion stages that can each transmit and that come with increasing paralysis-to-infection ratios and relative basic reproduction numbers (R_0 values) compared to homotypic WPVs. The last reversion stage (stage 19) represents fully-reverted VDPVs with assumed paralysis-to-infection ratio and R_0 equivalent to homotypic WPVs. For WPVs or any OPV reversion stage, the DEB model mimics die-out by setting the force-of-infection for the given strain to 0 whenever its effective prevalence of infections resides below a calibrated threshold of 5 per million people. Consequently, OPV-related viruses can only continue to transmit and thus evolve to cVDPVs through successive infections when low enough population immunity to transmission permits circulation of the OPV viruses introduced in the population through vaccination. We fixed the die-out process, model structure, and numerical

For peer review only - http://bmjopen.bmj.com/site/about/guidelines.xhtml

model inputs that characterize them across all populations we modeled and Table A1 includes the corresponding generic model inputs. […]

“Figure A2 summarizes the results of the model calibration process, based on prior work.[2] With the generic model inputs from Table A1 fixed, we compared our model behavior against i) data on children with non-polio acute flaccid paralysis who reported no receipt of OPV for northern India (modeled separately for Western Uttar Pradesh (WUP) and Bihar) and northwest (NW) Nigeria; ii) data on polio incidence and die-out of endemic WPV transmission for all situations and serotypes (shown in Figure A2 for WPV1 and WPV3 in northern India and northwest Nigeria and for all 3 WPV serotypes in the USA); iii) data from WPV importation outbreak behavior in the Netherlands, Tajikistan, and Albania; iv) data on age distributions of cases for all situations in which meaningful data was available (shown in Figure A2 for the Netherlands, Tajikistan, and Albania); v) available serogical data on the effect of secondary OPV immunity in the USA and Cuba (not shown); vi) indigenous emergence of cVDPVs (shown in Figure A2 for northern India, NW Nigeria (both serotype 2), Haiti, and Madura in Indonesia (both serotype 1); and vii) no indigenous emergence of cVDPVs in all other situations and serotypes (die-out of serotype 1 OPV-related viruses shows in Figure A2 for Cuba and Haiti). We subsequently applied the model to successfully reproduce the asymptomatic transmission of an imported WPV1 in Israel in 2013.[31]” [45, online supplement pp. 1-2]

Most critically in the context of certification questions, the DEB model approximates interruption of live poliovirus transmission (i.e., of an OPV, WPV, vaccine-derived poliovirus (VDPV), or OPV-related strain) in a population to occur when the effective infectiousness-weighted proportion of the population infectious with that poliovirus drops below 5 per million people (i.e., the transmission threshold EPI^*).[2] While this simplifies the true die-out behavior, which depends on local heterogeneity and chance, it appears capable of generating WPV die-out times consistent with observations in a broad range of settings.[2 30 31 41] Moreover, when applied to the persistence of OPV-related viruses that evolve to fully transmissible and neurovirulent circulating VDPVs (cVDPVs), the approximation produces cVDPV outbreaks for conditions in which they occurred (e.g., in Hispaniola[46] and Nigeria[47]) and no cVDPV outbreaks for conditions in which they did not occur despite OPV use and cessation (e.g., in Cuba[48] and the USA[49]).[2]

Use of the hypothetical model clarified that under-vaccinated subpopulations can sustain poliovirus transmission independently despite high coverage in the surrounding general population and showed how the minimum coverage needed to interrupt transmission depends on the degree of isolation and the relative size of the under-vaccinated subpopulation.[38] To explore the role of hard-to-reach under-vaccinated subpopulations for certification questions, we modified the hypothetical model in two ways and added a stochastic layer on top of the DEB model to simulate polio case detections. The first modification consisted of desynchronizing the time when vaccination starts in the general and under-vaccinated subpopulations to simulate the concept of a population that remains inaccessible for an extended period of time. Specifically, we run the model, which assumes equal birth and death rates and thus no population growth (Table 1), without vaccination for 30 years to settle into the endemic equilibrium, and then instantly change the routine immunization coverage in the general population with three OPV doses to 0.95, which lies well above the threshold of 0.92 needed to interrupt transmission in a closed population with similar characteristics.[38] However, we assume that the under-vaccinated subpopulation initially remains completely unreached by vaccination, with vaccine introduction

For peer review only - http://bmjopen.bmj.com/site/about/guidelines.xhtml

in the under-vaccinated subpopulation occurring 10 years after vaccine introduction in the general population. Desynchronizing the introduction of vaccination affects the dynamics and effectively makes it more difficult to interrupt transmission after introducing vaccination in the last subpopulation. To offset this effect, we consider a different hypothetical population with a slightly higher average per-dose take rate for OPV of 0.6 instead of 0.5 in the original analysis[38] (e.g., due to lower exposure to enteric viruses that interfere with vaccine take [50]). As in the original analysis,[38] we vary the coverage in the under-vaccinated subpopulation, the relative size of the under-vaccinated subpopulation compared to the total population, and the degree of preferential mixing, characterized by the proportion of potentially infectious contacts of individuals in the under-vaccinated subpopulations with other individuals in the same subpopulation (p_{within}).

Figure A3 shows the behavior of the incidence of infections in fully susceptible individuals and infants born with maternal immunity as a function of the varied DEB model inputs. Generally, the model yields incidence proportional to population size before vaccination starts. After the introduction of vaccination with high coverage in the general population, the initially still unvaccinated subpopulation becomes the main contributor to the total incidence. However, with less than 100% coverage in the general population and some interaction between the two populations (i.e., $p_{within}<1$), some incidence continues to occur in the general population as exported viruses find unvaccinated individuals. Lower values of p_{within} imply more interaction between the two populations and result in more incidence in the general population before vaccination in the under-vaccinated subpopulation begins (middle column of Figure A3). The relative size of the under-vaccinated subpopulation also affects the extent to which the under-vaccinated subpopulation affects the general population (right column of Figure A3). With base case model inputs, the minimum coverage in the under-vaccinated subpopulation to interrupt transmission equals 0.82. Higher coverage values mean interruption occurs sooner after the introduction of vaccination in the under-vaccinated subpopulation, while lower coverage values mean that transmission continues and can eventually rebound and settle into a new equilibrium (left column in Figure A1).

While the prior approach used fully stochastic transmission models to randomly generate infections, die-out, and polio cases and detections,[19-22] for efficiency we use post-hoc processing of DEB model results to randomly generate only the times when polio cases and detections stochastically occur. Specifically, for each setting of the DEB model, we record the deterministic realization of the daily incidence of infections in fully susceptible individuals of any age and 50% of infants less than 3 months of age born with maternal immunity, which represent the only individuals at risk of becoming a polio case in the DEB model.[2] We then randomly determine the number of polio cases resulting from the infection incidence on each day using a Poisson draw with a rate equal to the infection incidence multiplied by the PIR. For each generated case, we use a separate uniform random draw to determine whether it results in a detection based on each of the detection probabilities in Table 1 (e.g., a random uniform draw of 0.45 would mean that the case results in a detection only for detection probabilities of more than 0.45). For each DEB model setting, we repeat the post-hoc stochastic process 10,000 times and we start generating cases 10 years before vaccination starts in the general population, which we assume starts vaccination 10 years earlier than the under-vaccinated subpopulation (see appendix). The precise choice of when to start randomly generating cases exerts negligible

For peer review only - http://bmjopen.bmj.com/site/about/guidelines.xhtml

influence on the results as long as it occurs before cases become rare (i.e., before the interval between cases becomes longer). For simplicity, although prior work showed the significant role of serotype differences and seasonality,[20, 22] the hypothetical model inputs reflect WPV1 and assumes no seasonality. A limitation arises from the direct scaling of the DEB model with absolute population size, such that die-out depends on the effective proportion of infectious individuals rather than the absolute number. Using the post-hoc stochastic analysis, the absolute population size affects the number of infections, which affects the typical interval between detected cases. We show that CNC95% increases substantially for smaller absolute population sizes.

Our initial findings motivated analysis of the minimum population size that can sustain WPV circulation on its own to determine whether the upper bound on the CNC95% of 9 years could occur in real populations. However, for population sizes far below 100,000, the DEB model becomes inadequate because it allows prevalence to remain above the die-out threshold even with only fractional numbers of infections (i.e., less than one infected person). Therefore, we used a fully stochastic model to explore questions of minimum population size. We run the model 10,000 times for different population sizes and initial conditions and report the distribution of the duration of circulation and the CNC95%.

Exploration of the causal interactions relevant to global WPV certification decisions with an influence diagram (Figure 5)

Table A2 provides indicative estimates of the key quantities in Figure 5, based on the literature. Figure 5 assumes that policy makers explicitly or implicitly set a *desired confidence about no WPV circulation at OPV cessation*. In reality, they may focus on the confidence at certification, but given that it takes some fixed *preparation time needed between certification and OPV cessation*, any set confidence at the time of certification corresponds to some *desired confidence about no WPV circulation at OPV cessation.* A higher desired confidence level implies a longer *time between last detection and certification.* This time decreases with increasing investments in immunization and surveillance from the *GPEI budget* through *population immunity maintenance* and the *perceived pre-certification surveillance quality*, respectively. The main drawback of a longer *time between last detection and OPV cessation* comes in the form of longer OPV use in most countries, which results in *planned immunization costs* and *OPV-related pre-cessation polio cases* (i.e., vaccine-associated paralytic polio and VDPVs). In addition, with some globally-recommended or nationally-preferred *duration of IPV after OPV cessation,* later OPV cessation would imply greater overall IPV costs, because global IPV use already started (i.e., only the end, and not the beginning of IPV use depends on the timing of cessation of the last OPV serotypes). These drawbacks together lead to *financial and societal costs of planned immunization*. This includes the monetary equivalent of the *OPV-related polio cases*, which depends on the country income-level-dependent *societal costs per polio case.*

On the left side of Figure 5, we see the benefits of setting a higher *desired confidence about no WPV circulation at OPV cessation.* A higher confidence implies a lower *probability of a WPV reemergence after OPV cessation* (all else being equal). However, this probability does not directly equal the reciprocal of the confidence in the event of a *gap between perceived and actual surveillance quality.* Specifically, if the *perceived pre-certification surveillance quality* exceeds

For peer review only - http://bmjopen.bmj.com/site/about/guidelines.xhtml

the *actual pre-certification surveillance quality*, then the true *probability of WPV reemergence after OPV cessation* equals more than 1 minus the *desired confidence about no WPV circulation at OPV cessation*, and vice versa. This potential discrepancy highlights the importance of continued assessment of surveillance quality and assurance of high surveillance quality. A lower *GPEI budget* also decreases *population immunity maintenance* and thus increases the *probability of WPV reemergence after OPV cessation*, which implies an increase in *expected WPV reemergence outbreaks.* Unlike other possible types of post-cessation outbreaks, a WPV reemergence would almost certainly occur in the most challenging populations. Any such reemergences would lead to *expected polio cases due to WPV reemergence outbreaks* and *expected costs to respond to WPV emergence outbreaks*. The expected costs and cases decrease with higher *post-cessation surveillance quality*, which affects the extent of viral spread at the time of outbreak detection (and beyond), and with a better *outbreak response quality and timeliness,* which both increase the probability of effective outbreak control.[51] However, the occurrence of any outbreaks comes with some probability of uncontrolled outbreaks, either by failing to control the original outbreak virus, or by creating new cVDPV outbreaks with the OPV vaccine used in the response. This implies some *probability of OPV restart due to WPV reemergences*, which would carry very significant *expected costs due to an OPV restart triggered by WPV reemergence* and *expected cases due to an OPV restart triggered by WPV emergence* (Table A2). For moderate or high *probability of OPV restart due to WPV reemergences*, the resulting *expected costs due to OPV restarts triggered by WPV reemergence* and *expected cases due to OPV restarts triggered by WPV reemergence* would likely dwarf the costs and cases associated with any controlled outbreaks due to WPV reemergences and would therefore drive the *expected financial and societal costs of possible WPV reemergences.*

Together with the *surveillance costs*, which act to moderate the costs of delayed OPV cessation or premature OPV cessation, the *expected financial and societal costs of possible WPV reemergences* and the *financial and societal costs of planned immunization* together make up the *total financial and societal costs (associated with any given desired confidence about no WPV circulation at OPV cessation).* The costs of possible WPV emergences and the costs of planned immunization move in opposite directions as a function of the *desired confidence about no circulation at OPV cessation.*

Figure 5 also highlights the consequences of the GPEI already scaling down some of its supplemental immunization and surveillance activities. While scaling down saves costs in the short term, doing so could lead to larger long-term costs by delaying certification and OPV cessation (i.e., requiring higher confidence about no circulation), which would imply that OPV cessation could occur in the context of lower global population immunity to transmission and lower ability to rapidly detect outbreaks. This ultimately implies an increase in the expected *total financial and societal costs (associated with any given desired confidence about no WPV circulation at OPV cessation)*. For visual simplicity, Figure 5 omitted some additional complexity involved in this decision. Furthermore, given that the confidence about no circulation increases with time after the last detection, we could have equivalently centered Figure 5 around finding the optimal time between the last detection and certification or OPV cessation. The amounts in Table A2 highlight the significant financial and humanitarian stakes involved in finding the optimal *desired confidence about no WPV circulation at OPV cessation.*

For peer review only - http://bmjopen.bmj.com/site/about/guidelines.xhtml

Table A1: Generic inputs of the DEB model[2 30] (adopted from the online supplement of Duinter Tebbens et al., 2017[45])

Model input (symbol)	Best estimate	Source
Relative susceptibility (σ) of recent immunity states (for PV1;PV2;PV3)		[52 53]
- Maternally immune	0.78;0.79;0.77	
- 1 successful IPV	0.91;0.92;0.90	
- 2 successful IPV	0.80;0.80;0.79	
- ≥ 3 successful IPV	0.72;0.72;0.71	
- 1 LPV infection	0.42;0.43;0.41	
- ≥ 2 LPV infections	0.21;0.22;0.20	
- IPV and LPV	0.21;0.22;0.20	
Duration of latent period (ξ^{fec} or ξ^{oro}, in days)	~ 3[a]	[52 53]
Duration of fecal infectiousness (γ^{fec}, in days) of recent immunity states (for PV1;PV2;PV3)		[52 53]
- Fully susceptible	28.0;27.8;28.3	
- Maternally immune	24.6;24.6;24.6	
- 1 successful IPV,	24.5;24.4;24.7	
- 2 successful IPV	21.1;20.8;21.3	
- ≥ 3 successful IPV	18.0;17.7;18.2	
- 1 LPV infection	11.6;10.5;10.5	
- ≥ 2 LPV infections	10.1;8.9;8.9	
- IPV and LPV	10.1;8.9;8.9	
Duration of oropharyngeal infectiousness (γ^{oro}, in days) of recent immunity states (no serotype differences)		[52 53]
- Fully susceptible	13.4	
- Maternally immune	11.9	
- 1 successful IPV	9.9	
- 2 successful IPV	6.6	
- ≥ 3 successful IPV	6.1	
- 1 LPV infection	5.0	
- ≥ 2 LPV infections	3.7	
- IPV and LPV	3.7	
Relative fecal infectiousness (π^{fec}) of recent immunity states (for PV1;PV2;PV3)		[52 53]
- Maternally immune	0.96;0.96;0.95	
- 1 successful IPV	0.92;0.92;0.91	
- 2 successful IPV	0.70;0.69;0.68	
- ≥ 3 successful IPV	0.61;0.59;0.59	
- 1 LPV infection	0.39;0.43;0.43	
- ≥ 2 LPV infections	0.20;0.23;0.23	
- IPV and LPV	0.20;0.23;0.23	
Relative oropharyngeal infectiousness (π^{oro}) of recent immunity states (no serotype differences)		[52 53]
- Maternally immune	0.68	
- 1 successful IPV	0.30	
- 2 successful IPV	0.17	
- ≥ 3 successful IPV	0.12	
- 1 LPV infection	0.33	
- ≥ 2 LPV infections	0.21	
- IPV and LPV	0.21	
Number of infection stages		
- Latent period (r)	2	
- Infectious period (s)	4	
Relative weight of infection stages, compared to average weight over the infectious period (θ_j, j=0,…,r+s-1)		[52 53]

For peer review only - http://bmjopen.bmj.com/site/about/guidelines.xhtml

- Infection stage 0 and 1 (latent stages)<br>- Infectious stage 2<br>- Infectious stage 3<br>- Infectious stage 4<br>- Infectious stage 5	0<br>12/17<br>40/17<br>12/17<br>4/17	
IPV immunity delay (φ, in days)	7	54
Number of waning stages (*nw*)	5	
Shape of waning function (z_w)	5	52 53
Average time to reach last waning stage (ρ, in days)<br>- Type 1&2<br>- Type 3	 4×365<br>3×365	52 53
Average time for maternal immunes to wane to fully susceptible (ρ_{MI}, in days)	0.25×365	52 53
Relative susceptibility (σ) for last waning stage (no serotype differences)<br>- 1 successful IPV<br>- 2 successful IPV<br>- ≥ 3 successful IPV<br>- 1 LPV infection<br>- ≥ 2 LPV infections<br>- IPV and LPV	 1.0<br>1.0<br>1.0<br>0.8<br>0.7<br>0.7	52 53
Duration of fecal infectiousness (γ^{fec}, in days) of last waning stage (for PV1;PV2;PV3)<br>- 1 successful IPV<br>- 2 successful IPV<br>- ≥ 3 successful IPV<br>- 1 LPV infection<br>- ≥ 2 LPV infections<br>- IPV and LPV	 26.6;26.4;26.9<br>25.2;25.0;25.5<br>23.8;23.6;24.1<br>14.0;13.9;14.1<br>11.4;11.4;11.6<br>11.4;11.4;11.6	52 53
Duration of oropharyngeal infectiousness (γ^{oro}, in days) of last waning stage (no serotype differences)<br>- 1 successful IPV<br>- 2 successful IPV<br>- ≥ 3 successful IPV<br>- 1 LPV infection<br>- ≥ 2 LPV infections<br>- IPV and LPV	 11.4<br>6.7<br>6.6<br>6.7<br>4.0<br>4.0	52 53
Relative fecal infectiousness (π^{fec}) of last waning stage (no serotype differences)<br>- 1 successful IPV<br>- 2 successful IPV<br>- ≥ 3 successful IPV<br>- 1 LPV infection<br>- ≥ 2 LPV infections<br>- IPV and LPV	 0.95<br>0.9<br>0.85<br>0.5<br>0.3<br>0.3	52 53
Relative oropharyngeal infectiousness (π^{oro}) of last waning stage (no serotype differences)<br>- 1 successful IPV<br>- 2 successful IPV<br>- ≥ 3 successful IPV<br>- 1 LPV infection<br>- ≥ 2 LPV infections<br>- IPV and LPV	 0.43<br>0.25<br>0.13<br>0.5<br>0.3<br>0.3	52 53
Number of reversion stages (*h*)	20	
Shape of reversion function with respect to:<br>- R_0 (z_r)<br>- ln(PIR) (z_p)	 1<br>2.5	

For peer review only - http://bmjopen.bmj.com/site/about/guidelines.xhtml

Average time to reach last reversion stage (ε, in days) (for PV1;PV2;PV3)	620.5; 408; 620.5	30
Paralysis-to-infection ratio for fully susceptible individuals infected with OPV (PIR_0) (for PV1; PV2;PV3)	0.26×10^{-6}; 1.2×10^{-6}; 1.8×10^{-6}	
Paralysis-to-infection ratio for fully susceptible individuals infected with FRPV (PIR_{h-1}) (for PV1; PV2;PV3)	0.005; 0.0005; 0.001	2 14 54
Relative R_0 of OPV vs. FRPV (τ_0) (for PV1; PV2; PV3)	0.37;0.55;0.25	2 52 53
Effective infectious proportion below which we assume 0 force-of-infection (transmission threshold EPI^*)	5/1,000,000	
Relative PIR for maternally immunes compared to fully susceptible individuals ($RPIR_{\mathrm{MI}}$)	0.5	
Ratio of R_0 by serotype in the same setting (PV1:PV2:PV3)	1:0.9:0.75	30
Average incubation period (δ, in days)	10	54 55
Demographics for all situations	Time series 1950-2100	56

Acronyms: CDC = (U.S.) Centers for Disease Control and prevention; cVDPV = circulating vaccine-derived poliovirus; DEB = differential equation-based FRPV = fully-reverted poliovirus; GPLN = Global Polio Laboratory Network; IPV = inactivated poliovirus vaccine; LPV = live poliovirus; OPV = oral poliovirus vaccine; PIR = paralysis-to-infection ratio; PV(1,2,3) = poliovirus (type 1, 2, or 3, respectively); R_0 = basic reproductive number; UN = United Nations; USA = United States of America; VAPP = vaccine-associated paralytic poliomyelitis; VP1 = viral protein 1; WPV(1,2,3) = wild poliovirus (type 1, 2, or 3, respectively)

Notes: [a] Mean estimates obtained from experts and used in the model for the different immunity states, serotypes, and excretion modes vary between 2.85 and 3.37 days

For peer review only

Table A2: Indicative estimates of key variables from Figure 5

Variable	Estimate	Notes and sources
Preparation time needed between certification and OPV cessation	Approximately 1 year	Depends on when setting of the OPV cessation date occurs relative to certification [57]
Planned immunization costs	$1 billion in external GPEI funds per year, plus internal contributions	Most of the $1.1 billion GPEI budget for 2016 was for immunization and coordination of activities;[58] Countries may internally contribute at a similar rate as the external contributions;[59] The current GPEI budget projects a decrease from 2018 forward, which would imply some offset of costs for maintenance of activities, or alternatively the activities previously supported by external contributions may end, which would imply declines in programmatic activities and quality
OPV-related polio cases	Hundreds per year	Vaccine-associated paralytic polio cases,[60] which depends on timing of IPV doses,[61] and presumably local cVDPV outbreaks[62]
Surveillance costs	Around $100 million per year	The 2016 GPEI budget included $67 million in external support for surveillance and laboratories,[58] with additional significant internal contributions by countries [59 63]
Probability of OPV restart due to WPV reemergence	Unknown	Prior studies estimated an approximately 5% chance of an OPV restart due primarily to OPV-associated risks, although the actual implementation of risk management policies was not as good as suggested by these models.[59 64]
Immunization costs associated with an OPV restart	$ billions (hundreds of millions per year)	An OPV restart would involve reintroduction of OPV vaccination in most countries in perpetuity, with supplemental immunization activities needed in countries with insufficient routine immunization coverage.[59] Significant uncertainty exists about what an OPV restart would look like in practice.
Expected cases due to an OPV restart	Up to thousands per year	Reintroduction of OPV in most countries would result in hundreds of vaccine-associated paralytic polio cases per year and could result in continued cVDPV outbreaks in countries with insufficient routine immunization coverage that do no conduct regular preventive supplemental immunization activities.[59 64]

For peer review only - http://bmjopen.bmj.com/site/about/guidelines.xhtml

Figure A1: Schematic of the DEB model structure, adopted from Duintjer Tebbens et al. (2013)[2, p. 706]

(a) Immunity states and flows between them due to epidemiological events

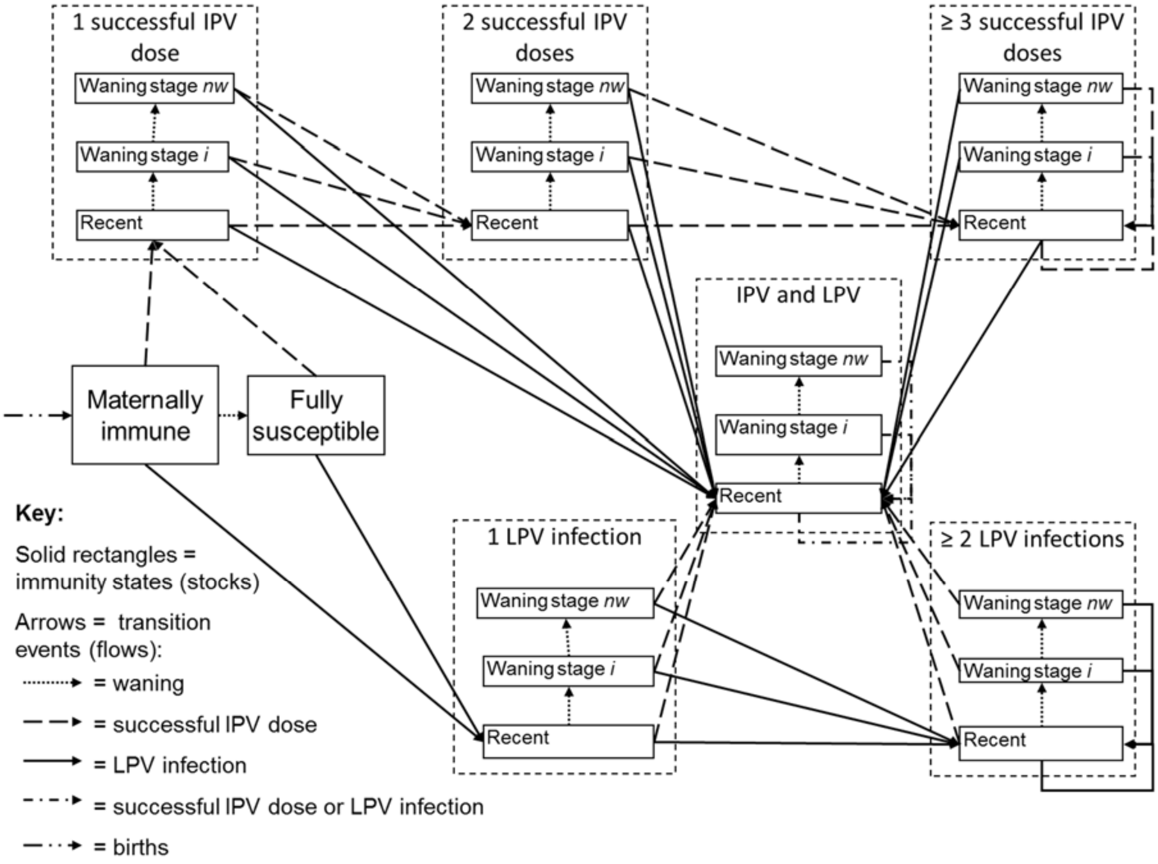

For peer review only - http://bmjopen.bmj.com/site/about/guidelines.xhtml

(b) Progression through infection and reversion stages

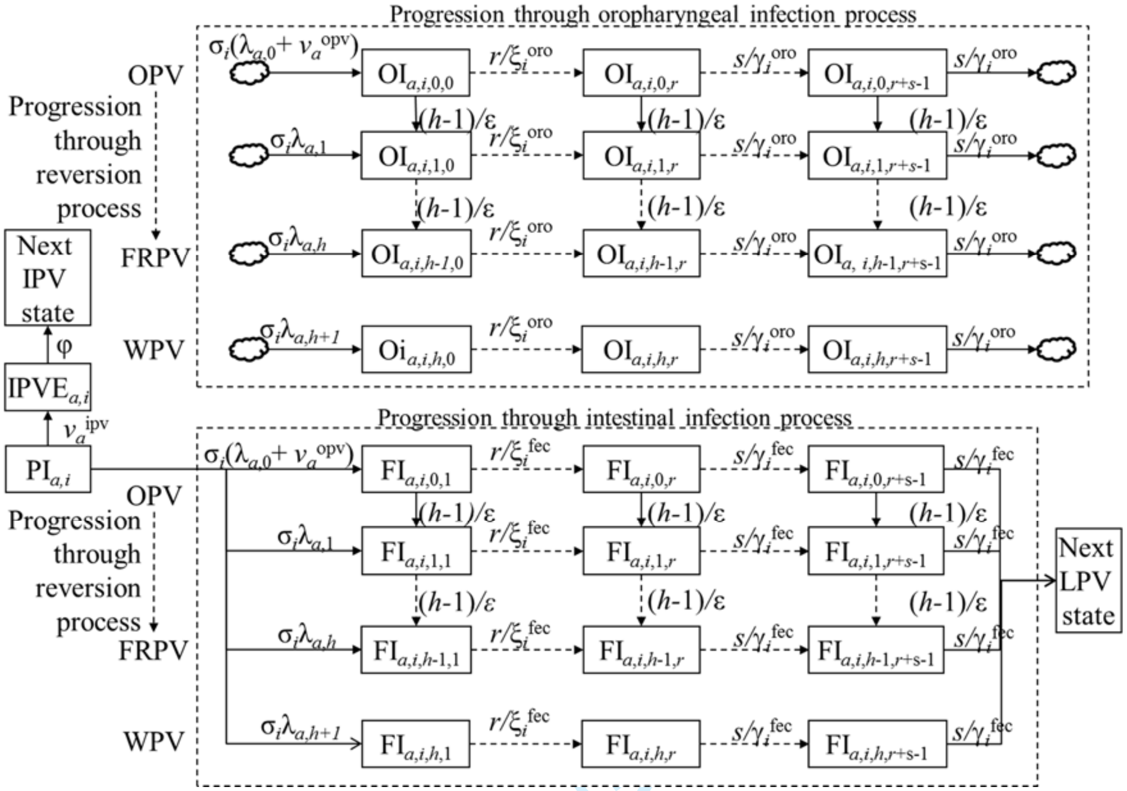

"Acronyms: FRPV = fully-reverted poliovirus; IPV = inactivated poliovirus vaccine; OPV = oral poliovirus vaccine; WPV = wild poliovirus**; Symbols:** $PI_{a,i}$ = partially infectible in age group *a* and immunity state *I*; $IPVE_{a,i}$ = IPV-exposed individual from immunity state *i* and age group *a*; $FI_{a,i,j,k}$ ($OI_{a,i,j,k}$) = individual in age group a from immunity state *i*, infected with virus strain *j* and in fecal (oropharyngeal) infection stage *k*; $\lambda_{a,j}$ = force-of–infection to age group *a* for virus strain *j;* ν_a^{ipv} (ν_a^{opv}) = force-of-IPV(OPV)-vaccination to age group *a* as a result of routine and supplementary immunization; σ_i = relative susceptibility for immunity state *i*; ξ_i^{fec} (ξ_i^{oro}) = average duration of the fecal (oropharyngeal) latent period for immunity state *i*; γ_i^{fec} ($\gamma_i^{oro)}$ = average duration of the fecal (oropharyngeal) infectious period for immunity state *i*; φ = IPV immunity delay; *h* = number of reversion stages; *r* = number of latent stages; *s* = number of infectious stages" [2, p. 706]

For peer review only - http://bmjopen.bmj.com/site/about/guidelines.xhtml

Figure A2: Summary results from the model calibration process, adapted from Duintjer Tebbens et al. (2013)[2]

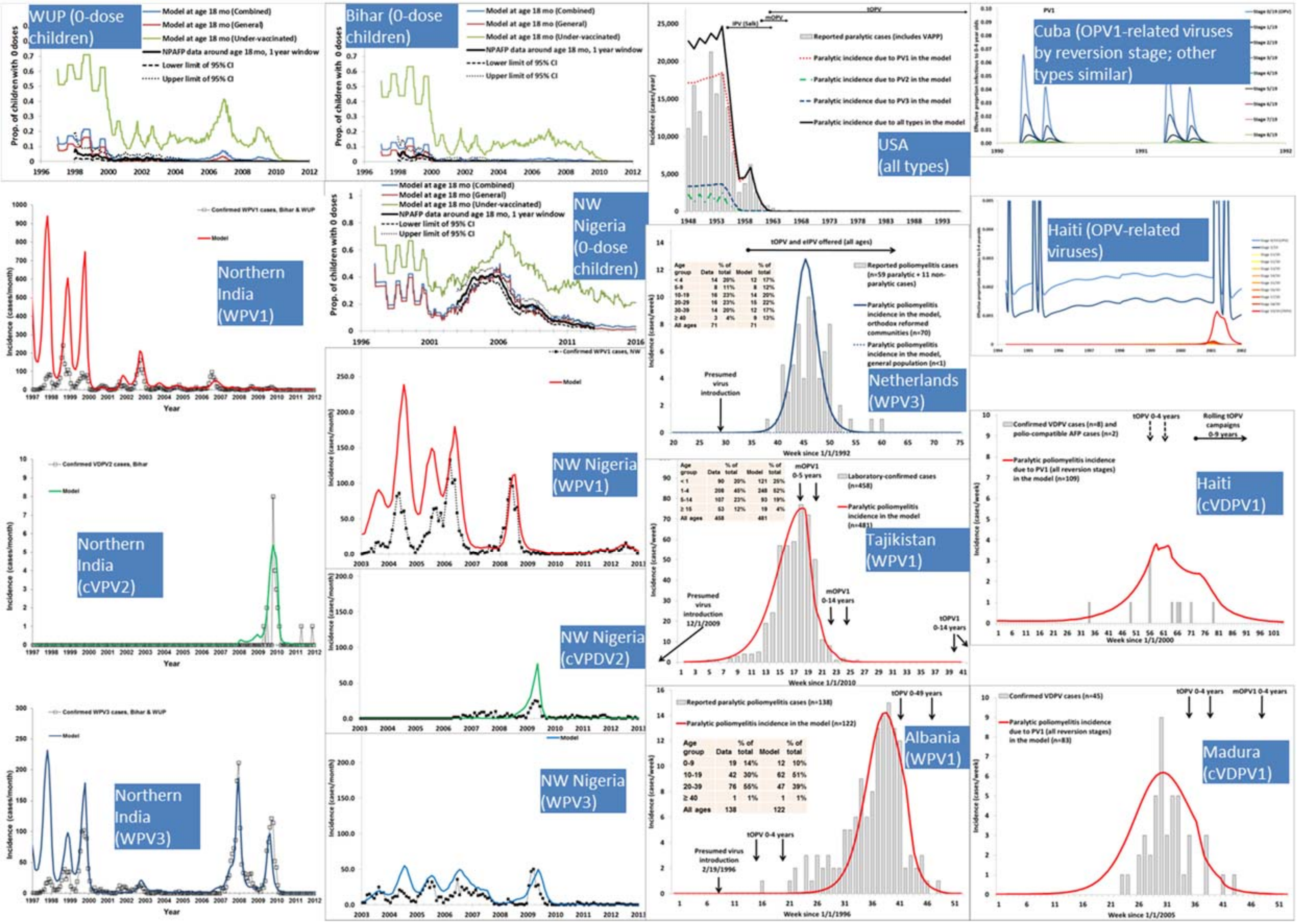

Figure A3: Differential-equation based model results for base case model inputs and varied coverage (left column), varied degree of isolation with coverage 0.82 (middle column), and varied relative size with coverage of 0.82 (right column). The y-axis scales linearly with total population size (all figures assume a total population size of 1 million).

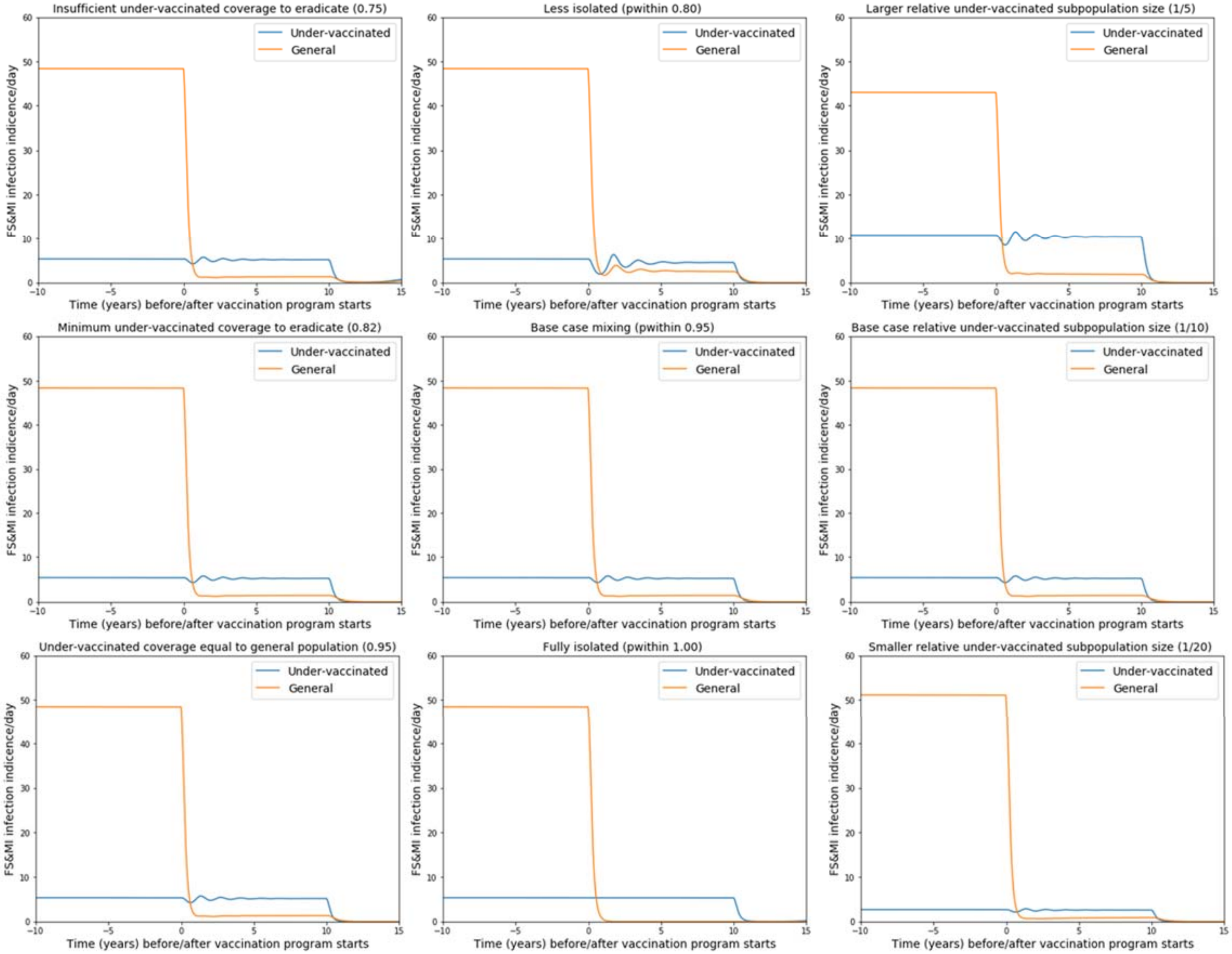

BMJ Open

Global certification of wild poliovirus eradication: Insights from modeling hard-to-reach subpopulations and confidence about the absence of transmission

Journal:	*BMJ Open*
Manuscript ID	bmjopen-2018-023938.R1
Article Type:	Research
Date Submitted by the Author:	29-Oct-2018
Complete List of Authors:	Duintjer Tebbens, Radboud; Kid Risk, Inc.<br>Kalkowska, Dominika; Kid Risk, Inc.<br>Thompson, KM; Kid Risk, Inc.,
**Primary Subject Heading**:	Infectious diseases
Secondary Subject Heading:	Health policy
Keywords:	polio, eradication, certification, modeling

SCHOLARONE™
Manuscripts

Global certification of wild poliovirus eradication: Insights from modeling hard-to-reach subpopulations and confidence about the absence of transmission

Radboud J. Duintjer Tebbens,[1] Dominika A. Kalkowska,[1] Kimberly M. Thompson[1]

1. Kid Risk, Inc., Columbus, OH, USA

Running title: **Poliovirus certification confidence**

Correspondence to: Kimberly M. Thompson, Kid Risk, Inc., 605 N High St, #253, Columbus, OH 43215, USA, Email: kimt@kidrisk.org

Abstract:

Objective: To explore the extent to which under-vaccinated subpopulations may influence the confidence about no circulation of wild poliovirus (WPV) after the last detected case.

Design and participants: We used a hypothetical model to examine the extent to which the existence of an under-vaccinated subpopulation influences the confidence about no WPV circulation after the last detected case as a function of different characteristics of the subpopulation (e.g., size, extent of isolation). We also used the hypothetical population model to inform the bounds on the maximum possible time required to reach high confidence about no circulation in a completely-isolated and unvaccinated subpopulation starting either at the endemic equilibrium or with a single infection in an entirely susceptible population.

Results: It may take over three years to reach 95% confidence about no circulation for this hypothetical population despite high surveillance sensitivity and high vaccination coverage in the surrounding general population if: (1) ability to detect cases in the under-vaccinated subpopulation remains exceedingly small, (2) the under-vaccinated subpopulation remains small and highly isolated from the general population, and (3) the coverage in the under-vaccinated subpopulation remains very close to the minimum needed to eradicate. Fully-isolated hypothetical populations of 4,000 people or less cannot sustain endemic transmission for more than 5 years, with at least 20,000 people required for a 50% chance of at least 5 years of sustained transmission in a population without seasonality that starts at the endemic equilibrium.

For peer review only

Notably, however, the population size required for persistent transmission increases significantly for realistic populations that include some vaccination and seasonality and/or that do not begin at the endemic equilibrium.

Conclusions: Significant trade-offs remain inherent in global polio certification decisions, which underscore the need for making and valuing investments to maximize population immunity and surveillance quality in all remaining possible WPV reservoirs.

Strengths and limitations of this study:

- Demonstrates the somewhat limited but important role of under-vaccinated subpopulations in the time required to achieve high confidence about no WPV transmission after the last reported case.
- Highlights competing trends as time increases such that for smaller population sizes continued transmission becomes exceedingly unlikely, while for larger population sizes undetected circulation becomes less likely due to the higher frequency of cases and greater chances of detection.
- Results underscore the importance of continued investments to maximize population immunity and surveillance quality.
- Analyses remain limited by model assumptions, but in abstract provide insights relevant to likely last poliovirus reservoirs.

Keywords: polio, eradication, certification, modeling

For peer review only - http://bmjopen.bmj.com/site/about/guidelines.xhtml

Background

Achieving the 1988 World Health Assembly polio eradication goal of ending all cases of poliomyelitis [1] requires a successful transition from the interruption of the current low level of wild poliovirus (WPV) transmission through coordinated cessation of all use of live attenuated oral poliovirus vaccine (OPV) to effective long-term risk management. The Global Polio Laboratory Network supports the Global Polio Eradication Initiative (GPEI) by testing stool samples from acute flaccid paralysis (AFP) cases and sewage samples for polioviruses. As the GPEI approaches success, the transition to the polio endgame has begun. The endgame involves significant complexity, because all countries must achieve and maintain sufficient population immunity [2-4] to stop and prevent the transmission of three separate poliovirus serotypes (i.e., 1, 2, and 3) and globally coordinate cessation of each OPV serotype.[5-7] In September 2015, the Global Certification Commission declared successful eradication of serotype 2 WPV (WPV2),[8] which represented a prerequisite to the globally-coordinated cessation of all serotype 2-containing OPV use. Global cessation of serotype 2-containing OPV occurred in late April and early May 2016, during which time over 150 countries stopped using trivalent OPV (tOPV, which contains all three serotypes) and switched to bivalent OPV (bOPV, which contains only serotypes 1 and 3 OPV).[9]

The Global Polio Laboratory Network reported the lowest number of annual paralytic serotype 1 WPV (WPV1) cases in 2017,[10] and no serotype 3 WPV (WPV3) cases since November 2012.[11] Successful WPV eradication requires stopping all transmission, which manifests as an absence of detected WPVs despite actively looking. With increasing time of not seeing cases (while actively looking), confidence increases about WPV die-out. However, the absence of evidence is not evidence of absence. Extended silent transmission can occur, because most poliovirus infections do not lead to symptoms and surveillance gaps can exist. For example, a WPV3 resurfaced in Sudan/Chad in 2004 after no reported cases during 1997-2003 [12] and a WPV1 resurfaced in Borno, Nigeria in 2016 after nearly 3 years with no reported cases [13]. The average paralysis-to-infection ratio (PIR), defined as the fraction of infections in fully susceptible individuals that leads to paralytic poliomyelitis (polio) symptoms, equals approximately 1/200, 1/2000, and 1/1000, for serotype 1, 2, and 3 WPV, respectively.[14] The last reported naturally-

For peer review only - http://bmjopen.bmj.com/site/about/guidelines.xhtml

occurring WPV2 case occurred in India in 1999,[15] and since then, only two episodes of WPV2 infections occurred that traced back to laboratory strains.[16 17] Despite the possibility of silent circulation, the absence of any naturally-occurring WPV2 cases for over 15 years (and in many countries for many decades) led to very high confidence about the die-out of WPV2 transmission.

Multiple prior mathematical modeling studies explored the probability of undetected circulation of WPVs in the absence of reported cases or other poliovirus detections. Polio eradication efforts in the Americas, which reported the last indigenous WPV case of any serotype in Peru in 1991,[18] motivated the first analysis and discussion of certification requirements. A statistical analysis of Pan American Health Organization epidemiological data reported less than a 5% chance of undetected indigenous WPV circulation after 4 years since the last reported confirmed case.[19] A simple, stochastic model of poliovirus transmission and die-out characterized the probability of undetected poliovirus circulation in a hypothetical, homogeneously mixed population of 200,000 people in a relatively low-income country, and estimated that not observing a case for 3 years provided 95% confidence about local extinction of WPV infections.[20] This seminal paper provided the foundation for appropriate characterization of the probability of undetected circulation as a function of the time since the last detected case.[20] Related modeling also explored theoretical thresholds to stop transmission [21] and estimated a minimum population size for persistent transmission of 50,000-100,000 in developing countries and over 200,000 in developed countries required to achieve at least 95% probability of poliovirus persistence for 5 years or more in the absence of vaccination.[22] These studies supported the 2004-8 GPEI Strategic Plan requirement of at least 3 years of no polio cases detected by AFP surveillance for certification,[23] which remains the current minimum requirement.[24] A 2012 study [25] relaxed some of the assumptions of the prior theoretical model [20] and highlighted that the probability of undetected circulation varied for different poliovirus serotypes, places, and conditions, which suggested the need to focus on appropriate characterization of conditions in the last likely WPV reservoirs.[25] A 2015 study [26] also used the prior model [20] to show that in the context of an instantaneous introduction of vaccination, the time of the last case relative to vaccine introduction further informs the confidence about the absence of circulation.

For peer review only

Subsequent analyses focused on modeling the conditions in specific and more realistic populations. A 2015 study [27] used a previously-developed poliovirus dynamic transmission model [2] applied to: recently-endemic transmission in two states in northern India,[28] endemic transmission in northwest Nigeria,[29] a 2010 outbreak in Tajikistan,[30] and transmission following a 2013 WPV1 introduction into Israel detected by environmental surveillance.[31] The study characterized the confidence about no undetected poliovirus circulation by serotype as a function of time without reported polio cases or environmental detections considering realistic assumptions for surveillance, immunization, and other national inputs.[27] The results suggested that time periods of 0.5 to 3 years without detected polio cases provided 95% confidence about the interruption of transmission in the context of perfect AFP surveillance depending on situation-specific characteristics (e.g., the overall population immunity, endemic versus outbreak conditions, and virus serotype).[27] This model also suggested longer times required for less-than-perfect AFP surveillance and potentially shorter times using highly-sensitive environmental surveillance based on the experience in Israel.[27] A recent statistical analysis of the 2013 WPV1 outbreak in Israel demonstrated a rapid increase in confidence about no undetected local transmission following outbreak response immunization after repeated negative environmental surveillance samples in a city.[32] A non-dynamic, statistical model [33] estimated a shorter time (compared to [27]) of 14 months required to reach high confidence about no undetected circulation. For its most conservative assumptions about surveillance and force-of-infection, the study estimated a probability of 93% of a WPV-free Africa in the absence of any new WPV cases reported by the end of 2015,[33] shortly before the WPV reemerged.[13] Contrasting with all other modeling studies, a recent study [34] suggested a relatively high probability of undetected circulation after more than 3 years without any polio cases in small populations, although a correction to that analysis emphasized the unrealistic nature of one of the assumptions.[35] Remarkably, the analysis reported that closed populations of 10,000 people or fewer could support many years of transmission in the absence of vaccination, and experience gaps between polio cases of over 5 years.[34] A reanalysis of this hypothetical model identified issues with the analysis and its framing, and reported results consistent with the prior literature after correcting for some errors.[36]

For peer review only - http://bmjopen.bmj.com/site/about/guidelines.xhtml

Although the modeling results demonstrated the critical importance of sustaining high population immunity through immunization programs and high-quality surveillance to obtain high confidence about no undetected circulation, the current GPEI strategic plan only covers 2013-2018,[6] which leads to uncertainty about the ability to sustain high program performance after 2018. As of mid-2018, questions continue to arise about when the GPEI will cease to exist and what resources will be available to support the polio endgame, including the certification of eradication of WPV1 and WPV3 with high confidence. The GPEI partners already began transition planning, and this process already led to some downsizing of national poliovirus programs, including the reduction of some AFP surveillance activities.[37] Thus, while the prior modeling assumed strong GPEI and national polio program performance up through the end of the polio endgame, this assumption now appears optimistic, and further analyses that explore the impact of lower quality surveillance may prove useful in the context of global certification decisions for WPV1 and WPV3 eradication. Further motivation for developing models to support certification decisions comes from the re-appearance of WPV1 in security-compromised areas in Borno, Nigeria after apparent interruption, which raised questions about the ability of poliovirus circulation without detection in communities not (or poorly) accessed by immunization and surveillance efforts within larger populations with high immunity and good surveillance.

This study aims to support future decisions about WPV certification by: (1) informing confidence about the absence of circulation by modeling the role of hard-to-reach populations, (2) examining the minimum population size required to sustain poliovirus transmission, and (3) developing a conceptual framework to provide some structure for future certification decisions,.

Methods

To inform confidence about the absence of circulation by modeling the role of hard-to-reach populations, we explored the impact of key assumptions using an existing model of a hypothetical population comprised of a well-vaccinated general population and an under-vaccinated subpopulation.[38] Table 1 lists the model inputs used to characterize this hypothetical population and explore the role of key assumptions (see appendix for model details). To explore

For peer review only - http://bmjopen.bmj.com/site/about/guidelines.xhtml

different population characteristics, we varied the total population size, the size of the under-vaccinated subpopulation, and the degree of mixing between the under-vaccinated and general population around a base case indicated by the bold values in Table 1. In addition, for each variation around the base case, we simultaneously varied the routine immunization coverage and detection probability per polio case in the under-vaccinated subpopulation. We interpret the total hypothetical population as one epidemiological block (e.g., a country) and therefore compute the confidence about no circulation based on all detections that occur in the general population and under-vaccinated subpopulation combined. However, we fix the detection probability in the general population at 95% to characterize high-quality national surveillance while considering lower detection probabilities only in the under-vaccinated subpopulation (Table 1).[38] To estimate the confidence about no circulation in this conceptual model, we use a simplified version (see appendix) of the stochastic approach developed by Eichner and Dietz (1996) [20] and adopted by others.[25-27] We define the probability of undetected circulation after a given period of *t* months without a detection as the number of times in multiple stochastic simulations that *t* months went by without a detection despite continued circulation, divided by the total number of times that *t* months went by without a detection (i.e., with or without continued circulation). Intuitively, the fraction of all time periods of *t* months without a detection but with transmission still ongoing should decrease as *t* increases, corresponding to an increasing probability of no circulation. Confidence about no circulation equals one minus the probability of undetected circulation. To visualize the impact of varying the model inputs, we focus on the time without a detection until the confidence about no circulation first exceeds 95% (CNC95%).

We revisit the question of silent transmission in small populations [22 34 36] using the hypothetical population model [38] in an attempt to inform the bounds on the maximum possible CNC95%. To do so, we ignore the general population and effectively assume a completely-isolated and unvaccinated subpopulation and otherwise adopt the hypothetical population assumptions from Table 1. We transform the DEB model to a stochastic form using the Gillespie algorithm,[39] as described elsewhere, [27] and start either at the endemic equilibrium [34] or with a single infection in an entirely susceptible population. Instead of modeling die-out using the transmission threshold,[2 27] we allow transmission to continue until the infection prevalence becomes 0. This complements the existing work [22 34 36] by providing a comparison to the same situation with a

For peer review only - http://bmjopen.bmj.com/site/about/guidelines.xhtml

more comprehensive model for poliovirus transmission,[2] adding consideration of the impact of the initial conditions, and adding the impact on confidence about no circulation.

Finally, recognizing the complexity and inter-related nature of certification decisions, we developed an influence diagram to relate certification timing decisions to outcomes. The diagram provides a conceptual framework to support certification decisions and formulate decisions about the timing of certification as an optimization problem. The diagram uses conventions from causal loop diagrams [40] and specifies the directionality of relationships between variables using unidirectional arrows. The polarity or sign at the arrow head indicates whether increasing the variable at the base of the arrow increases (+) or decreases (-) the variable that the arrow points to with all else being equal.

Patient and Public Involvement

This survey did not involve patients or public opportunities for engagement.

Results

Figure 1 illustrates how the confidence about no circulation increases with time after the last detection as a function of the surveillance quality in the under-vaccinated subpopulation (i.e., the detection probability). Clearly, higher confidence implies the need to wait longer after the last detected case, and lower detection probabilities further increase the time required to reach a certain level of confidence (e.g., the 95% line). Figure 1 shows a relatively modest effect of the detection probability in the under-vaccinated subpopulation for this hypothetical model due to continued occurrence of cases in the general population for the assumed degree of mixing (see appendix).

Figure 2 shows the CNC95% values as a function of coverage and detection probability for the under-vaccinated subpopulation. The figure shows longer times required to reach CNC95% values with increasingly more isolated under-vaccinated subpopulations (left column, top to bottom), with decreasing relative sizes of the under-vaccinated subpopulation (middle column,

For peer review only

For peer review only - http://bmjopen.bmj.com/site/about/guidelines.xhtml

top to bottom), and decreasing absolute sizes of a fully-isolated under-vaccinated subpopulation (right column, top to bottom, note increased y-axis ranges). The panels in Figure 2 omit curves for coverage values that do not result in eradication, because they do not allow for calculation of any confidence about eradication. The panels also omit the data point for 0 detection probability in the event of a fully-isolated under-vaccinated subpopulation, because that would imply no ability to detect the virus. Consistent with previous findings,[27] all panels in Figure 2 show higher CNC95% values with higher coverage in the under-vaccinated subpopulation. In each panel, the lowest shown coverage value may result in the longest period of undetected circulation before interruption and therefore result in the longest time to achieve high confidence about no circulation.

Looking more closely at the differences between the columns, the left column of Figure 2 shows a very strong influence of the degree of isolation on the CNC95%. With little isolation and no surveillance in the under-vaccinated subpopulation, the general population with high surveillance quality can still detect transmission because of relatively frequent spillover of polio cases (see appendix). Thus, the results do not depend much on the detection probability in the under-vaccinated subpopulation for p_{within}=0.8. In contrast, for a fully isolated under-vaccinated subpopulation (p_{within}=1), the detection probability in this population becomes a more important driver of the CNC95% than the coverage (i.e., for detection probability of 0.1 or very poor surveillance and all other inputs at the base case, the CNC95% equals almost 6 years regardless of coverage). The middle column of Figure 2 shows CNC95% values of approximately 5 years with no surveillance in a relatively small under-vaccinated subpopulation. Although the relative size of the under-vaccinated subpopulation affects the mixing dynamics and incidence of cases in both populations, much of the observed effect comes from the implied change in the absolute size of the under-vaccinated subpopulation, which directly affects the typical time between cases. As shown in the right column of Figure 2, changing the absolute size of the under-vaccinated subpopulation in the event of full isolation from the general population and a detection probability of 0.1 dramatically affects the CNC95%, which ranges from slightly over 2 years for 500,000 people to approximately 9 years for 50,000 people (i.e., a 4-fold increase in CNC95% for a 10-fold increase in population size).

For peer review only - http://bmjopen.bmj.com/site/about/guidelines.xhtml

Considering the relatively high CNC95% observed for small, isolated populations in Figure 2, Figure 3a uses a stochastic model to show the distribution of the duration of circulation in a single population not reached by vaccination at all. Figure 3a shows the results as a function of population size for a model initialized at the endemic equilibrium. For very small population sizes (e.g., hundreds), not surprisingly poliovirus infections typically die-out within a year, with a maximum duration of circulation of one year and 4 months for a closed population of 1,000 people (based on 10,000 iterations). The maximum duration of circulation increases rapidly for larger populations. For a population of 5,000 people, circulation continues for 3 or more years in 50 of 10,000 (0.5%) iterations. With population sizes of 10,000, 20,000, 30,000, 40,000 and 50,000, circulation continues for at least 10 years for 3%, 34%, 63%, 79%, and 88% of iterations, respectively.

Figure 3b shows the same analysis as Figure 3a except that it changes the initial conditions by assuming a population with no prior exposure to any polioviruses. In this context, a single introduction rapidly burns through the entire susceptible population and quickly exhausts susceptible individuals, leading to die-out and a maximum duration of circulation of less than 2 years for all population sizes considered in Figure 3b. Together, Figures 3a-b encompass the bounds on the possible duration of circulation for different initial conditions. In reality, small, completely isolated populations are unlikely to remain at the endemic equilibrium because of random fluctuations in the incidence, seasonality, and die-out, and no completely naïve populations likely exist. In a separate analysis using the same model, we verified that the addition of seasonality decreases the typical duration of circulation and increases the probability of eradication within 5 years. For example, for a population size of 20,000 people, the probability of eradication within 5 years increased from approximately 64% without seasonality to 78%-92% with a seasonal amplitude of 10% (applied to the basic reproduction number of 10), depending on the timing of the seasonal peak.

While Figure 3 implies that increasing the population size results in an increasing probability of persistent circulation (i.e., a greater probability of sustained undetected transmission), Figure 2 implies that increasing population size decreases the typical time interval between cases (i.e., lower probabilities of sustained undetected circulation). Figure 4 shows the net effect of these

For peer review only - http://bmjopen.bmj.com/site/about/guidelines.xhtml

two opposing trends and suggests that an optimal population size exists around 20,000 people. For smaller population sizes, continued transmission becomes exceedingly unlikely (Figure 3), while for larger population sizes, undetected circulation becomes less likely due to the higher frequency of cases. This non-linear behavior suggests a maximum CNC95% of approximately 2.5 years for a detection probability of 1, although the maximum increases to up to 9 years for a very low detection probability of 0.1 and a population size of 20,000 to 30,000 people.

Figure 5 shows how the desired confidence about no circulation may influence certification timing and key health economic outcomes (see appendix for details). Earlier certification and OPV cessation may increase the risk of undetected circulation after OPV cessation (and therefore the possibility of needing to restart OPV use) but may decrease the costs until OPV cessation (and therefore the overall global costs for planned polio immunization). Therefore, the fundamental optimization problem consists of finding the desired confidence about no WPV circulation at OPV cessation that minimizes the resulting total financial and societal costs. Figure 5 also shows that the costs and risks both depend on the GPEI budget until and after OPV cessation, with a lower budget saving costs in the short term but increasing the time of OPV cessation at a given confidence level and the risks of OPV restarts, which may ultimately result in greater overall costs. Optimization of the desired confidence about no WPV circulation depends critically on how the confidence about no circulation increases with time after the last detected event from the surveillance system.

Discussion

Hard-to-reach subpopulations may play a key role in deliberations about WPV circulation and decisions about WPV certification. The timing of WPV certification and subsequent OPV cessation involves high stakes and largely depends on the desired confidence about the absence of circulation. Surveillance quality emerges as a key factor that affects both the confidence about the absence of circulation and the ability to detect and control any outbreaks after OPV cessation. However, national surveillance indicators may not suffice to measure the overall surveillance system quality because gaps in surveillance at the level of tens of thousands of people may influence confidence. Our modeling suggests that high quality surveillance suffices

For peer review only - http://bmjopen.bmj.com/site/about/guidelines.xhtml

to detect transmission in the context of a relatively well-mixed under-vaccinated subpopulation (e.g., in Pakistan and Afghanistan),[41] while local gaps may miss transmission for several years in the context of highly-isolated under-vaccinated subpopulations. With respect to global certification of WPV eradication, this implies a need to address any such gaps in isolated populations that experienced WPV transmission during the last decade. The recent experience in Borno and previously in Chad and Sudan demonstrated the ability of WPVs to circulate undetected for many years in sub-populations missed by both surveillance and immunization efforts.[12 13] However, one of the main contributions of this work is that is shows that very small, isolated subpopulations cannot sustain transmission indigenously, while in the context of even very limited surveillance, persistent undetected transmission becomes increasingly unlikely for increasing population sizes. To our knowledge, the existence of a worst-case population size for undetected circulation has not yet been demonstrated for polioviruses. Our analysis confirms that with high-quality surveillance, 3 years without a detected WPV case suffices to attain high confidence about no circulation for serotype 1, even considering possible persistence in very small population sizes.

Explicit consideration of the decision to certify WPV eradication (Figure 5) suggests that if we remain confident that we can prevent the need to restart OPV due to uncontrolled outbreaks resulting from a possible WPV reemergence, then we should accept a lower confidence about the absence of circulation to certify sooner, because the costs of delaying OPV cessation would outweigh the risk of premature certification. Earlier OPV cessation particularly represents the best option if diminishing GPEI financial and/or global OPV supply resources limit our ability to maintain population immunity and/or respond effectively to post-cessation outbreaks. However, this choice depends on a willingness to accept the reputational risk of finding out that WPV still circulates despite its certification. With WPV3 not detected anywhere since 2012 [11] and in many places for decades, the confidence about no WPV3 circulation continues to grow. Although confidence about no circulation increases more slowly for WPV3 than WPV1 due to the lower PIR,[25 27] assuming 1-2 years to prepare for coordinated global OPV cessation, starting the process of removing serotype 3 OPV now would imply at least 7 years of no detection since the last WPV3 case and synchronized cessation of serotype 3 OPV use (i.e., 2012 to 2019-2020). The transition of GPEI resources already occurring leads to expected decreases in population

For peer review only - http://bmjopen.bmj.com/site/about/guidelines.xhtml

immunity for serotype 3 in some areas. Combined with on-going serotype 3 vaccine-associated paralytic poliomyelitis, this should motivate careful consideration of the costs, benefits, risks, and logistical challenges of globally certifying WPV3 eradication and synchronizing serotype 3 OPV cessation before completing WPV1 eradication and serotype 1 OPV cessation, which now appears at least 4 years away.

Our results related to minimum population sizes appear consistent with a prior study [22] that found an average of approximately 5 years of circulation for a population of 20,000 people in a high-R_0 setting and an exponential increase in the average duration of circulation with increasing population size. The prior study also reported a higher probability of virus persistence as the degree of mixing between subpopulations increases.[22] Our study suggests that more mixing between subpopulations may not lead to a higher probability of undetected circulation because surveillance can more easily detect persistent viruses for higher degrees of mixing. Using a more realistic model than another prior analysis,[36] we similarly do not find a high probability of persistent transmission for populations of 10,000 people or less.

Like all models, our model makes simplifying assumptions that affect its behavior.[2] Specifically, we characterized a stylized, hypothetical population to systematically explore key assumptions, used a simplified semi-stochastic approach to compute CNC95% that does not fully account for all stochastic variability, and deterministically characterized die-out. However, for the analysis of small population sizes that depend most on stochastic variability, we accounted for stochastic variability and die-out at the individual level.

While this study highlights the importance of ensuring high surveillance quality in all subpopulations, it also reiterates the role of immunization in accelerating confidence about no circulation after the last detection.[27] Achieving and maintaining high population immunity to transmission represents a mission critical component of the GPEI.[4] Populations with immunity near the threshold experience increased risk of prolonged undetected transmission. Failing to invest relatively small amounts of resources to maintain high population immunity can lead to much more costly outbreaks, as occurred for example in Tajikistan.[3] Thus, if ensuring high-quality surveillance in all subpopulations remains an elusive goal, then achieving better coverage

For peer review only - http://bmjopen.bmj.com/site/about/guidelines.xhtml

in those subpopulations would still result in higher confidence about no circulation. In contrast, high quality surveillance in the context of poor immunization still leaves the population and the world at risk.

Poliovirus environmental surveillance can detect polioviruses even in the absence of symptomatic polio cases [42 43] and offers the potential to fill some local gaps in symptomatic poliovirus surveillance. For example, the extensive environmental surveillance system in Israel effectively detected transmission of circulating WPV1 in the absence of any cases and despite very high coverage with inactivated poliovirus vaccine (IPV).[31 44] However, despite the potential for high sensitivity of environmental surveillance to detect infected individuals excreting into the catchment area, its sensitivity remains zero outside of the catchment area and depends on sampling frequency (e.g., one sample every year provides little increase in confidence over AFP alone).[45] Environmental surveillance system designs generally depend on access to a centralized sewage network,[43] which hard-to-reach subpopulations (i.e., those most likely to sustain undetected poliovirus transmission) may not possess. Further research should help to explore the ability of environmental surveillance to increase confidence about no circulation in specific areas, and the value of the information obtained from environmental surveillance relative to its costs requires evaluation.

Overall, IPV plays a relatively limited role with respect to the CNC. While IPV protects otherwise susceptible individuals from paralysis if they become subsequently infected with a live poliovirus and may reduce the participation of these individuals in transmission to some degree, the decreased frequency of paralysis may delay the detection of any circulating live poliovirus in countries with surveillance systems that rely on AFP surveillance (i.e., the detection of cases). Overall, immunization with IPV helps to maintain population immunity to transmission somewhat, but given births of immunologically naïve, deaths of immune individuals, waning immunity, and the absence of circulating live polioviruses, population immunity to transmission declines following WPV eradication and homotypic OPV cessation, even with very high IPV coverage.[46] The extent of transmission possible following reintroduction of a live poliovirus into a country with high IPV coverage will depend on the relative contributions of fecal-oral and oropharyngeal routes to overall transmission.[4] In countries dominated by fecal-oral

For peer review only - http://bmjopen.bmj.com/site/about/guidelines.xhtml

transmission, the use of IPV will not prevent or stop transmission, and reintroduced live polioviruses that restart transmission may lead to the need to restart the use of OPV.[47]

List of abbreviations: AFP, acute flaccid paralysis; CNC95%, Time until the confidence about no circulation reaches 95%; cVDPV, circulating VDPV DEB, differential-equation based; GPEI, Global Polio Eradication Initiative; IPV, inactivated poliovirus vaccine; OPV, oral poliovirus vaccine; PIR, paralysis-to-infection ratio; VDPV, vaccine-derived poliovirus; WPV(1,2,3), wild poliovirus (of serotype 1, 2, 3, respectively)

DECLARATIONS

Authors' contributions

All authors (RDT, DAK, KMT) contributed to the study design, model development, interpretation of results, manuscript writing, and revisions. The first and second authors (RDT, DAK) performed the modeling and analyses, and the last author (KMT) secured the funding for the study.

Ethics approval and consent to participate

Not applicable

Consent to publish

Not applicable

Competing interests

None

Funding

This work was funded by the Bill and Melinda Gates Foundation [OPP1129391].

Acknowledgments

For peer review only

We thank the Bill and Melinda Gates Foundation for supporting the completion of this work [OPP1129391].

Data sharing statement

Technical appendix available on request from the authors.

References

1. World Health Assembly. Global eradication of poliomyelitis by the year 2000 (resolution 41.28). Geneva: World Health Organization, 1988.
2. Duintjer Tebbens RJ, Pallansch MA, Kalkowska DA, et al. Characterizing poliovirus transmission and evolution: Insights from modeling experiences with wild and vaccine-related polioviruses. *Risk Analysis* 2013;23(4):703-49.
3. Thompson KM, Pallansch MA, Duintjer Tebbens RJ, et al. Modeling population immunity to support efforts to end the transmission of live polioviruses. *Risk Analysis* 2013;33(4):647-63.
4. Thompson KM, Kalkowska DA, Duintjer Tebbens RJ. Managing population immunity to reduce or eliminate the risks of circulation following the importation of live polioviruses *Vaccine* 2015;33(3):1568-77.
5. Thompson KM, Duintjer Tebbens RJ. The case for cooperation in managing and maintaining the end of poliomyelitis: Stockpile needs and coordinated OPV cessation. *The Medscape Journal of Medicine* 2008;10(8):190.
6. World Health Organization. Global Polio Eradication Initiative: Polio Eradication and Endgame Strategic Plan (2013-2018). Geneva, 2013.
7. World Health Assembly. Poliomyelitis: mechanism for management of potential risks to eradication (resolution 61.1). Geneva: World Health Organization, 2008.
8. Global Polio Eradication Initiative. Global eradication of wild poliovirus type 2 declared 2015 [updated September 20, 2015. Available from: http://www.polioeradication.org/mediaroom/newsstories/Global-eradication-of-wild-poliovirus-type-2-declared/tabid/526/news/1289/Default.aspx accessed November 30 2015.
9. Hampton LM, Farrell M, Ramirez-Gonzalez A, et al. Cessation of trivalent oral poliovirus vaccine and introduction of inactivated poliovirus vaccine - Worldwide, 2016. *Morbidity and Mortality Weekly Report* 2016;65(35):934-38. doi: 10.15585/mmwr.mm6535a3
10. World Health Organization. Polio this week as of 3 January 2018 2018 [Available from: http://polioeradication.org/polio-today/polio-now/this-week/ accessed January 10 2018.
11. Kew OM, Cochi SL, Jafari HS, et al. Possible eradication of wild poliovirus type 3--worldwide, 2012. *Morbidity and Mortality Weekly Report* 2014;63(45):1031-33.
12. World Health Organization. Laboratory surveillance for wild and vaccine-derived polioviruses, January 2004-June 2005. *Weekly Epidemiological Record* 2005;80(39):335-40.

For peer review only

For peer review only - http://bmjopen.bmj.com/site/about/guidelines.xhtml

13. Nnadi C, Damisa E, Esapa L, et al. Continued endemic wild poliovirus transmission in security-compromised areas - Nigeria, 2016. *MMWR Morb Mortal Wkly Rep* 2017;66(7):190-93. doi: 10.15585/mmwr.mm6607a2
14. Nathanson N, Kew OM. From emergence to eradication: the epidemiology of poliomyelitis deconstructed. *American Journal of Epidemiology* 2010;172(11):1213-29. [published Online First: 2010/10/28]
15. World Health Organization. Transmission of wild poliovirus type 2 - Apparent global interruption. *Weekly Epidemiological Record* 2001;76:95-97.
16. Deshpande JM, Nadkarni SS, Siddiqui ZA. Detection of MEF-1 laboratory reference strain of poliovirus type 2 in children with poliomyelitis in India in 2002 & 2003. *Indian Journal of Medical Research* 2003;118:217-23.
17. Duizer E, Ruijs WL, van der Weijden CP, et al. Response to a wild poliovirus type 2 (WPV2)-shedding event following accidental exposure to WPV2, the Netherlands, April 2017. *Euro Surveillance* 2017;22(21):pii=30542. doi: 10.2807/1560-7917.ES.2017.22.21.30542
18. Centers for Disease Control and Prevention. Certification of poliomyelitis eradication--the Americas, 1994. *MMWR Morb Mortal Wkly Rep* 1994;43(39):720-2.
19. Debanne SM, Rowland DY. Statistical certification of eradication of poliomyelitis in the Americas. *Mathematical Biosciences* 1998;150(1):83-103.
20. Eichner M, Dietz K. Eradication of poliomyelitis: when can one be sure that polio virus transmission has been terminated? *American Journal of Epidemiology* 1996;143(8):816-22.
21. Eichner M, Hadeler KP. Deterministic models for the eradication of poliomyelitis: vaccination with the inactivated (IPV) and attenuated (OPV) polio virus vaccine. *Mathematical Biosciences* 1995;127(2):149-66.
22. Eichner M, Hadeler KP, Dietz K. Stochastic models for the eradication of poliomyelitis: minimum population size for polio virus persistence. In: Isham V, Medley GF, eds. Models for Infectious Human Diseases: their structure and relation to data. New York: Cambridge University Press 1996:315-27.
23. World Health Organization. Global Polio Eradication Initiative: Strategic plan 2004-2008. Geneva, 2004.
24. Bahl S, Kumar R, Menabde N, et al. Polio-free certification and lessons learned - South-East Asia region, march 2014. *Morbidity and Mortality Weekly Report* 2014;63(42):941-46.
25. Kalkowska DA, Duintjer Tebbens RJ, Thompson KM. The probability of undetected wild poliovirus circulation after apparent global interruption of transmission. *American Journal of Epidemiology* 2012;175(9):936-49.
26. Houy N. The probability of undetected wild poliovirus circulation: Can we do better? *Journal of Theoretical Biology* 2015;382:272-78.
27. Kalkowska DA, Duintjer Tebbens RJ, Pallansch MA, et al. Modeling undetected live poliovirus circulation after apparent interruption of transmission: Implications for surveillance and vaccination. *BMC infectious diseases* 2015;15(66):1. doi: 10.1186/s12879-015-0791-5
28. Kalkowska DA, Duintjer Tebbens RJ, Thompson KM. Modeling strategies to increase population immunity and prevent poliovirus transmission in two high-risk areas in northern India. *Journal of Infectious Diseases* 2014;210(Suppl 1):S398-S411.

For peer review only - http://bmjopen.bmj.com/site/about/guidelines.xhtml

29. Kalkowska DA, Duintjer Tebbens RJ, Thompson KM. Modeling strategies to increase population immunity and prevent poliovirus transmission in the high-risk area of northwest Nigeria. *Journal of Infectious Diseases* 2014;210(Suppl 1):S412-S23.
30. Duintjer Tebbens RJ, Kalkowska DA, Wassilak SGF, et al. The potential impact of expanding target age groups for polio immunization campaigns. *BMC infectious diseases* 2014;14:45.
31. Kalkowska DA, Duintjer Tebbens RJ, Grotto I, et al. Modeling options to manage type 1 wild poliovirus imported into Israel in 2013. *Journal of Infectious Diseases* 2015;211(11):1800-12.
32. Berchenko Y, Manor Y, Freedman LS, et al. Estimation of polio infection prevalence from environmental surveillance data. *Sci Transl Med* 2017;9(383) doi: 10.1126/scitranslmed.aaf6786
33. Famulare M. Has wild poliovirus been eliminated from Nigeria? . *PLoS One* 2016;10:e0135765.
34. Vallejo C, Keesling J, Koopman JS, et al. Silent circulation of poliovirus in small populations. *Infectious Disease Modelling* 2017;2(4):431-40. doi: 10.1016/j.idm.2017.11.001
35. Vallejo C, Keesling J, Koopman JS, et al. Corrigendum to "Silent circulation of poliovirus in small populations". *Infectious Disease Modelling* 2018;3:136-38. doi: 10.1016/j.idm.2017.11.001
36. Kalkowska DA, Duintjer Tebbens RJ, Thompson KM. Another look at silent circulation of poliovirus in small populations. *Infectious Disease Modelling* 2018;3:107-17. doi: https://doi.org/10.1016/j.idm.2018.06.001
37. World Health Organization. Report on polio transition planning - advance draft as of 18 April, 2017 2017 [Available from: http://www.who.int/mediacentre/events/2017/advance-report-polio-transmission.pdf?ua=1 accessed March 12 2018.
38. Thompson KM, Duintjer Tebbens RJ. Lessons from the polio endgame: Overcoming the failure to vaccinate and the role of subpopulations in maintaining transmission. *Journal of Infectious Diseases* 2017;216(Suppl 1):S176-S82.
39. Gillespie DT. A general method for numerically simulating the stochastic time evolution of coupled chemical reactions. *Journal of Computational Physics* 1976;22:403-34.
40. Sterman J. Business dynamics: Systems thinking and modeling for a complex world. Boston, MA: McGraw-Hill 2000.
41. Duintjer Tebbens RJ, Pallansch MA, Cochi SL, et al. Modeling poliovirus transmission in Pakistan and Afghanistan to inform vaccination strategies in undervaccinated subpopulations. *Risk Analysis* 2018;38(8):1701-17. doi: 10.1111/risa.12962
42. Global Polio Eradication Initiative. Guidelines on environmental surveillance for detection of polioviruses - working draft - March 2015, 2015.
43. Duintjer Tebbens RJ, Zimmermann M, Pallansch MA, et al. Insights from a systematic search for information on designs, costs, and effectiveness of poliovirus environmental surveillance systems. *Food and Environmental Virology* 2017;9(4):361-82. doi: 10.1007/s12560-017-9314-4
44. Anis E, Kopel E, Singer S, et al. Insidious reintroduction of wild poliovirus into Israel, 2013. *Euro Surveillance* 2013;18(38):pii=20586.

For peer review only - http://bmjopen.bmj.com/site/about/guidelines.xhtml

45. Kalkowska DA, Duintjer Tebbens RJ, Thompson KM. Environmental surveillance system characteristics and impacts on confidence about no undetected serotype 1 wild poliovirus circulation. *Risk Analysis* 2018:on-line September 21, 2018, doi: 10.1111/risa.13193.
46. Thompson KM, Wallace GS, Duintjer Tebbens RJ, et al. Trends in the risk of U.S. polio outbreaks and poliovirus vaccine availability for response. *Public Health Reports* 2012;127(1):23-37.
47. Duintjer Tebbens RJ, Thompson KM. Polio endgame risks and the possibility of restarting the use of oral poliovirus vaccine. *Expert Review of Vaccines* 2018;17(8):739-51. doi: 10.1080/14760584.2018.1506333

For peer review only

For peer review only - http://bmjopen.bmj.com/site/about/guidelines.xhtml

Table 1: Model inputs to characterize a hypothetical population that contains an under-vaccinated subpopulation.

Model input	Value(s)[a]	Source/notes
Total population size	500,000;**1 million**;5 million	No effect on DEB model behavior, but required for stochastic analysis of detections
Time until vaccination starts, years<br>General population<br>Under-vaccinated subpopulation	 30<br>40	Assumption to characterize hard-to-reach subpopulation within well-vaccinated general population
Initial age distribution<br>0-2 months<br>3-59 months<br>5-14 years<br>≥ 15 years	 0.01<br>0.15<br>0.25<br>0.59	Equilibrium age distribution[38]
Birth rate, births/person/year	0.02	[38]
Death rate, deaths/person/year	0.02	[38]
Basic reproduction number (R_0)	10	[38]
Proportion of transmissions via oropharyngeal route	0.3	[38]
Proportion of contacts reserved for individuals within the same mixing age group	0.4	Same value as used in [38] (not explicitly listed)
Average per-dose take rate for serotype 1 OPV	0.6	Increased from 0.5 to maintain similar coverage thresholds with different run-up [38]
Routine immunization coverage<br>General population<br>Under-vaccinated subpopulation	 0.95<br>0.75;0.82;0.85;0.90;0.95[b]	Represents coverage with exactly 3 OPV doses; general population based on [38], under-vaccinated varied around threshold to eradicate, which equals 0.82 for the bolded values in the middle column
Proportion of contacts with under-vaccinated subpopulation (p_{within})	0.8;**0.95**;1.00	Selected values from [38]
Size of under-vaccinated subpopulation compared to total population	1/20;**1/10**;1/5	Selected values from [38]
Paralysis-to-infection ratio (PIR)	1/200	Average for serotype 1 wild poliovirus[2,14]
Detection probability per polio case<br>General population<br>Under-vaccinated subpopulations	 0.95<br>0;0.1;0.2;0.3;0.4;0.5;0.6;0.7;0.8;0.9;0.95[b]	Assumption to characterize hard-to-reach subpopulation within general population with high acute flaccid paralysis surveillance quality

Abbreviations: DEB, differential-equation based; OPV, oral poliovirus vaccine

[a] Values shown in bold represent values that we held fixed when varying other values in sensitivity analyses

[b] All values considered jointly in all sensitivity analysis (hence no single value bolded)

For peer review only - http://bmjopen.bmj.com/site/about/guidelines.xhtml

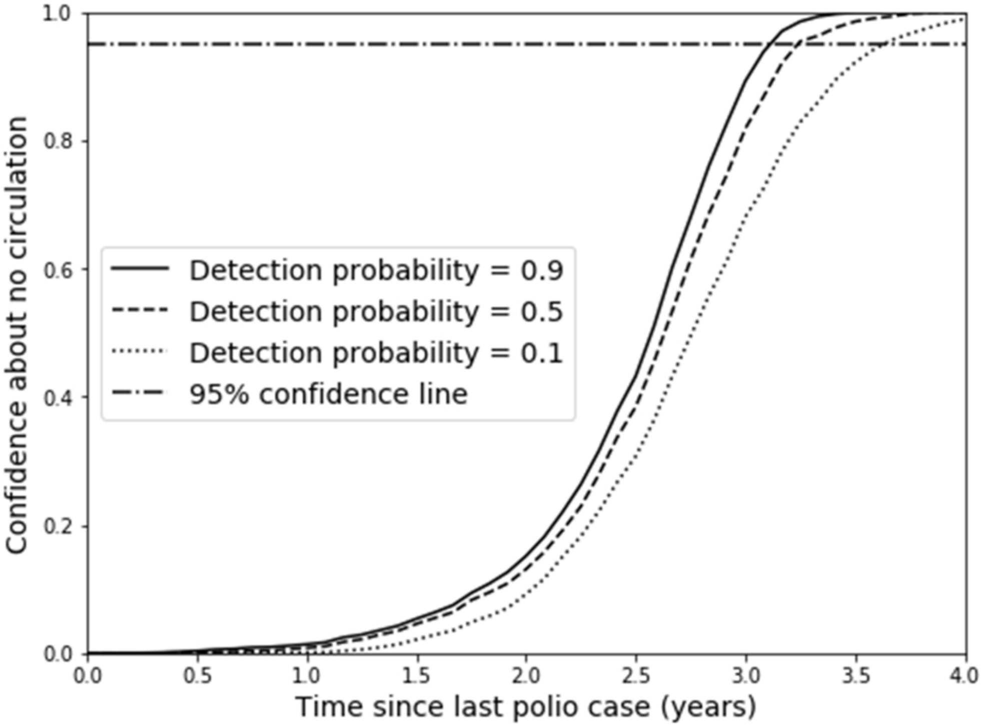

Figure 1: Confidence about no circulation as a function of time since the last detection for different detection probability values for the hypothetical model base case, with coverage at the corresponding minimum to eliminate WPV (i.e., 0.82).

179x133mm (300 x 300 DPI)

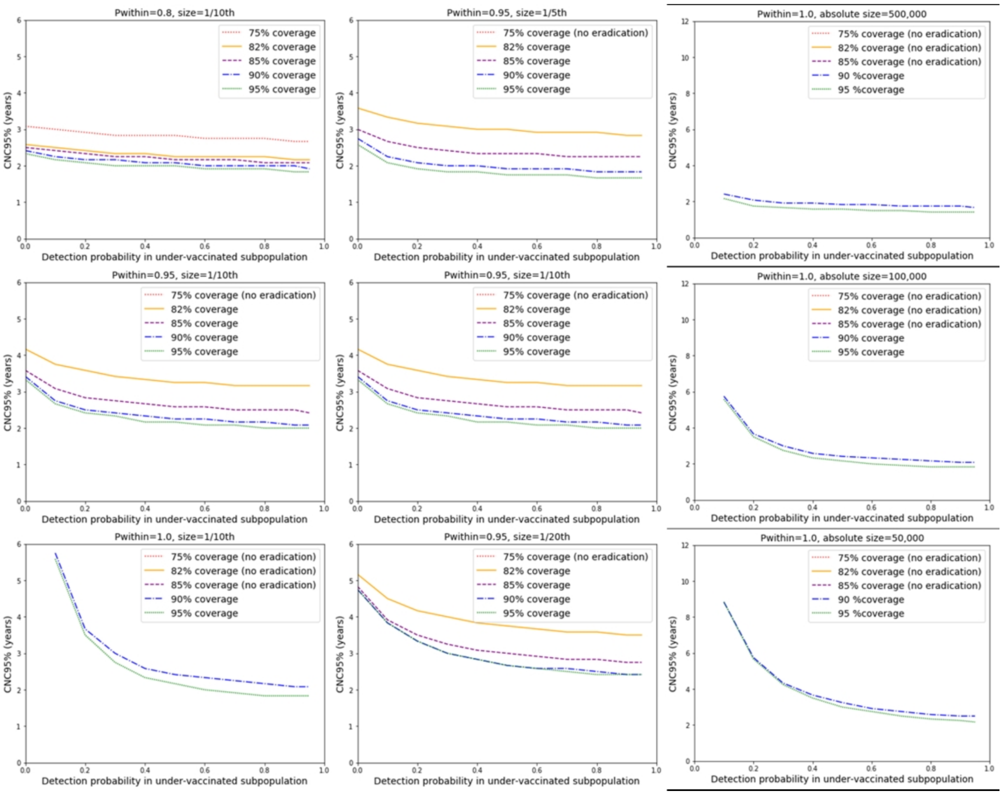

Figure 2: Time until the confidence about no circulation reaches 95% (CNC95%) from the stochastic analysis for different degrees of isolation of the under-vaccinated subpopulation (left column), relative sizes of the under-vaccinated subpopulation (middle column), and absolute sizes of a fully-isolated under-vaccinated subpopulation (right column, note doubled y-axis ranges).

250x196mm (300 x 300 DPI)

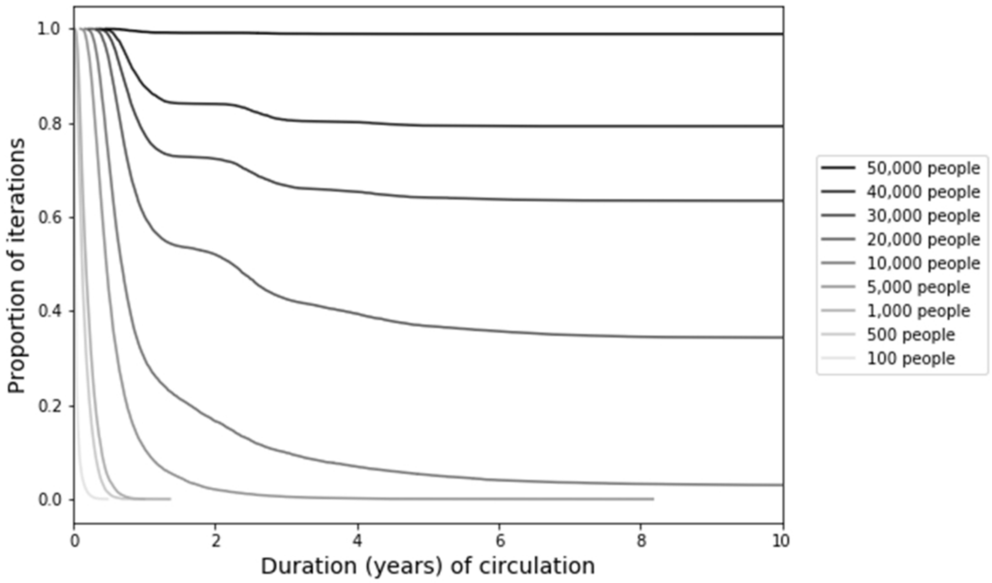

Results from the analysis of the relationship between population size and persistence of circulation of serotype 1 wild poliovirus transmission in the fully stochastic model (a) Model starts at the endemic equilibrium

222x133mm (300 x 300 DPI)

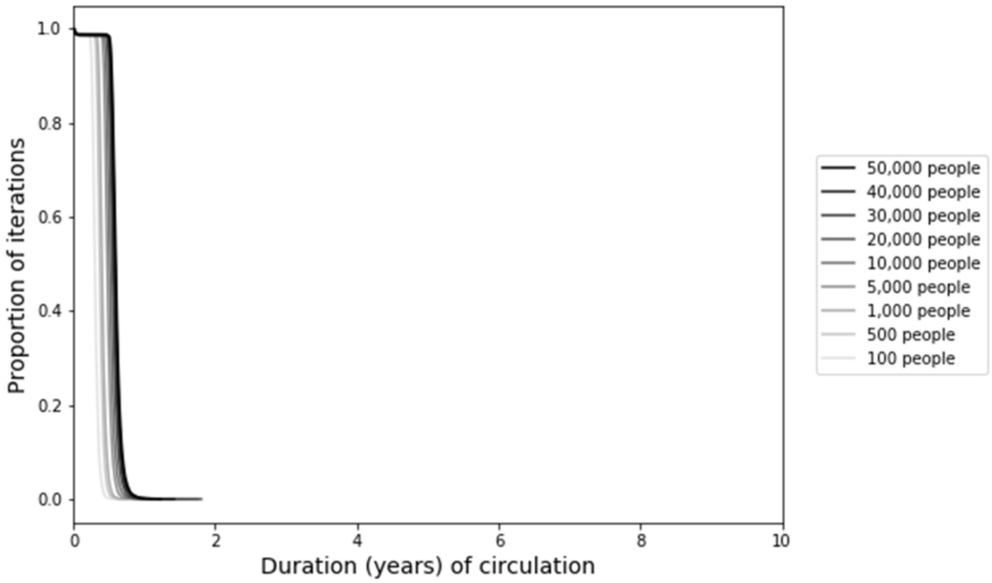

Figure 3: Results from the analysis of the relationship between population size and persistence of circulation of serotype 1 wild poliovirus transmission in the fully stochastic model (b) Model starts with a single infection in a fully susceptible population

222x133mm (300 x 300 DPI)

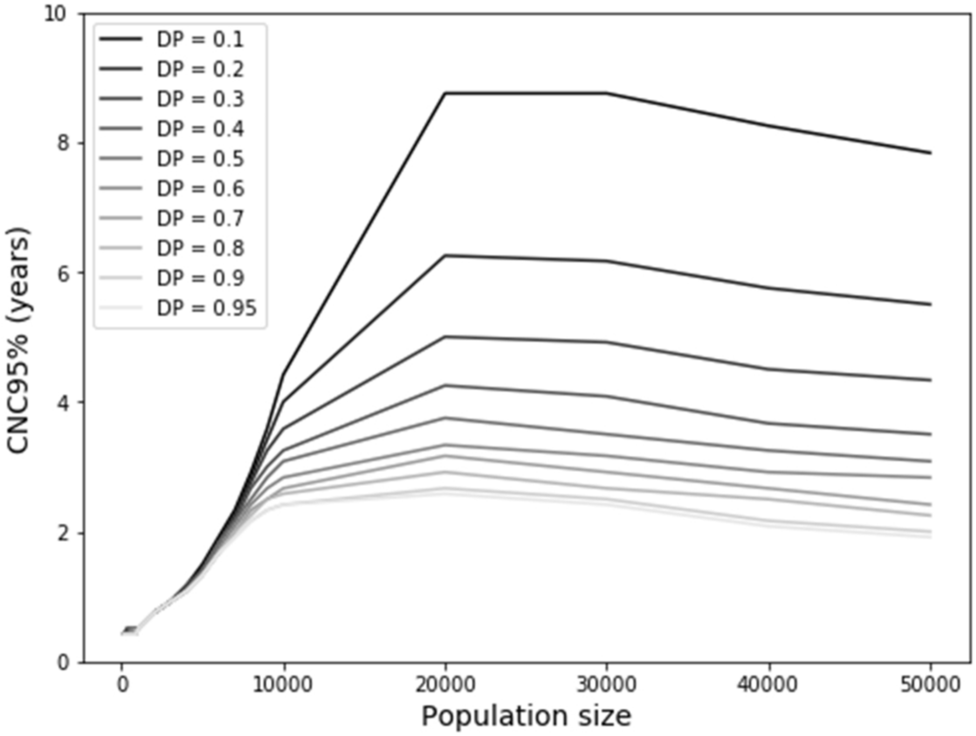

Figure 4: Time until the confidence about no circulation reaches 95% (CNC95%) for small population sizes in the fully stochastic model that starts at the endemic equilibrium, by detection probability (DP)

177x133mm (300 x 300 DPI)

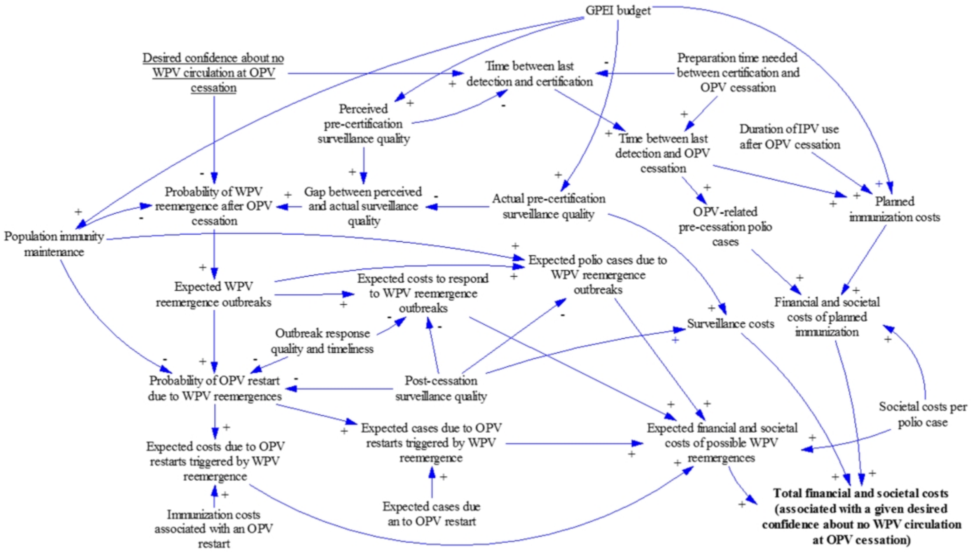

Figure 5: Conceptual diagram for the implications of choices about the timing of certification of eradication of a wild poliovirus serotype on total financial and societal costs

228x135mm (300 x 300 DPI)

APPENDIX for "Certification of global eradication: The role of hard-to-reach subpopulations and confidence about the absence of transmission"

Radboud J. Duintjer Tebbens,[1] Dominika A. Kalkowsa,[1] Kimberly M. Thompson[1]

Differential-equation based model and results

The DEB model we use to examine the role of subpopulations [38] made simplifying assumptions about what a high-risk population might look like and otherwise adopted the comprehensive structure and setting-invariant model inputs of a previously developed and calibrated differential-equation based poliovirus transmission and OPV evolution model.[2 30] The following text from the appendix of a prior publication [45] (with references renumbered) briefly describes the model and Figures A1-2 and Table A1 cited in the text provide the model structure and generic inputs (i.e., model inputs that remain the same for all populations).

"The differential equation-based poliovirus transmission and OPV evolution model (DEB model) [2] tracks the movement of people between demographic age groups (grouped into mixing age groups that mix preferentially amongst themselves), and for each serotype between oropharyngeal and intestinal infection stages (resulting in potential oropharyngeal and fecal-oral transmission, respectively), immunity states, and waning stages. Figure A1 provides an overview of the model structure based on prior work.[2] Figure A1a depicts the immunity states with the flows that move individuals in and out of them and Figure A1b details how effectively vaccinated or infected individuals progress through different stages of infection and, in the event of infection with OPV, through OPV evolution stages. The model assumes that active immunity from prior vaccination or infection results in permanent protection from polio (disease), but only partial protection from subsequent infection and participation in transmission, depending on the nature of immunity (IPV-induced vs. LPV-induced or both) and time since the last exposure (i.e. waning stage). The model includes 5 waning stages, 6 fecal-oral and 6 oropharyngeal infection stages (2 latent and 4 infectious, with varying degrees of infectiousness), and also accounts for a delay between IPV receipt and development of the immune response that moves individuals to the next IPV immunity state. In Figure A1a, we note that the model assumes identical properties for "IPV and LPV" and "≥ 2 LPV infections" and that the recent waning stages of these immunity states represent the highest degree of immunity to transmission in the model. The model further tracks OPV evolution by moving individuals infected with the OPV parent strain (stage 0) through 20 successive reversion stages that can each transmit and that come with increasing paralysis-to-infection ratios and relative basic reproduction numbers (R_0 values) compared to homotypic WPVs. The last reversion stage (stage 19) represents fully-reverted VDPVs with assumed paralysis-to-infection ratio and R_0 equivalent to homotypic WPVs. For WPVs or any OPV reversion stage, the DEB model mimics die-out by setting the force-of-infection for the given strain to 0 whenever its effective prevalence of infections resides below a calibrated threshold of 5 per million people. Consequently, OPV-related viruses can only continue to transmit and thus evolve to cVDPVs through successive infections when low enough population immunity to transmission permits circulation of the OPV viruses introduced in the population through vaccination. We fixed the die-out process, model structure, and numerical

For peer review only - http://bmjopen.bmj.com/site/about/guidelines.xhtml

model inputs that characterize them across all populations we modeled and Table A1 includes the corresponding generic model inputs. […]

"Figure A2 summarizes the results of the model calibration process, based on prior work.[2] With the generic model inputs from Table A1 fixed, we compared our model behavior against i) data on children with non-polio acute flaccid paralysis who reported no receipt of OPV for northern India (modeled separately for Western Uttar Pradesh (WUP) and Bihar) and northwest (NW) Nigeria; ii) data on polio incidence and die-out of endemic WPV transmission for all situations and serotypes (shown in Figure A2 for WPV1 and WPV3 in northern India and northwest Nigeria and for all 3 WPV serotypes in the USA); iii) data from WPV importation outbreak behavior in the Netherlands, Tajikistan, and Albania; iv) data on age distributions of cases for all situations in which meaningful data was available (shown in Figure A2 for the Netherlands, Tajikistan, and Albania); v) available serogical data on the effect of secondary OPV immunity in the USA and Cuba (not shown); vi) indigenous emergence of cVDPVs (shown in Figure A2 for northern India, NW Nigeria (both serotype 2), Haiti, and Madura in Indonesia (both serotype 1); and vii) no indigenous emergence of cVDPVs in all other situations and serotypes (die-out of serotype 1 OPV-related viruses shows in Figure A2 for Cuba and Haiti). We subsequently applied the model to successfully reproduce the asymptomatic transmission of an imported WPV1 in Israel in 2013.[31]" [45, online supplement pp. 1-2]

Most critically in the context of certification questions, the DEB model approximates interruption of live poliovirus transmission (i.e., of an OPV, WPV, vaccine-derived poliovirus (VDPV), or OPV-related strain) in a population to occur when the effective infectiousness-weighted proportion of the population infectious with that poliovirus drops below 5 per million people (i.e., the transmission threshold EPI^{*}).[2] While this simplifies the true die-out behavior, which depends on local heterogeneity and chance, it appears capable of generating WPV die-out times consistent with observations in a broad range of settings.[2 30 31 41] Moreover, when applied to the persistence of OPV-related viruses that evolve to fully transmissible and neurovirulent circulating VDPVs (cVDPVs), the approximation produces cVDPV outbreaks for conditions in which they occurred (e.g., in Hispaniola[46] and Nigeria[47]) and no cVDPV outbreaks for conditions in which they did not occur despite OPV use and cessation (e.g., in Cuba[48] and the USA[49]).[2]

Use of the hypothetical model clarified that under-vaccinated subpopulations can sustain poliovirus transmission independently despite high coverage in the surrounding general population and showed how the minimum coverage needed to interrupt transmission depends on the degree of isolation and the relative size of the under-vaccinated subpopulation.[38] To explore the role of hard-to-reach under-vaccinated subpopulations for certification questions, we modified the hypothetical model in two ways and added a stochastic layer on top of the DEB model to simulate polio case detections. The first modification consisted of desynchronizing the time when vaccination starts in the general and under-vaccinated subpopulations to simulate the concept of a population that remains inaccessible for an extended period of time. Specifically, we run the model, which assumes equal birth and death rates and thus no population growth (Table 1), without vaccination for 30 years to settle into the endemic equilibrium, and then instantly change the routine immunization coverage in the general population with three OPV doses to 0.95, which lies well above the threshold of 0.92 needed to interrupt transmission in a closed population with similar characteristics.[38] However, we assume that the under-vaccinated subpopulation initially remains completely unreached by vaccination, with vaccine introduction

For peer review only - http://bmjopen.bmj.com/site/about/guidelines.xhtml

in the under-vaccinated subpopulation occurring 10 years after vaccine introduction in the general population. Desynchronizing the introduction of vaccination affects the dynamics and effectively makes it more difficult to interrupt transmission after introducing vaccination in the last subpopulation. To offset this effect, we consider a different hypothetical population with a slightly higher average per-dose take rate for OPV of 0.6 instead of 0.5 in the original analysis[38] (e.g., due to lower exposure to enteric viruses that interfere with vaccine take [50]). As in the original analysis,[38] we vary the coverage in the under-vaccinated subpopulation, the relative size of the under-vaccinated subpopulation compared to the total population, and the degree of preferential mixing, characterized by the proportion of potentially infectious contacts of individuals in the under-vaccinated subpopulations with other individuals in the same subpopulation (p_{within}).

Figure A3 shows the behavior of the incidence of infections in fully susceptible individuals and infants born with maternal immunity as a function of the varied DEB model inputs. Generally, the model yields incidence proportional to population size before vaccination starts. After the introduction of vaccination with high coverage in the general population, the initially still unvaccinated subpopulation becomes the main contributor to the total incidence. However, with less than 100% coverage in the general population and some interaction between the two populations (i.e., $p_{within}<1$), some incidence continues to occur in the general population as exported viruses find unvaccinated individuals. Lower values of p_{within} imply more interaction between the two populations and result in more incidence in the general population before vaccination in the under-vaccinated subpopulation begins (middle column of Figure A3). The relative size of the under-vaccinated subpopulation also affects the extent to which the under-vaccinated subpopulation affects the general population (right column of Figure A3). With base case model inputs, the minimum coverage in the under-vaccinated subpopulation to interrupt transmission equals 0.82. Higher coverage values mean interruption occurs sooner after the introduction of vaccination in the under-vaccinated subpopulation, while lower coverage values mean that transmission continues and can eventually rebound and settle into a new equilibrium (left column in Figure A1).

While the prior approach used fully stochastic transmission models to randomly generate infections, die-out, and polio cases and detections,[19-22] for efficiency we use post-hoc processing of DEB model results to randomly generate only the times when polio cases and detections stochastically occur. Specifically, for each setting of the DEB model, we record the deterministic realization of the daily incidence of infections in fully susceptible individuals of any age and 50% of infants less than 3 months of age born with maternal immunity, which represent the only individuals at risk of becoming a polio case in the DEB model.[2] We then randomly determine the number of polio cases resulting from the infection incidence on each day using a Poisson draw with a rate equal to the infection incidence multiplied by the PIR. For each generated case, we use a separate uniform random draw to determine whether it results in a detection based on each of the detection probabilities in Table 1 (e.g., a random uniform draw of 0.45 would mean that the case results in a detection only for detection probabilities of more than 0.45). For each DEB model setting, we repeat the post-hoc stochastic process 10,000 times and we start generating cases 10 years before vaccination starts in the general population, which we assume starts vaccination 10 years earlier than the under-vaccinated subpopulation (see appendix). The precise choice of when to start randomly generating cases exerts negligible

For peer review only - http://bmjopen.bmj.com/site/about/guidelines.xhtml

influence on the results as long as it occurs before cases become rare (i.e., before the interval between cases becomes longer). For simplicity, although prior work showed the significant role of serotype differences and seasonality,[20, 22] the hypothetical model inputs reflect WPV1 and assumes no seasonality. A limitation arises from the direct scaling of the DEB model with absolute population size, such that die-out depends on the effective proportion of infectious individuals rather than the absolute number. Using the post-hoc stochastic analysis, the absolute population size affects the number of infections, which affects the typical interval between detected cases. We show that CNC95% increases substantially for smaller absolute population sizes.

Our initial findings motivated analysis of the minimum population size that can sustain WPV circulation on its own to determine whether the upper bound on the CNC95% of 9 years could occur in real populations. However, for population sizes far below 100,000, the DEB model becomes inadequate because it allows prevalence to remain above the die-out threshold even with only fractional numbers of infections (i.e., less than one infected person). Therefore, we used a fully stochastic model to explore questions of minimum population size. We run the model 10,000 times for different population sizes and initial conditions and report the distribution of the duration of circulation and the CNC95%.

Exploration of the causal interactions relevant to global WPV certification decisions with an influence diagram (Figure 5)

Table A2 provides indicative estimates of the key quantities in Figure 5, based on the literature. Figure 5 assumes that policy makers explicitly or implicitly set a *desired confidence about no WPV circulation at OPV cessation*. In reality, they may focus on the confidence at certification, but given that it takes some fixed *preparation time needed between certification and OPV cessation*, any set confidence at the time of certification corresponds to some *desired confidence about no WPV circulation at OPV cessation.* A higher desired confidence level implies a longer *time between last detection and certification.* This time decreases with increasing investments in immunization and surveillance from the *GPEI budget* through *population immunity maintenance* and the *perceived pre-certification surveillance quality*, respectively. The main drawback of a longer *time between last detection and OPV cessation* comes in the form of longer OPV use in most countries, which results in *planned immunization costs* and *OPV-related pre-cessation polio cases* (i.e., vaccine-associated paralytic polio and VDPVs). In addition, with some globally-recommended or nationally-preferred *duration of IPV after OPV cessation,* later OPV cessation would imply greater overall IPV costs, because global IPV use already started (i.e., only the end, and not the beginning of IPV use depends on the timing of cessation of the last OPV serotypes). These drawbacks together lead to *financial and societal costs of planned immunization*. This includes the monetary equivalent of the *OPV-related polio cases*, which depends on the country income-level-dependent *societal costs per polio case.*

On the left side of Figure 5, we see the benefits of setting a higher *desired confidence about no WPV circulation at OPV cessation.* A higher confidence implies a lower *probability of a WPV reemergence after OPV cessation* (all else being equal). However, this probability does not directly equal the reciprocal of the confidence in the event of a *gap between perceived and actual surveillance quality.* Specifically, if the *perceived pre-certification surveillance quality* exceeds

For peer review only - http://bmjopen.bmj.com/site/about/guidelines.xhtml

the *actual pre-certification surveillance quality*, then the true *probability of WPV reemergence after OPV cessation* equals more than 1 minus the *desired confidence about no WPV circulation at OPV cessation*, and vice versa. This potential discrepancy highlights the importance of continued assessment of surveillance quality and assurance of high surveillance quality. A lower *GPEI budget* also decreases *population immunity maintenance* and thus increases the *probability of WPV reemergence after OPV cessation*, which implies an increase in *expected WPV reemergence outbreaks.* Unlike other possible types of post-cessation outbreaks, a WPV reemergence would almost certainly occur in the most challenging populations. Any such reemergences would lead to *expected polio cases due to WPV reemergence outbreaks* and *expected costs to respond to WPV emergence outbreaks*. The expected costs and cases decrease with higher *post-cessation surveillance quality*, which affects the extent of viral spread at the time of outbreak detection (and beyond), and with a better *outbreak response quality and timeliness,* which both increase the probability of effective outbreak control.[51] However, the occurrence of any outbreaks comes with some probability of uncontrolled outbreaks, either by failing to control the original outbreak virus, or by creating new cVDPV outbreaks with the OPV vaccine used in the response. This implies some *probability of OPV restart due to WPV reemergences*, which would carry very significant *expected costs due to an OPV restart triggered by WPV reemergence* and *expected cases due to an OPV restart triggered by WPV emergence* (Table A2). For moderate or high *probability of OPV restart due to WPV reemergences*, the resulting *expected costs due to OPV restarts triggered by WPV reemergence* and *expected cases due to OPV restarts triggered by WPV reemergence* would likely dwarf the costs and cases associated with any controlled outbreaks due to WPV reemergences and would therefore drive the *expected financial and societal costs of possible WPV reemergences.*

Together with the *surveillance costs*, which act to moderate the costs of delayed OPV cessation or premature OPV cessation, the *expected financial and societal costs of possible WPV reemergences* and the *financial and societal costs of planned immunization* together make up the *total financial and societal costs (associated with any given desired confidence about no WPV circulation at OPV cessation)*. The costs of possible WPV emergences and the costs of planned immunization move in opposite directions as a function of the *desired confidence about no circulation at OPV cessation.*

Figure 5 also highlights the consequences of the GPEI already scaling down some of its supplemental immunization and surveillance activities. While scaling down saves costs in the short term, doing so could lead to larger long-term costs by delaying certification and OPV cessation (i.e., requiring higher confidence about no circulation), which would imply that OPV cessation could occur in the context of lower global population immunity to transmission and lower ability to rapidly detect outbreaks. This ultimately implies an increase in the expected *total financial and societal costs (associated with any given desired confidence about no WPV circulation at OPV cessation)*. For visual simplicity, Figure 5 omitted some additional complexity involved in this decision. Furthermore, given that the confidence about no circulation increases with time after the last detection, we could have equivalently centered Figure 5 around finding the optimal time between the last detection and certification or OPV cessation. The amounts in Table A2 highlight the significant financial and humanitarian stakes involved in finding the optimal *desired confidence about no WPV circulation at OPV cessation.*

For peer review only - http://bmjopen.bmj.com/site/about/guidelines.xhtml

Table A1: Generic inputs of the DEB model[2 30] (adopted from the online supplement of Duinter Tebbens et al., 2017[45])

Model input (symbol)	Best estimate	Source
Relative susceptibility (σ) of recent immunity states (for PV1;PV2;PV3)		[52 53]
- Maternally immune	0.78;0.79;0.77	
- 1 successful IPV	0.91;0.92;0.90	
- 2 successful IPV	0.80;0.80;0.79	
- ≥ 3 successful IPV	0.72;0.72;0.71	
- 1 LPV infection	0.42;0.43;0.41	
- ≥ 2 LPV infections	0.21;0.22;0.20	
- IPV and LPV	0.21;0.22;0.20	
Duration of latent period (ξ^{fec} or ξ^{oro}, in days)	~ 3[a]	[52 53]
Duration of fecal infectiousness (γ^{fec}, in days) of recent immunity states (for PV1;PV2;PV3)		[52 53]
- Fully susceptible	28.0;27.8;28.3	
- Maternally immune	24.6;24.6;24.6	
- 1 successful IPV,	24.5;24.4;24.7	
- 2 successful IPV	21.1;20.8;21.3	
- ≥ 3 successful IPV	18.0;17.7;18.2	
- 1 LPV infection	11.6;10.5;10.5	
- ≥ 2 LPV infections	10.1;8.9;8.9	
- IPV and LPV	10.1;8.9;8.9	
Duration of oropharyngeal infectiousness (γ^{oro}, in days) of recent immunity states (no serotype differences)		[52 53]
- Fully susceptible	13.4	
- Maternally immune	11.9	
- 1 successful IPV	9.9	
- 2 successful IPV	6.6	
- ≥ 3 successful IPV	6.1	
- 1 LPV infection	5.0	
- ≥ 2 LPV infections	3.7	
- IPV and LPV	3.7	
Relative fecal infectiousness (π^{fec}) of recent immunity states (for PV1;PV2;PV3)		[52 53]
- Maternally immune	0.96;0.96;0.95	
- 1 successful IPV	0.92;0.92;0.91	
- 2 successful IPV	0.70;0.69;0.68	
- ≥ 3 successful IPV	0.61;0.59;0.59	
- 1 LPV infection	0.39;0.43;0.43	
- ≥ 2 LPV infections	0.20;0.23;0.23	
- IPV and LPV	0.20;0.23;0.23	
Relative oropharyngeal infectiousness (π^{oro}) of recent immunity states (no serotype differences)		[52 53]
- Maternally immune	0.68	
- 1 successful IPV	0.30	
- 2 successful IPV	0.17	
- ≥ 3 successful IPV	0.12	
- 1 LPV infection	0.33	
- ≥ 2 LPV infections	0.21	
- IPV and LPV	0.21	
Number of infection stages		
- Latent period (r)	2	
- Infectious period (s)	4	
Relative weight of infection stages, compared to average weight over the infectious period (θ_j, j=0,…,r+s-1)		[52 53]

For peer review only - http://bmjopen.bmj.com/site/about/guidelines.xhtml

- Infection stage 0 and 1 (latent stages)<br>- Infectious stage 2<br>- Infectious stage 3<br>- Infectious stage 4<br>- Infectious stage 5	0<br>12/17<br>40/17<br>12/17<br>4/17	
IPV immunity delay (φ, in days)	7	54
Number of waning stages (*nw*)	5	
Shape of waning function (z_w)	5	52 53
Average time to reach last waning stage (ρ, in days)<br>- Type 1&2<br>- Type 3	 4×365<br>3×365	52 53
Average time for maternal immunes to wane to fully susceptible (ρ_{MI}, in days)	0.25×365	52 53
Relative susceptibility (σ) for last waning stage (no serotype differences)<br>- 1 successful IPV<br>- 2 successful IPV<br>- ≥ 3 successful IPV<br>- 1 LPV infection<br>- ≥ 2 LPV infections<br>- IPV and LPV	 1.0<br>1.0<br>1.0<br>0.8<br>0.7<br>0.7	52 53
Duration of fecal infectiousness (γ^{fec}, in days) of last waning stage (for PV1;PV2;PV3)<br>- 1 successful IPV<br>- 2 successful IPV<br>- ≥ 3 successful IPV<br>- 1 LPV infection<br>- ≥ 2 LPV infections<br>- IPV and LPV	 26.6;26.4;26.9<br>25.2;25.0;25.5<br>23.8;23.6;24.1<br>14.0;13.9;14.1<br>11.4;11.4;11.6<br>11.4;11.4;11.6	52 53
Duration of oropharyngeal infectiousness (γ^{oro}, in days) of last waning stage (no serotype differences)<br>- 1 successful IPV<br>- 2 successful IPV<br>- ≥ 3 successful IPV<br>- 1 LPV infection<br>- ≥ 2 LPV infections<br>- IPV and LPV	 11.4<br>6.7<br>6.6<br>6.7<br>4.0<br>4.0	52 53
Relative fecal infectiousness (π^{fec}) of last waning stage (no serotype differences)<br>- 1 successful IPV<br>- 2 successful IPV<br>- ≥ 3 successful IPV<br>- 1 LPV infection<br>- ≥ 2 LPV infections<br>- IPV and LPV	 0.95<br>0.9<br>0.85<br>0.5<br>0.3<br>0.3	52 53
Relative oropharyngeal infectiousness (π^{oro}) of last waning stage (no serotype differences)<br>- 1 successful IPV<br>- 2 successful IPV<br>- ≥ 3 successful IPV<br>- 1 LPV infection<br>- ≥ 2 LPV infections<br>- IPV and LPV	 0.43<br>0.25<br>0.13<br>0.5<br>0.3<br>0.3	52 53
Number of reversion stages (*h*)	20	
Shape of reversion function with respect to:<br>- R_0 (z_r)<br>- ln(PIR) (z_p)	 1<br>2.5	

For peer review only - http://bmjopen.bmj.com/site/about/guidelines.xhtml

Average time to reach last reversion stage (ε, in days) (for PV1;PV2;PV3)	620.5; 408; 620.5	[30]
Paralysis-to-infection ratio for fully susceptible individuals infected with OPV (PIR_0) (for PV1; PV2;PV3)	0.26×10^{-6}; 1.2×10^{-6}; 1.8×10^{-6}	
Paralysis-to-infection ratio for fully susceptible individuals infected with FRPV (PIR_{h-1}) (for PV1; PV2;PV3)	0.005; 0.0005; 0.001	[2 14 54]
Relative R_0 of OPV vs. FRPV (τ_0) (for PV1; PV2; PV3)	0.37;0.55;0.25	[2 52 53]
Effective infectious proportion below which we assume 0 force-of-infection (transmission threshold EPI*)	5/1,000,000	
Relative PIR for maternally immunes compared to fully susceptible individuals ($RPIR_{MI}$)	0.5	
Ratio of R_0 by serotype in the same setting (PV1:PV2:PV3)	1:0.9:0.75	[30]
Average incubation period (δ, in days)	10	[54 55]
Demographics for all situations	Time series 1950-2100	[56]

Acronyms: CDC = (U.S.) Centers for Disease Control and prevention; cVDPV = circulating vaccine-derived poliovirus; DEB = differential equation-based FRPV = fully-reverted poliovirus; GPLN = Global Polio Laboratory Network; IPV = inactivated poliovirus vaccine; LPV = live poliovirus; OPV = oral poliovirus vaccine; PIR = paralysis-to-infection ratio; PV(1,2,3) = poliovirus (type 1, 2, or 3, respectively); R_0 = basic reproductive number; UN = United Nations; USA = United States of America; VAPP = vaccine-associated paralytic poliomyelitis; VP1 = viral protein 1; WPV(1,2,3) = wild poliovirus (type 1, 2, or 3, respectively)

Notes: [a] Mean estimates obtained from experts and used in the model for the different immunity states, serotypes, and excretion modes vary between 2.85 and 3.37 days

For peer review only

Table A2: Indicative estimates of key variables from Figure 5

Variable	Estimate	Notes and sources
Preparation time needed between certification and OPV cessation	Approximately 1 year	Depends on when setting of the OPV cessation date occurs relative to certification [57]
Planned immunization costs	$1 billion in external GPEI funds per year, plus internal contributions	Most of the $1.1 billion GPEI budget for 2016 was for immunization and coordination of activities;[58] Countries may internally contribute at a similar rate as the external contributions;[59] The current GPEI budget projects a decrease from 2018 forward, which would imply some offset of costs for maintenance of activities, or alternatively the activities previously supported by external contributions may end, which would imply declines in programmatic activities and quality
OPV-related polio cases	Hundreds per year	Vaccine-associated paralytic polio cases,[60] which depends on timing of IPV doses,[61] and presumably local cVDPV outbreaks[62]
Surveillance costs	Around $100 million per year	The 2016 GPEI budget included $67 million in external support for surveillance and laboratories,[58] with additional significant internal contributions by countries [59 63]
Probability of OPV restart due to WPV reemergence	Unknown	Prior studies estimated an approximately 5% chance of an OPV restart due primarily to OPV-associated risks, although the actual implementation of risk management policies was not as good as suggested by these models.[59 64]
Immunization costs associated with an OPV restart	$ billions (hundreds of millions per year)	An OPV restart would involve reintroduction of OPV vaccination in most countries in perpetuity, with supplemental immunization activities needed in countries with insufficient routine immunization coverage.[59] Significant uncertainty exists about what an OPV restart would look like in practice.
Expected cases due to an OPV restart	Up to thousands per year	Reintroduction of OPV in most countries would result in hundreds of vaccine-associated paralytic polio cases per year and could result in continued cVDPV outbreaks in countries with insufficient routine immunization coverage that do no conduct regular preventive supplemental immunization activities.[59 64]

For peer review only - http://bmjopen.bmj.com/site/about/guidelines.xhtml

Figure A1: Schematic of the DEB model structure, adopted from Duintjer Tebbens et al. (2013)[2, p. 706]

(a) Immunity states and flows between them due to epidemiological events

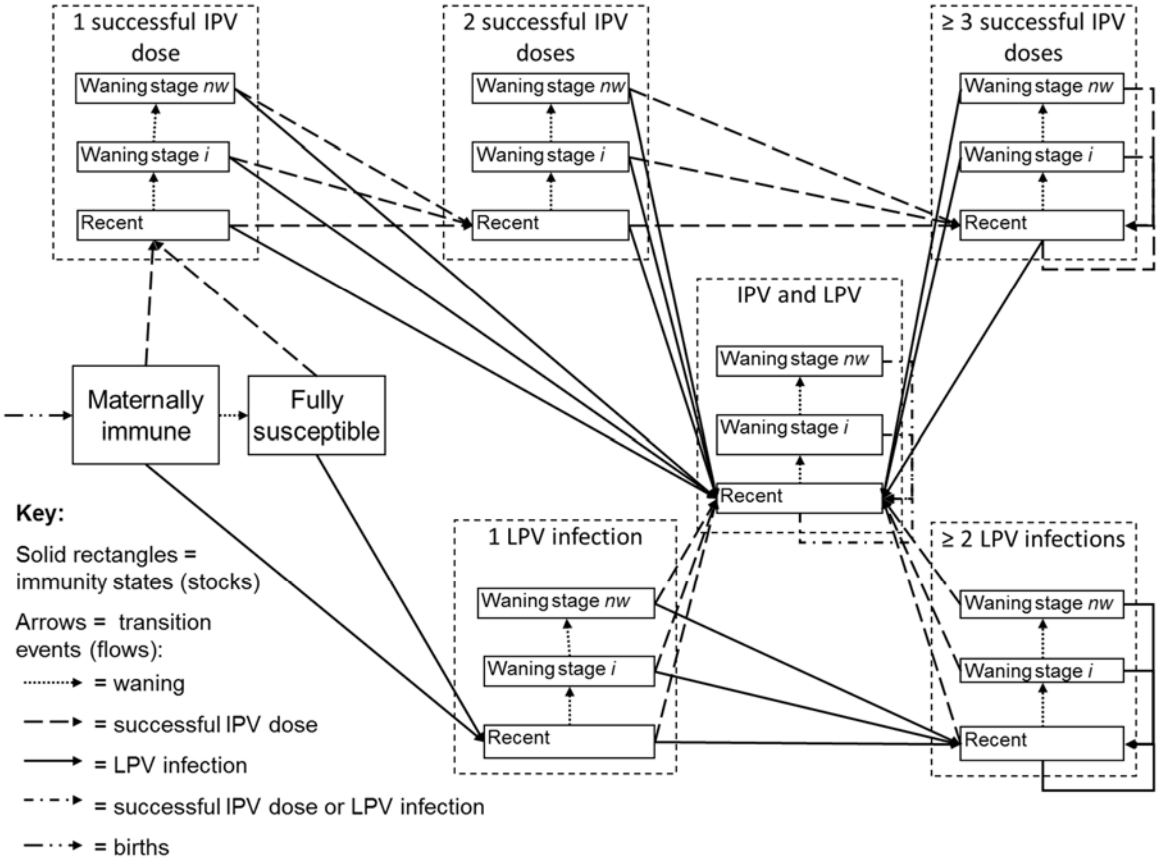

For peer review only - http://bmjopen.bmj.com/site/about/guidelines.xhtml

(b) Progression through infection and reversion stages

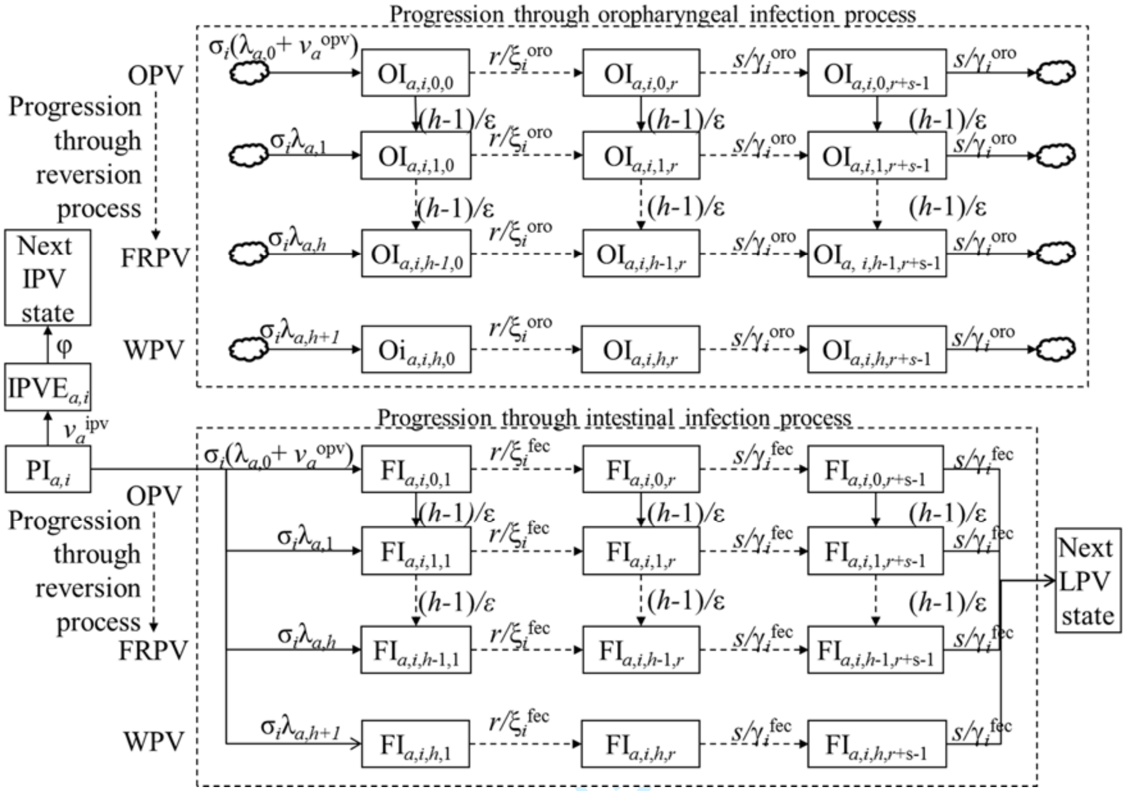

"**Acronyms:** FRPV = fully-reverted poliovirus; IPV = inactivated poliovirus vaccine; OPV = oral poliovirus vaccine; WPV = wild poliovirus**; Symbols:** $PI_{a,i}$ = partially infectible in age group *a* and immunity state *I*; $IPVE_{a,i}$ = IPV-exposed individual from immunity state *i* and age group *a*; $FI_{a,i,j,k}$ ($OI_{a,i,j,k}$) = individual in age group a from immunity state *i*, infected with virus strain *j* and in fecal (oropharyngeal) infection stage *k*; $\lambda_{a,j}$ = force-of–infection to age group *a* for virus strain *j;* ν_a^{ipv} (ν_a^{opv}) = force-of-IPV(OPV)-vaccination to age group *a* as a result of routine and supplementary immunization; σ_i = relative susceptibility for immunity state *i*; ξ_i^{fec} (ξ_i^{oro}) = average duration of the fecal (oropharyngeal) latent period for immunity state *i*; γ_i^{fec} (γ_i^{oro}) = average duration of the fecal (oropharyngeal) infectious period for immunity state *i*; *φ* = IPV immunity delay; *h* = number of reversion stages; *r* = number of latent stages; *s* = number of infectious stages" [2, p. 706]

For peer review only - http://bmjopen.bmj.com/site/about/guidelines.xhtml

Figure A2: Summary results from the model calibration process, adapted from Duintjer Tebbens et al. (2013)[2]

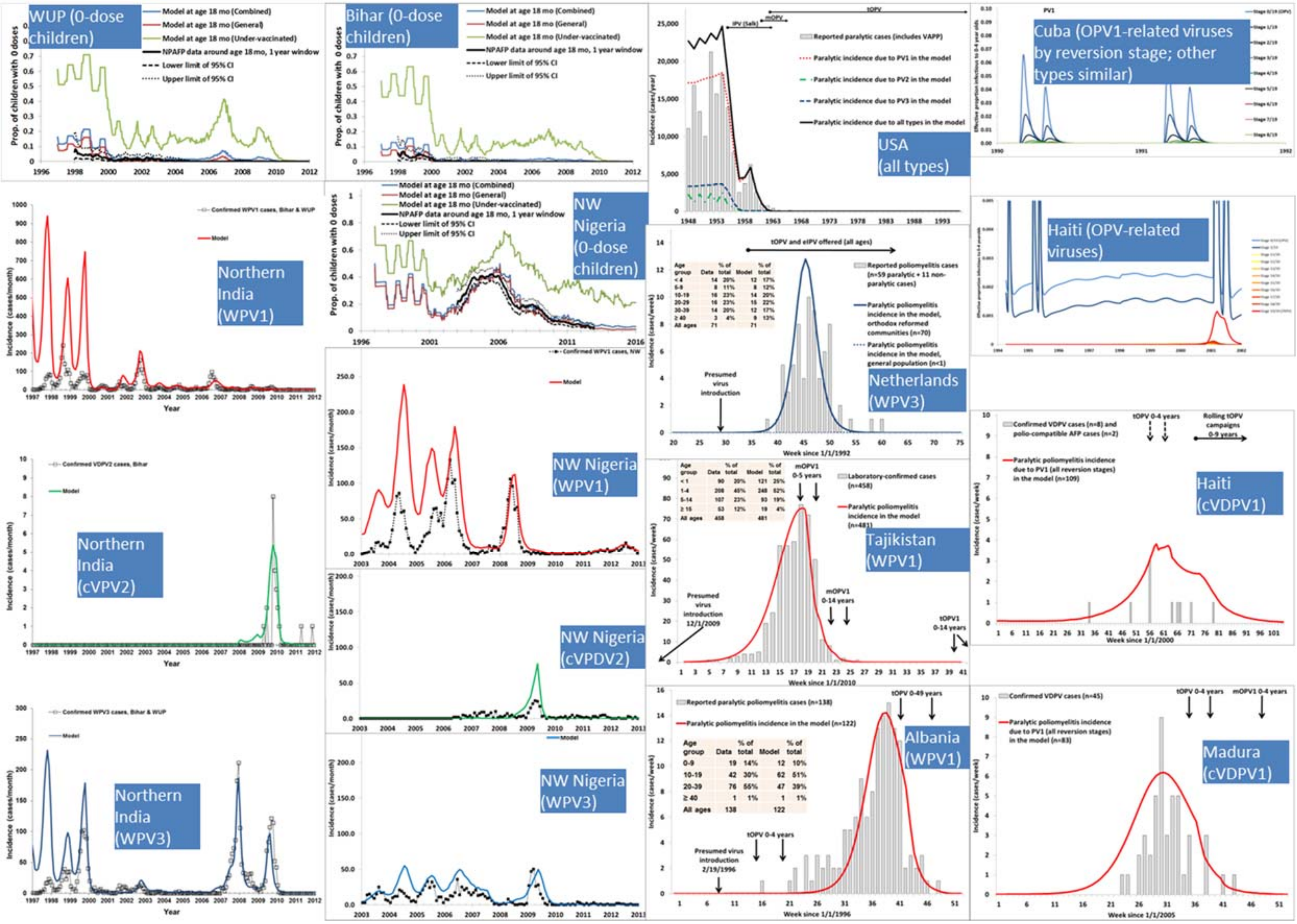

Figure A3: Differential-equation based model results for base case model inputs and varied coverage (left column), varied degree of isolation with coverage 0.82 (middle column), and varied relative size with coverage of 0.82 (right column). The y-axis scales linearly with total population size (all figures assume a total population size of 1 million).

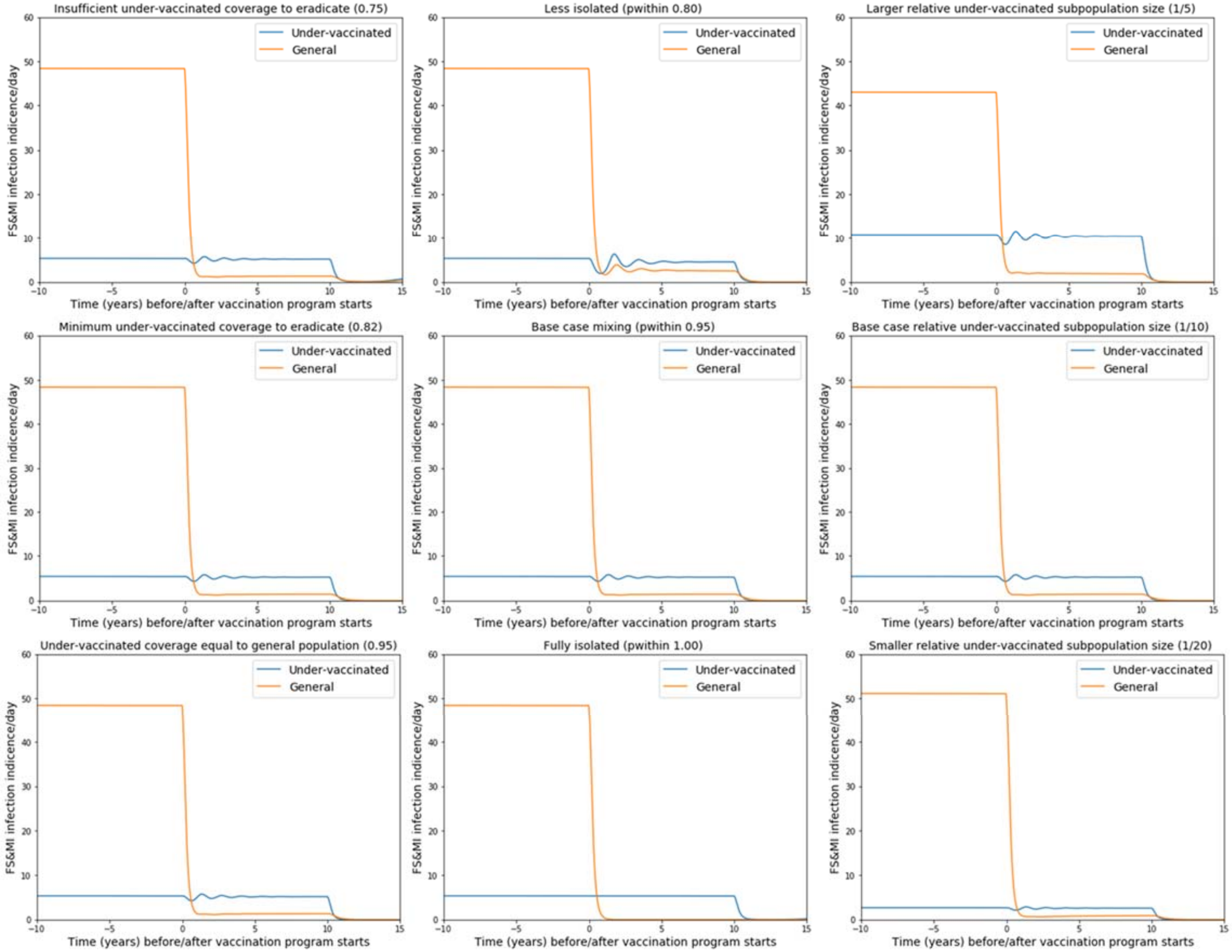

BMJ Open

Global certification of wild poliovirus eradication: Insights from modeling hard-to-reach subpopulations and confidence about the absence of transmission

Journal:	*BMJ Open*
Manuscript ID	bmjopen-2018-023938.R2
Article Type:	Research
Date Submitted by the Author:	29-Nov-2018
Complete List of Authors:	Duintjer Tebbens, Radboud; Kid Risk, Inc.<br>Kalkowska, Dominika; Kid Risk, Inc.<br>Thompson, KM; Kid Risk, Inc.,
**Primary Subject Heading**:	Infectious diseases
Secondary Subject Heading:	Health policy
Keywords:	polio, eradication, certification, modeling

SCHOLARONE™ Manuscripts

Global certification of wild poliovirus eradication: Insights from modeling hard-to-reach subpopulations and confidence about the absence of transmission

Radboud J. Duintjer Tebbens,[1] Dominika A. Kalkowska,[1] Kimberly M. Thompson[1]

1. Kid Risk, Inc., Columbus, OH, USA

Running title: **Poliovirus certification confidence**

Correspondence to: Kimberly M. Thompson, Kid Risk, Inc., 605 N High St, #253, Columbus, OH 43215, USA, Email: kimt@kidrisk.org

Abstract:

Objective: To explore the extent to which under-vaccinated subpopulations may influence the confidence about no circulation of wild poliovirus (WPV) after the last detected case.

Design and participants: We used a hypothetical model to examine the extent to which the existence of an under-vaccinated subpopulation influences the confidence about no WPV circulation after the last detected case as a function of different characteristics of the subpopulation (e.g., size, extent of isolation). We also used the hypothetical population model to inform the bounds on the maximum possible time required to reach high confidence about no circulation in a completely-isolated and unvaccinated subpopulation starting either at the endemic equilibrium or with a single infection in an entirely susceptible population.

Results: It may take over three years to reach 95% confidence about no circulation for this hypothetical population despite high surveillance sensitivity and high vaccination coverage in the surrounding general population if: (1) ability to detect cases in the under-vaccinated subpopulation remains exceedingly small, (2) the under-vaccinated subpopulation remains small and highly isolated from the general population, and (3) the coverage in the under-vaccinated subpopulation remains very close to the minimum needed to eradicate. Fully-isolated hypothetical populations of 4,000 people or less cannot sustain endemic transmission for more than 5 years, with at least 20,000 people required for a 50% chance of at least 5 years of sustained transmission in a population without seasonality that starts at the endemic equilibrium.

For peer review only - http://bmjopen.bmj.com/site/about/guidelines.xhtml

Notably, however, the population size required for persistent transmission increases significantly for realistic populations that include some vaccination and seasonality and/or that do not begin at the endemic equilibrium.

Conclusions: Significant trade-offs remain inherent in global polio certification decisions, which underscore the need for making and valuing investments to maximize population immunity and surveillance quality in all remaining possible WPV reservoirs.

Strengths and limitations of this study:

- Models the limited but important role of under-vaccinated subpopulations in achieving confidence about no WPV transmission after the last reported case.
- Explores trends in transmission and detection for different population sizes as time increases since the last reported case.
- Examines the importance of maximizing population immunity and surveillance quality.
- Provides critical information to support decisions related to the ultimate certification of wild poliovirus elimination.
- Analyses remain limited by model assumptions, but in abstract provide insights relevant to likely last poliovirus reservoirs.

Keywords: polio, eradication, certification, modeling

For peer review only

Background

Achieving the 1988 World Health Assembly polio eradication goal of ending all cases of poliomyelitis [1] requires a successful transition from the interruption of the current low level of wild poliovirus (WPV) transmission through coordinated cessation of all use of live attenuated oral poliovirus vaccine (OPV) to effective long-term risk management. The Global Polio Laboratory Network supports the Global Polio Eradication Initiative (GPEI) by testing stool samples from acute flaccid paralysis (AFP) cases and sewage samples for polioviruses. As the GPEI approaches success, the transition to the polio endgame has begun. The endgame involves significant complexity, because all countries must achieve and maintain sufficient population immunity [2-4] to stop and prevent the transmission of three separate poliovirus serotypes (i.e., 1, 2, and 3) and globally coordinate cessation of each OPV serotype.[5-7] In September 2015, the Global Certification Commission declared successful eradication of serotype 2 WPV (WPV2),[8] which represented a prerequisite to the globally-coordinated cessation of all serotype 2-containing OPV use. Global cessation of serotype 2-containing OPV occurred in late April and early May 2016, during which time over 150 countries stopped using trivalent OPV (tOPV, which contains all three serotypes) and switched to bivalent OPV (bOPV, which contains only serotypes 1 and 3 OPV).[9]

The Global Polio Laboratory Network reported the lowest number of annual paralytic serotype 1 WPV (WPV1) cases in 2017,[10] and no serotype 3 WPV (WPV3) cases since November 2012.[11] Successful WPV eradication requires stopping all transmission, which manifests as an absence of detected WPVs despite actively looking. With increasing time of not seeing cases (while actively looking), confidence increases about WPV die-out. However, the absence of evidence is not evidence of absence. Extended silent transmission can occur, because most poliovirus infections do not lead to symptoms and surveillance gaps can exist. For example, a WPV3 resurfaced in Sudan/Chad in 2004 after no reported cases during 1997-2003 [12] and a WPV1 resurfaced in Borno, Nigeria in 2016 after nearly 3 years with no reported cases [13]. The average paralysis-to-infection ratio (PIR), defined as the fraction of infections in fully susceptible individuals that leads to paralytic poliomyelitis (polio) symptoms, equals approximately 1/200, 1/2000, and 1/1000, for serotype 1, 2, and 3 WPV, respectively.[14] The last reported naturally-

For peer review only - http://bmjopen.bmj.com/site/about/guidelines.xhtml

occurring WPV2 case occurred in India in 1999,[15] and since then, only two episodes of WPV2 infections occurred that traced back to laboratory strains.[16 17] Despite the possibility of silent circulation, the absence of any naturally-occurring WPV2 cases for over 15 years (and in many countries for many decades) led to very high confidence about the die-out of WPV2 transmission.

Multiple prior mathematical modeling studies explored the probability of undetected circulation of WPVs in the absence of reported cases or other poliovirus detections. Polio eradication efforts in the Americas, which reported the last indigenous WPV case of any serotype in Peru in 1991,[18] motivated the first analysis and discussion of certification requirements. A statistical analysis of Pan American Health Organization epidemiological data reported less than a 5% chance of undetected indigenous WPV circulation after 4 years since the last reported confirmed case.[19] A simple, stochastic model of poliovirus transmission and die-out characterized the probability of undetected poliovirus circulation in a hypothetical, homogeneously mixed population of 200,000 people in a relatively low-income country, and estimated that not observing a case for 3 years provided 95% confidence about local extinction of WPV infections.[20] This seminal paper provided the foundation for appropriate characterization of the probability of undetected circulation as a function of the time since the last detected case.[20] Related modeling also explored theoretical thresholds to stop transmission [21] and estimated a minimum population size for persistent transmission of 50,000-100,000 in developing countries and over 200,000 in developed countries required to achieve at least 95% probability of poliovirus persistence for 5 years or more in the absence of vaccination.[22] These studies supported the 2004-8 GPEI Strategic Plan requirement of at least 3 years of no polio cases detected by AFP surveillance for certification,[23] which remains the current minimum requirement.[24] A 2012 study [25] relaxed some of the assumptions of the prior theoretical model [20] and highlighted that the probability of undetected circulation varied for different poliovirus serotypes, places, and conditions, which suggested the need to focus on appropriate characterization of conditions in the last likely WPV reservoirs.[25] A 2015 study [26] also used the prior model [20] to show that in the context of an instantaneous introduction of vaccination, the time of the last case relative to vaccine introduction further informs the confidence about the absence of circulation.

For peer review only - http://bmjopen.bmj.com/site/about/guidelines.xhtml

Subsequent analyses focused on modeling the conditions in specific and more realistic populations. A 2015 study [27] used a previously-developed poliovirus dynamic transmission model [2] applied to: recently-endemic transmission in two states in northern India,[28] endemic transmission in northwest Nigeria,[29] a 2010 outbreak in Tajikistan,[30] and transmission following a 2013 WPV1 introduction into Israel detected by environmental surveillance.[31] The study characterized the confidence about no undetected poliovirus circulation by serotype as a function of time without reported polio cases or environmental detections considering realistic assumptions for surveillance, immunization, and other national inputs.[27] The results suggested that time periods of 0.5 to 3 years without detected polio cases provided 95% confidence about the interruption of transmission in the context of perfect AFP surveillance depending on situation-specific characteristics (e.g., the overall population immunity, endemic versus outbreak conditions, and virus serotype).[27] This model also suggested longer times required for less-than-perfect AFP surveillance and potentially shorter times using highly-sensitive environmental surveillance based on the experience in Israel.[27] A recent statistical analysis of the 2013 WPV1 outbreak in Israel demonstrated a rapid increase in confidence about no undetected local transmission following outbreak response immunization after repeated negative environmental surveillance samples in a city.[32] A non-dynamic, statistical model [33] estimated a shorter time (compared to the 2015 study [27]) of 14 months required to reach high confidence about no undetected circulation. For its most conservative assumptions about surveillance and force-of-infection, the study estimated a probability of 93% of a WPV-free Africa in the absence of any new WPV cases reported by the end of 2015,[33] shortly before the WPV reemerged.[13] Contrasting with all other modeling studies, a recent study [34] suggested a relatively high probability of undetected circulation after more than 3 years without any polio cases in small populations, although a correction to that analysis emphasized the unrealistic nature of one of the assumptions.[35] Remarkably, the analysis reported that closed populations of 10,000 people or fewer could support many years of transmission in the absence of vaccination, and experience gaps between polio cases of over 5 years.[34] A reanalysis of this hypothetical model identified issues with the analysis and its framing, and reported results consistent with the prior literature after correcting for some errors.[36]

For peer review only - http://bmjopen.bmj.com/site/about/guidelines.xhtml

Although the modeling results demonstrated the critical importance of sustaining high population immunity through immunization programs and high-quality surveillance to obtain high confidence about no undetected circulation, the current GPEI strategic plan only covers 2013-2018,[6] which leads to uncertainty about the ability to sustain high program performance after 2018. As of mid-2018, questions continue to arise about when the GPEI will cease to exist and what resources will be available to support the polio endgame, including the certification of eradication of WPV1 and WPV3 with high confidence. The GPEI partners already began transition planning, and this process already led to some downsizing of national poliovirus programs, including the reduction of some AFP surveillance activities.[37] Thus, while the prior modeling assumed strong GPEI and national polio program performance up through the end of the polio endgame, this assumption now appears optimistic, and further analyses that explore the impact of lower quality surveillance may prove useful in the context of global certification decisions for WPV1 and WPV3 eradication. Further motivation for developing models to support certification decisions comes from the re-appearance of WPV1 in security-compromised areas in Borno, Nigeria after apparent interruption, which raised questions about the ability of poliovirus circulation without detection in communities not (or poorly) accessed by immunization and surveillance efforts within larger populations with high immunity and good surveillance.

This study aims to support future decisions about WPV certification by: (1) informing confidence about the absence of circulation by modeling the role of hard-to-reach populations, (2) examining the minimum population size required to sustain poliovirus transmission, and (3) developing a conceptual framework to provide some structure for future certification decisions,.

Methods

To inform confidence about the absence of circulation by modeling the role of hard-to-reach populations, we explored the impact of key assumptions using an existing model of a hypothetical population comprised of a well-vaccinated general population and an under-vaccinated subpopulation.[38] Table 1 lists the model inputs used to characterize this hypothetical population and explore the role of key assumptions (see appendix text, Table A1, and Figures

For peer review only - http://bmjopen.bmj.com/site/about/guidelines.xhtml

A1, A2, and A3 for model details). To explore different population characteristics, we varied the total population size, the size of the under-vaccinated subpopulation, and the degree of mixing between the under-vaccinated and general population around a base case indicated by the bold values in Table 1. In addition, for each variation around the base case, we simultaneously varied the routine immunization coverage and detection probability per polio case in the under-vaccinated subpopulation. We interpret the total hypothetical population as one epidemiological block (e.g., a country) and therefore compute the confidence about no circulation based on all detections that occur in the general population and under-vaccinated subpopulation combined. However, we fix the detection probability in the general population at 95% to characterize high-quality national surveillance while considering lower detection probabilities only in the under-vaccinated subpopulation (Table 1).[38] To estimate the confidence about no circulation in this conceptual model, we use a simplified version (see appendix) of the stochastic approach developed by Eichner and Dietz (1996) [20] and adopted by others.[25-27] We define the probability of undetected circulation after a given period of *t* months without a detection as the number of times in multiple stochastic simulations that *t* months went by without a detection despite continued circulation, divided by the total number of times that *t* months went by without a detection (i.e., with or without continued circulation). Intuitively, the fraction of all time periods of *t* months without a detection but with transmission still ongoing should decrease as *t* increases, corresponding to an increasing probability of no circulation. Confidence about no circulation equals one minus the probability of undetected circulation. To visualize the impact of varying the model inputs, we focus on the time without a detection until the confidence about no circulation first exceeds 95% (CNC95%).

We revisit the question of silent transmission in small populations [22 34 36] using the hypothetical population model [38] in an attempt to inform the bounds on the maximum possible CNC95%. To do so, we ignore the general population and effectively assume a completely-isolated and unvaccinated subpopulation and otherwise adopt the hypothetical population assumptions from Table 1. We transform the DEB model to a stochastic form using the Gillespie algorithm,[39] as described elsewhere, [27] and start either at the endemic equilibrium [34] or with a single infection in an entirely susceptible population. Instead of modeling die-out using the transmission threshold,[2 27] we allow transmission to continue until the infection prevalence becomes 0. This

For peer review only - http://bmjopen.bmj.com/site/about/guidelines.xhtml

complements the existing work [22,34,36] by providing a comparison to the same situation with a more comprehensive model for poliovirus transmission,[2] adding consideration of the impact of the initial conditions, and adding the impact on confidence about no circulation.

Finally, recognizing the complexity and inter-related nature of certification decisions, we developed an influence diagram to relate certification timing decisions to outcomes. The diagram provides a conceptual framework to support certification decisions and formulate decisions about the timing of certification as an optimization problem. The diagram uses conventions from causal loop diagrams [40] and specifies the directionality of relationships between variables using unidirectional arrows. The polarity or sign at the arrow head indicates whether increasing the variable at the base of the arrow increases (+) or decreases (-) the variable that the arrow points to with all else being equal.

Patient and Public Involvement

This study did not involve patients or public opportunities for engagement.

Results

Figure 1 illustrates how the confidence about no circulation increases with time after the last detection as a function of the surveillance quality in the under-vaccinated subpopulation (i.e., the detection probability). Clearly, higher confidence implies the need to wait longer after the last detected case, and lower detection probabilities further increase the time required to reach a certain level of confidence (e.g., the 95% line). Figure 1 shows a relatively modest effect of the detection probability in the under-vaccinated subpopulation for this hypothetical model due to continued occurrence of cases in the general population for the assumed degree of mixing (see appendix).

Figure 2 shows the CNC95% values as a function of coverage and detection probability for the under-vaccinated subpopulation. The figure shows longer times required to reach CNC95% values with increasingly more isolated under-vaccinated subpopulations (left column, top to

For peer review only

For peer review only - http://bmjopen.bmj.com/site/about/guidelines.xhtml

bottom), with decreasing relative sizes of the under-vaccinated subpopulation (middle column, top to bottom), and decreasing absolute sizes of a fully-isolated under-vaccinated subpopulation (right column, top to bottom, note increased y-axis ranges). The panels in Figure 2 omit curves for coverage values that do not result in eradication, because they do not allow for calculation of any confidence about eradication. The panels also omit the data point for 0 detection probability in the event of a fully-isolated under-vaccinated subpopulation, because that would imply no ability to detect the virus. Consistent with previous findings,[27] all panels in Figure 2 show higher CNC95% values with higher coverage in the under-vaccinated subpopulation. In each panel, the lowest shown coverage value may result in the longest period of undetected circulation before interruption and therefore result in the longest time to achieve high confidence about no circulation.

Looking more closely at the differences between the columns, the left column of Figure 2 shows a very strong influence of the degree of isolation on the CNC95%. With little isolation and no surveillance in the under-vaccinated subpopulation, the general population with high surveillance quality can still detect transmission because of relatively frequent spillover of polio cases (see appendix). Thus, the results do not depend much on the detection probability in the under-vaccinated subpopulation for p_{within}=0.8. In contrast, for a fully isolated under-vaccinated subpopulation (p_{within}=1), the detection probability in this population becomes a more important driver of the CNC95% than the coverage (i.e., for detection probability of 0.1 or very poor surveillance and all other inputs at the base case, the CNC95% equals almost 6 years regardless of coverage). The middle column of Figure 2 shows CNC95% values of approximately 5 years with no surveillance in a relatively small under-vaccinated subpopulation. Although the relative size of the under-vaccinated subpopulation affects the mixing dynamics and incidence of cases in both populations, much of the observed effect comes from the implied change in the absolute size of the under-vaccinated subpopulation, which directly affects the typical time between cases. As shown in the right column of Figure 2, changing the absolute size of the under-vaccinated subpopulation in the event of full isolation from the general population and a detection probability of 0.1 dramatically affects the CNC95%, which ranges from slightly over 2 years for 500,000 people to approximately 9 years for 50,000 people (i.e., a 4-fold increase in CNC95% for a 10-fold increase in population size).

For peer review only - http://bmjopen.bmj.com/site/about/guidelines.xhtml

Considering the relatively high CNC95% observed for small, isolated populations in Figure 2, Figure 3A uses a stochastic model to show the distribution of the duration of circulation in a single population not reached by vaccination at all. Figure 3A shows the results as a function of population size for a model initialized at the endemic equilibrium. For very small population sizes (e.g., hundreds), not surprisingly poliovirus infections typically die-out within a year, with a maximum duration of circulation of one year and 4 months for a closed population of 1,000 people (based on 10,000 iterations). The maximum duration of circulation increases rapidly for larger populations. For a population of 5,000 people, circulation continues for 3 or more years in 50 of 10,000 (0.5%) iterations. With population sizes of 10,000, 20,000, 30,000, 40,000 and 50,000, circulation continues for at least 10 years for 3%, 34%, 63%, 79%, and 88% of iterations, respectively.

Figure 3B shows the same analysis as Figure 3A except that it changes the initial conditions by assuming a population with no prior exposure to any polioviruses. In this context, a single introduction rapidly burns through the entire susceptible population and quickly exhausts susceptible individuals, leading to die-out and a maximum duration of circulation of less than 2 years for all population sizes considered in Figure 3b. Together, Figures 3A and 3B encompass the bounds on the possible duration of circulation for different initial conditions. In reality, small, completely isolated populations are unlikely to remain at the endemic equilibrium because of random fluctuations in the incidence, seasonality, and die-out, and no completely naïve populations likely exist. In a separate analysis using the same model, we verified that the addition of seasonality decreases the typical duration of circulation and increases the probability of eradication within 5 years. For example, for a population size of 20,000 people, the probability of eradication within 5 years increased from approximately 64% without seasonality to 78%-92% with a seasonal amplitude of 10% (applied to the basic reproduction number of 10), depending on the timing of the seasonal peak.

While Figure 3 implies that increasing the population size results in an increasing probability of persistent circulation (i.e., a greater probability of sustained undetected transmission), Figure 2 implies that increasing population size decreases the typical time interval between cases (i.e.,

For peer review only - http://bmjopen.bmj.com/site/about/guidelines.xhtml

lower probabilities of sustained undetected circulation). Figure 4 shows the net effect of these two opposing trends and suggests that an optimal population size exists around 20,000 people. For smaller population sizes, continued transmission becomes exceedingly unlikely (Figure 3), while for larger population sizes, undetected circulation becomes less likely due to the higher frequency of cases. This non-linear behavior suggests a maximum CNC95% of approximately 2.5 years for a detection probability of 1, although the maximum increases to up to 9 years for a very low detection probability of 0.1 and a population size of 20,000 to 30,000 people.

Figure 5 shows how the desired confidence about no circulation may influence certification timing and key health economic outcomes (see appendix text and Table A2 for details). Earlier certification and OPV cessation may increase the risk of undetected circulation after OPV cessation (and therefore the possibility of needing to restart OPV use) but may decrease the costs until OPV cessation (and therefore the overall global costs for planned polio immunization). Therefore, the fundamental optimization problem consists of finding the desired confidence about no WPV circulation at OPV cessation that minimizes the resulting total financial and societal costs. Figure 5 also shows that the costs and risks both depend on the GPEI budget until and after OPV cessation, with a lower budget saving costs in the short term but increasing the time of OPV cessation at a given confidence level and the risks of OPV restarts, which may ultimately result in greater overall costs. Optimization of the desired confidence about no WPV circulation depends critically on how the confidence about no circulation increases with time after the last detected event from the surveillance system.

Discussion

Hard-to-reach subpopulations may play a key role in deliberations about WPV circulation and decisions about WPV certification. The timing of WPV certification and subsequent OPV cessation involves high stakes and largely depends on the desired confidence about the absence of circulation. Surveillance quality emerges as a key factor that affects both the confidence about the absence of circulation and the ability to detect and control any outbreaks after OPV cessation. However, national surveillance indicators may not suffice to measure the overall surveillance system quality because gaps in surveillance at the level of tens of thousands of

For peer review only

people may influence confidence. Our modeling suggests that high quality surveillance suffices to detect transmission in the context of a relatively well-mixed under-vaccinated subpopulation (e.g., in Pakistan and Afghanistan),[41] while local gaps may miss transmission for several years in the context of highly-isolated under-vaccinated subpopulations. With respect to global certification of WPV eradication, this implies a need to address any such gaps in isolated populations that experienced WPV transmission during the last decade. The recent experience in Borno and previously in Chad and Sudan demonstrated the ability of WPVs to circulate undetected for many years in sub-populations missed by both surveillance and immunization efforts.[12 13] However, one of the main contributions of this work is that is shows that very small, isolated subpopulations cannot sustain transmission indigenously, while in the context of even very limited surveillance, persistent undetected transmission becomes increasingly unlikely for increasing population sizes. To our knowledge, the existence of a worst-case population size for undetected circulation has not yet been demonstrated for polioviruses. Our analysis confirms that with high-quality surveillance, 3 years without a detected WPV case suffices to attain high confidence about no circulation for serotype 1, even considering possible persistence in very small population sizes.

Explicit consideration of the decision to certify WPV eradication (Figure 5) suggests that if we remain confident that we can prevent the need to restart OPV due to uncontrolled outbreaks resulting from a possible WPV reemergence, then we should accept a lower confidence about the absence of circulation to certify sooner, because the costs of delaying OPV cessation would outweigh the risk of premature certification. Earlier OPV cessation particularly represents the best option if diminishing GPEI financial and/or global OPV supply resources limit our ability to maintain population immunity and/or respond effectively to post-cessation outbreaks. However, this choice depends on a willingness to accept the reputational risk of finding out that WPV still circulates despite its certification. With WPV3 not detected anywhere since 2012 [11] and in many places for decades, the confidence about no WPV3 circulation continues to grow. Although confidence about no circulation increases more slowly for WPV3 than WPV1 due to the lower PIR,[25 27] assuming 1-2 years to prepare for coordinated global OPV cessation, starting the process of removing serotype 3 OPV now would imply at least 7 years of no detection since the last WPV3 case and synchronized cessation of serotype 3 OPV use (i.e., 2012 to 2019-2020).

For peer review only - http://bmjopen.bmj.com/site/about/guidelines.xhtml

The transition of GPEI resources already occurring leads to expected decreases in population immunity for serotype 3 in some areas. Combined with on-going serotype 3 vaccine-associated paralytic poliomyelitis, this should motivate careful consideration of the costs, benefits, risks, and logistical challenges of globally certifying WPV3 eradication and synchronizing serotype 3 OPV cessation before completing WPV1 eradication and serotype 1 OPV cessation, which now appears at least 4 years away.

Our results related to minimum population sizes appear consistent with a prior study [22] that found an average of approximately 5 years of circulation for a population of 20,000 people in a high-R_0 setting and an exponential increase in the average duration of circulation with increasing population size. The prior study also reported a higher probability of virus persistence as the degree of mixing between subpopulations increases.[22] Our study suggests that more mixing between subpopulations may not lead to a higher probability of undetected circulation because surveillance can more easily detect persistent viruses for higher degrees of mixing. Using a more realistic model than another prior analysis,[36] we similarly do not find a high probability of persistent transmission for populations of 10,000 people or less.

Like all models, our model makes simplifying assumptions that affect its behavior.[2] Specifically, we characterized a stylized, hypothetical population to systematically explore key assumptions, used a simplified semi-stochastic approach to compute CNC95% that does not fully account for all stochastic variability, and deterministically characterized die-out. However, for the analysis of small population sizes that depend most on stochastic variability, we accounted for stochastic variability and die-out at the individual level.

While this study highlights the importance of ensuring high surveillance quality in all subpopulations, it also reiterates the role of immunization in accelerating confidence about no circulation after the last detection.[27] Achieving and maintaining high population immunity to transmission represents a mission critical component of the GPEI.[4] Populations with immunity near the threshold experience increased risk of prolonged undetected transmission. Failing to invest relatively small amounts of resources to maintain high population immunity can lead to much more costly outbreaks, as occurred for example in Tajikistan.[3] Thus, if ensuring high-

For peer review only - http://bmjopen.bmj.com/site/about/guidelines.xhtml

quality surveillance in all subpopulations remains an elusive goal, then achieving better coverage in those subpopulations would still result in higher confidence about no circulation. In contrast, high quality surveillance in the context of poor immunization still leaves the population and the world at risk.

Poliovirus environmental surveillance can detect polioviruses even in the absence of symptomatic polio cases [42,43] and offers the potential to fill some local gaps in symptomatic poliovirus surveillance. For example, the extensive environmental surveillance system in Israel effectively detected transmission of circulating WPV1 in the absence of any cases and despite very high coverage with inactivated poliovirus vaccine (IPV).[31,44] However, despite the potential for high sensitivity of environmental surveillance to detect infected individuals excreting into the catchment area, its sensitivity remains zero outside of the catchment area and depends on sampling frequency (e.g., one sample every year provides little increase in confidence over AFP alone and the quality matters).[45] Environmental surveillance system designs generally depend on access to a centralized sewage network,[43] which hard-to-reach subpopulations (i.e., those most likely to sustain undetected poliovirus transmission) may not possess. Further research should help to explore the ability of environmental surveillance to increase confidence about no circulation in specific areas, and the value of the information obtained from environmental surveillance relative to its costs requires evaluation.

IPV plays a relatively limited role with respect to the CNC. While IPV protects otherwise susceptible individuals from paralysis if they become subsequently infected with a live poliovirus and may reduce the participation of these individuals in transmission to some degree, the decreased frequency of paralysis in live poliovirus-infected individuals in the population may delay the detection of any circulating live poliovirus in countries by AFP surveillance (i.e., less frequent detection of polio AFP cases depending on IPV coverage) We note the polio AFP detection rate depends on the exposure of fully-susceptible individuals to live poliovirus and it differs from the non-polio AFP detection rate, with the Global Polio Laboratory Network uses to monitor performance of the AFP surveillance system and is not affected by IPV use). Overall, immunization with IPV helps to maintain population immunity to transmission somewhat, but given births of immunologically naïve, deaths of immune individuals, waning immunity, and the

For peer review only - http://bmjopen.bmj.com/site/about/guidelines.xhtml

absence of circulating live polioviruses, population immunity to transmission declines following WPV eradication and homotypic OPV cessation, even with very high IPV coverage.[46] The extent of transmission possible following reintroduction of a live poliovirus into a country with high IPV coverage will depend on the relative contributions of fecal-oral and oropharyngeal routes to overall transmission.[4] In countries dominated by fecal-oral transmission, the use of IPV will not prevent or stop transmission, and reintroduced live polioviruses that restart transmission may lead to the need to restart the use of OPV.[47]

List of abbreviations: AFP, acute flaccid paralysis; CNC95%, Time until the confidence about no circulation reaches 95%; cVDPV, circulating VDPV DEB, differential-equation based; GPEI, Global Polio Eradication Initiative; IPV, inactivated poliovirus vaccine; OPV, oral poliovirus vaccine; PIR, paralysis-to-infection ratio; VDPV, vaccine-derived poliovirus; WPV(1,2,3), wild poliovirus (of serotype 1, 2, 3, respectively)

DECLARATIONS

Authors' contributions

All authors (RDT, DAK, KMT) contributed to the study design, model development, interpretation of results, manuscript writing, and revisions. The first and second authors (RDT, DAK) performed the modeling and analyses, and the last author (KMT) secured the funding for the study.

Ethics approval and consent to participate

Not applicable

Consent to publish

Not applicable

Competing interests

None

For peer review only

Funding

This work was funded by the Bill and Melinda Gates Foundation [OPP1129391].

Acknowledgments

We thank the Bill and Melinda Gates Foundation for supporting the completion of this work [OPP1129391].

Data sharing statement

Technical appendix available on request from the authors.

References

1. World Health Assembly. Global eradication of poliomyelitis by the year 2000 (resolution 41.28). Geneva: World Health Organization, 1988.
2. Duintjer Tebbens RJ, Pallansch MA, Kalkowska DA, et al. Characterizing poliovirus transmission and evolution: Insights from modeling experiences with wild and vaccine-related polioviruses. *Risk Analysis* 2013;23(4):703-49.
3. Thompson KM, Pallansch MA, Duintjer Tebbens RJ, et al. Modeling population immunity to support efforts to end the transmission of live polioviruses. *Risk Analysis* 2013;33(4):647-63.
4. Thompson KM, Kalkowska DA, Duintjer Tebbens RJ. Managing population immunity to reduce or eliminate the risks of circulation following the importation of live polioviruses *Vaccine* 2015;33(3):1568-77.
5. Thompson KM, Duintjer Tebbens RJ. The case for cooperation in managing and maintaining the end of poliomyelitis: Stockpile needs and coordinated OPV cessation. *The Medscape Journal of Medicine* 2008;10(8):190.
6. World Health Organization. Global Polio Eradication Initiative: Polio Eradication and Endgame Strategic Plan (2013-2018). Geneva, 2013.
7. World Health Assembly. Poliomyelitis: mechanism for management of potential risks to eradication (resolution 61.1). Geneva: World Health Organization, 2008.
8. Global Polio Eradication Initiative. Global eradication of wild poliovirus type 2 declared 2015 [updated September 20, 2015. Available from: http://www.polioeradication.org/mediaroom/newsstories/Global-eradication-of-wild-poliovirus-type-2-declared/tabid/526/news/1289/Default.aspx accessed November 30 2015.
9. Hampton LM, Farrell M, Ramirez-Gonzalez A, et al. Cessation of trivalent oral poliovirus vaccine and introduction of inactivated poliovirus vaccine - Worldwide, 2016. *Morbidity and Mortality Weekly Report* 2016;65(35):934-38. doi: 10.15585/mmwr.mm6535a3
10. World Health Organization. Polio this week as of 3 January 2018 2018 [Available from: http://polioeradication.org/polio-today/polio-now/this-week/ accessed January 10 2018.

For peer review only - http://bmjopen.bmj.com/site/about/guidelines.xhtml

11. Kew OM, Cochi SL, Jafari HS, et al. Possible eradication of wild poliovirus type 3--worldwide, 2012. *Morbidity and Mortality Weekly Report* 2014;63(45):1031-33.

12. World Health Organization. Laboratory surveillance for wild and vaccine-derived polioviruses, January 2004-June 2005. *Weekly Epidemiological Record* 2005;80(39):335-40.

13. Nnadi C, Damisa E, Esapa L, et al. Continued endemic wild poliovirus transmission in security-compromised areas - Nigeria, 2016. *MMWR Morb Mortal Wkly Rep* 2017;66(7):190-93. doi: 10.15585/mmwr.mm6607a2

14. Nathanson N, Kew OM. From emergence to eradication: the epidemiology of poliomyelitis deconstructed. *American Journal of Epidemiology* 2010;172(11):1213-29. [published Online First: 2010/10/28]

15. World Health Organization. Transmission of wild poliovirus type 2 - Apparent global interruption. *Weekly Epidemiological Record* 2001;76:95-97.

16. Deshpande JM, Nadkarni SS, Siddiqui ZA. Detection of MEF-1 laboratory reference strain of poliovirus type 2 in children with poliomyelitis in India in 2002 & 2003. *Indian Journal of Medical Research* 2003;118:217-23.

17. Duizer E, Ruijs WL, van der Weijden CP, et al. Response to a wild poliovirus type 2 (WPV2)-shedding event following accidental exposure to WPV2, the Netherlands, April 2017. *Euro Surveillance* 2017;22(21):pii=30542. doi: 10.2807/1560-7917.ES.2017.22.21.30542

18. Centers for Disease Control and Prevention. Certification of poliomyelitis eradication--the Americas, 1994. *MMWR Morb Mortal Wkly Rep* 1994;43(39):720-2.

19. Debanne SM, Rowland DY. Statistical certification of eradication of poliomyelitis in the Americas. *Mathematical Biosciences* 1998;150(1):83-103.

20. Eichner M, Dietz K. Eradication of poliomyelitis: when can one be sure that polio virus transmission has been terminated? *American Journal of Epidemiology* 1996;143(8):816-22.

21. Eichner M, Hadeler KP. Deterministic models for the eradication of poliomyelitis: vaccination with the inactivated (IPV) and attenuated (OPV) polio virus vaccine. *Mathematical Biosciences* 1995;127(2):149-66.

22. Eichner M, Hadeler KP, Dietz K. Stochastic models for the eradication of poliomyelitis: minimum population size for polio virus persistence. In: Isham V, Medley GF, eds. Models for Infectious Human Diseases: their structure and relation to data. New York: Cambridge University Press 1996:315-27.

23. World Health Organization. Global Polio Eradication Initiative: Strategic plan 2004-2008. Geneva, 2004.

24. Bahl S, Kumar R, Menabde N, et al. Polio-free certification and lessons learned - South-East Asia region, march 2014. *Morbidity and Mortality Weekly Report* 2014;63(42):941-46.

25. Kalkowska DA, Duintjer Tebbens RJ, Thompson KM. The probability of undetected wild poliovirus circulation after apparent global interruption of transmission. *American Journal of Epidemiology* 2012;175(9):936-49.

26. Houy N. The probability of undetected wild poliovirus circulation: Can we do better? *Journal of Theoretical Biology* 2015;382:272-78.

27. Kalkowska DA, Duintjer Tebbens RJ, Pallansch MA, et al. Modeling undetected live poliovirus circulation after apparent interruption of transmission: Implications for

For peer review only - http://bmjopen.bmj.com/site/about/guidelines.xhtml

surveillance and vaccination. *BMC infectious diseases* 2015;15(66):1. doi: 10.1186/s12879-015-0791-5
28. Kalkowska DA, Duintjer Tebbens RJ, Thompson KM. Modeling strategies to increase population immunity and prevent poliovirus transmission in two high-risk areas in northern India. *Journal of Infectious Diseases* 2014;210(Suppl 1):S398-S411.
29. Kalkowska DA, Duintjer Tebbens RJ, Thompson KM. Modeling strategies to increase population immunity and prevent poliovirus transmission in the high-risk area of northwest Nigeria. *Journal of Infectious Diseases* 2014;210(Suppl 1):S412-S23.
30. Duintjer Tebbens RJ, Kalkowska DA, Wassilak SGF, et al. The potential impact of expanding target age groups for polio immunization campaigns. *BMC infectious diseases* 2014;14:45.
31. Kalkowska DA, Duintjer Tebbens RJ, Grotto I, et al. Modeling options to manage type 1 wild poliovirus imported into Israel in 2013. *Journal of Infectious Diseases* 2015;211(11):1800-12.
32. Berchenko Y, Manor Y, Freedman LS, et al. Estimation of polio infection prevalence from environmental surveillance data. *Sci Transl Med* 2017;9(383) doi: 10.1126/scitranslmed.aaf6786
33. Famulare M. Has wild poliovirus been eliminated from Nigeria? . *PLoS One* 2016;10:e0135765.
34. Vallejo C, Keesling J, Koopman JS, et al. Silent circulation of poliovirus in small populations. *Infectious Disease Modelling* 2017;2(4):431-40. doi: 10.1016/j.idm.2017.11.001
35. Vallejo C, Keesling J, Koopman JS, et al. Corrigendum to "Silent circulation of poliovirus in small populations". *Infectious Disease Modelling* 2018;3:136-38. doi: 10.1016/j.idm.2017.11.001
36. Kalkowska DA, Duintjer Tebbens RJ, Thompson KM. Another look at silent circulation of poliovirus in small populations. *Infectious Disease Modelling* 2018;3:107-17. doi: https://doi.org/10.1016/j.idm.2018.06.001
37. World Health Organization. Report on polio transition planning - advance draft as of 18 April, 2017 2017 [Available from: http://www.who.int/mediacentre/events/2017/advance-report-polio-transmission.pdf?ua=1 accessed March 12 2018.
38. Thompson KM, Duintjer Tebbens RJ. Lessons from the polio endgame: Overcoming the failure to vaccinate and the role of subpopulations in maintaining transmission. *Journal of Infectious Diseases* 2017;216(Suppl 1):S176-S82.
39. Gillespie DT. A general method for numerically simulating the stochastic time evolution of coupled chemical reactions. *Journal of Computational Physics* 1976;22:403-34.
40. Sterman J. Business dynamics: Systems thinking and modeling for a complex world. Boston, MA: McGraw-Hill 2000.
41. Duintjer Tebbens RJ, Pallansch MA, Cochi SL, et al. Modeling poliovirus transmission in Pakistan and Afghanistan to inform vaccination strategies in undervaccinated subpopulations. *Risk Analysis* 2018;38(8):1701-17. doi: 10.1111/risa.12962
42. Global Polio Eradication Initiative. Guidelines on environmental surveillance for detection of polioviruses - working draft - March 2015, 2015.
43. Duintjer Tebbens RJ, Zimmermann M, Pallansch MA, et al. Insights from a systematic search for information on designs, costs, and effectiveness of poliovirus environmental

For peer review only - http://bmjopen.bmj.com/site/about/guidelines.xhtml

surveillance systems. *Food and Environmental Virology* 2017;9(4):361-82. doi: 10.1007/s12560-017-9314-4
44. Anis E, Kopel E, Singer S, et al. Insidious reintroduction of wild poliovirus into Israel, 2013. *Euro Surveillance* 2013;18(38):pii=20586.
45. Kalkowska DA, Duintjer Tebbens RJ, Thompson KM. Environmental surveillance system characteristics and impacts on confidence about no undetected serotype 1 wild poliovirus circulation. *Risk Analysis* 2018:on-line September 21, 2018, doi: 10.1111/risa.13193.
46. Thompson KM, Wallace GS, Duintjer Tebbens RJ, et al. Trends in the risk of U.S. polio outbreaks and poliovirus vaccine availability for response. *Public Health Reports* 2012;127(1):23-37.
47. Duintjer Tebbens RJ, Thompson KM. Polio endgame risks and the possibility of restarting the use of oral poliovirus vaccine. *Expert Review of Vaccines* 2018;17(8):739-51. doi: 10.1080/14760584.2018.1506333

For peer review only - http://bmjopen.bmj.com/site/about/guidelines.xhtml

Table 1: Model inputs to characterize a hypothetical population that contains an under-vaccinated subpopulation.

**Model input**	**Value(s)[a]**	**Source/notes**
Total population size	500,000;**1 million**;5 million	No effect on DEB model behavior, but required for stochastic analysis of detections
Time until vaccination starts, years<br>General population<br>Under-vaccinated subpopulation	 30<br>40	Assumption to characterize hard-to-reach subpopulation within well-vaccinated general population
Initial age distribution<br>0-2 months<br>3-59 months<br>5-14 years<br>≥ 15 years	 0.01<br>0.15<br>0.25<br>0.59	Equilibrium age distribution[38]
Birth rate, births/person/year	0.02	[38]
Death rate, deaths/person/year	0.02	[38]
Basic reproduction number (R_0)	10	[38]
Proportion of transmissions via oropharyngeal route	0.3	[38]
Proportion of contacts reserved for individuals within the same mixing age group	0.4	Same value as used in [38] (not explicitly listed)
Average per-dose take rate for serotype 1 OPV	0.6	Increased from 0.5 to maintain similar coverage thresholds with different run-up [38]
Routine immunization coverage<br>General population<br>Under-vaccinated subpopulation	 0.95<br>0.75;0.82;0.85;0.90;0.95[b]	Represents coverage with exactly 3 OPV doses; general population based on [38], under-vaccinated varied around threshold to eradicate, which equals 0.82 for the bolded values in the middle column
Proportion of contacts with under-vaccinated subpopulation (p_{within})	0.8;**0.95**;1.00	Selected values from [38]
Size of under-vaccinated subpopulation compared to total population	1/20;**1/10**;1/5	Selected values from [38]
Paralysis-to-infection ratio (PIR)	1/200	Average for serotype 1 wild poliovirus[2,14]
Detection probability per polio case<br>General population<br>Under-vaccinated subpopulations	 0.95<br>0;0.1;0.2;0.3;0.4;0.5;0.6;0.7;0.8;0.9;0.95[b]	Assumption to characterize hard-to-reach subpopulation within general population with high acute flaccid paralysis surveillance quality

Abbreviations: DEB, differential-equation based; OPV, oral poliovirus vaccine

[a] Values shown in bold represent values that we held fixed when varying other values in sensitivity analyses

[b] All values considered jointly in all sensitivity analysis (hence no single value bolded)

For peer review only - http://bmjopen.bmj.com/site/about/guidelines.xhtml

For peer review only

Figure Captions

Figure 1: Confidence about no circulation as a function of time since the last detection for different detection probability values for the hypothetical model base case, with coverage at the corresponding minimum to eliminate WPV (i.e., 0.82).

Figure 2: Time until the confidence about no circulation reaches 95% (CNC95%) from the stochastic analysis for different degrees of isolation of the under-vaccinated subpopulation (left column), relative sizes of the under-vaccinated subpopulation (middle column), and absolute sizes of a fully-isolated under-vaccinated subpopulation (right column, note doubled y-axis ranges).

Figure 3: Results from the analysis of the relationship between population size and persistence of circulation of serotype 1 wild poliovirus transmission in the fully stochastic model when (A) the model starts at the endemic equilibrium and (B) the model starts with a single infection in a fully susceptible population

Figure 4: Time until the confidence about no circulation reaches 95% (CNC95%) for small population sizes in the fully stochastic model that starts at the endemic equilibrium, as a function of detection probability (DP)

Figure 5: Conceptual diagram for the implications of choices about the timing of certification of eradication of a wild poliovirus serotype on total financial and societal costs

For peer review only

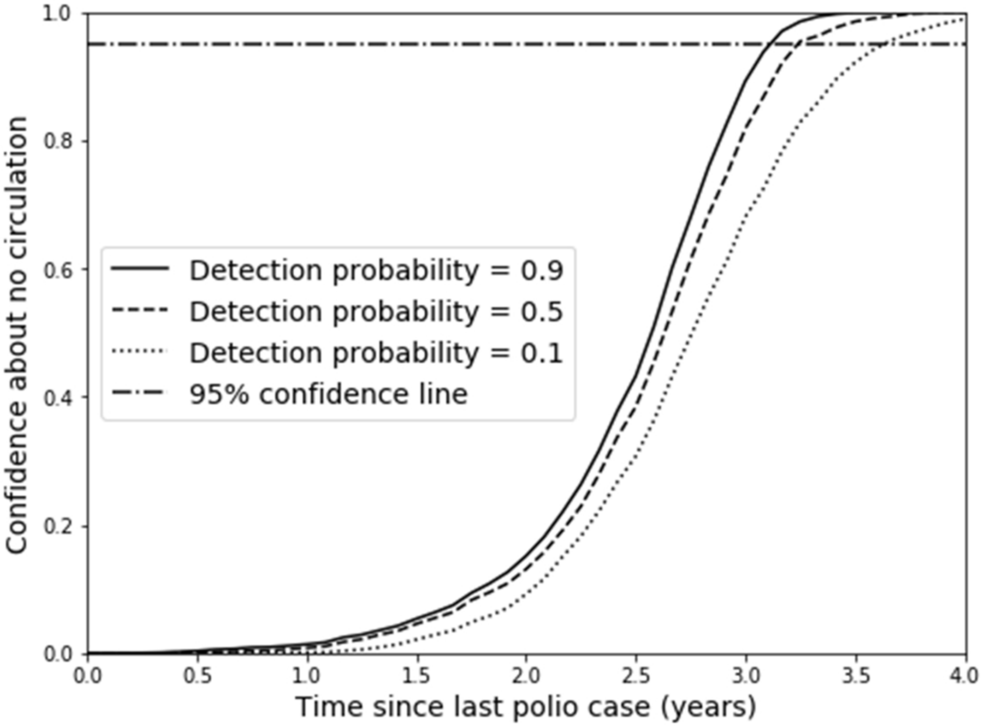

Figure 1: Confidence about no circulation as a function of time since the last detection for different detection probability values for the hypothetical model base case, with coverage at the corresponding minimum to eliminate WPV (i.e., 0.82).

179x133mm (300 x 300 DPI)

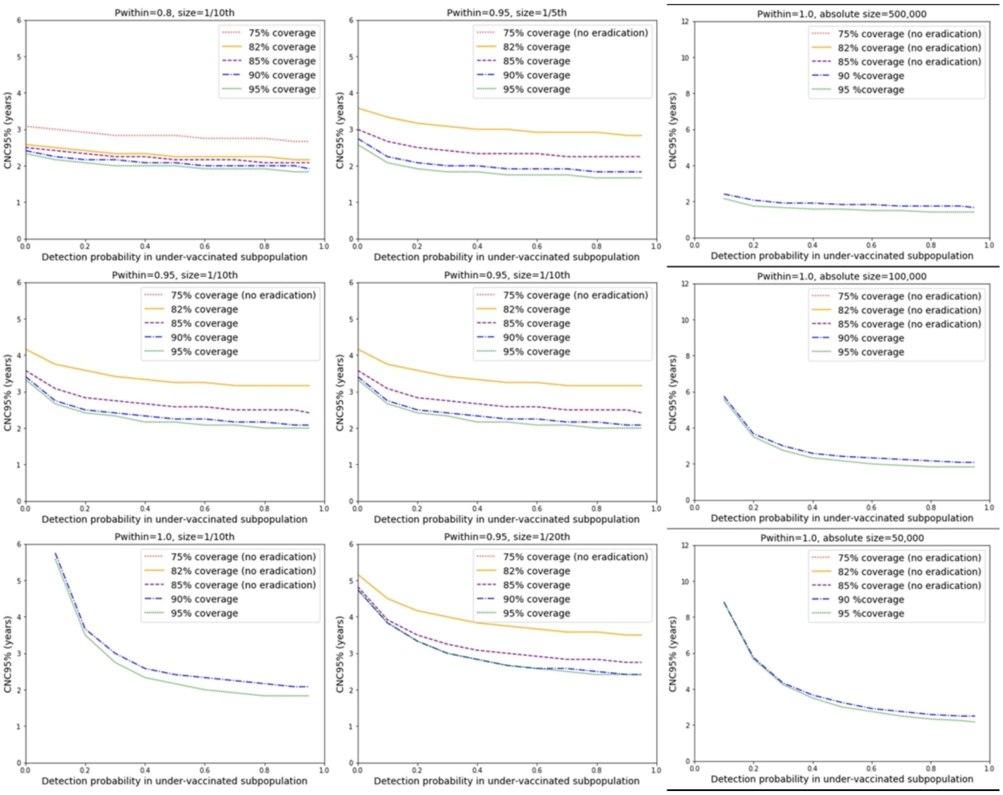

Figure 2: Time until the confidence about no circulation reaches 95% (CNC95%) from the stochastic analysis for different degrees of isolation of the under-vaccinated subpopulation (left column), relative sizes of the under-vaccinated subpopulation (middle column), and absolute sizes of a fully-isolated under-vaccinated subpopulation (right column, note doubled y-axis ranges).

250x196mm (300 x 300 DPI)

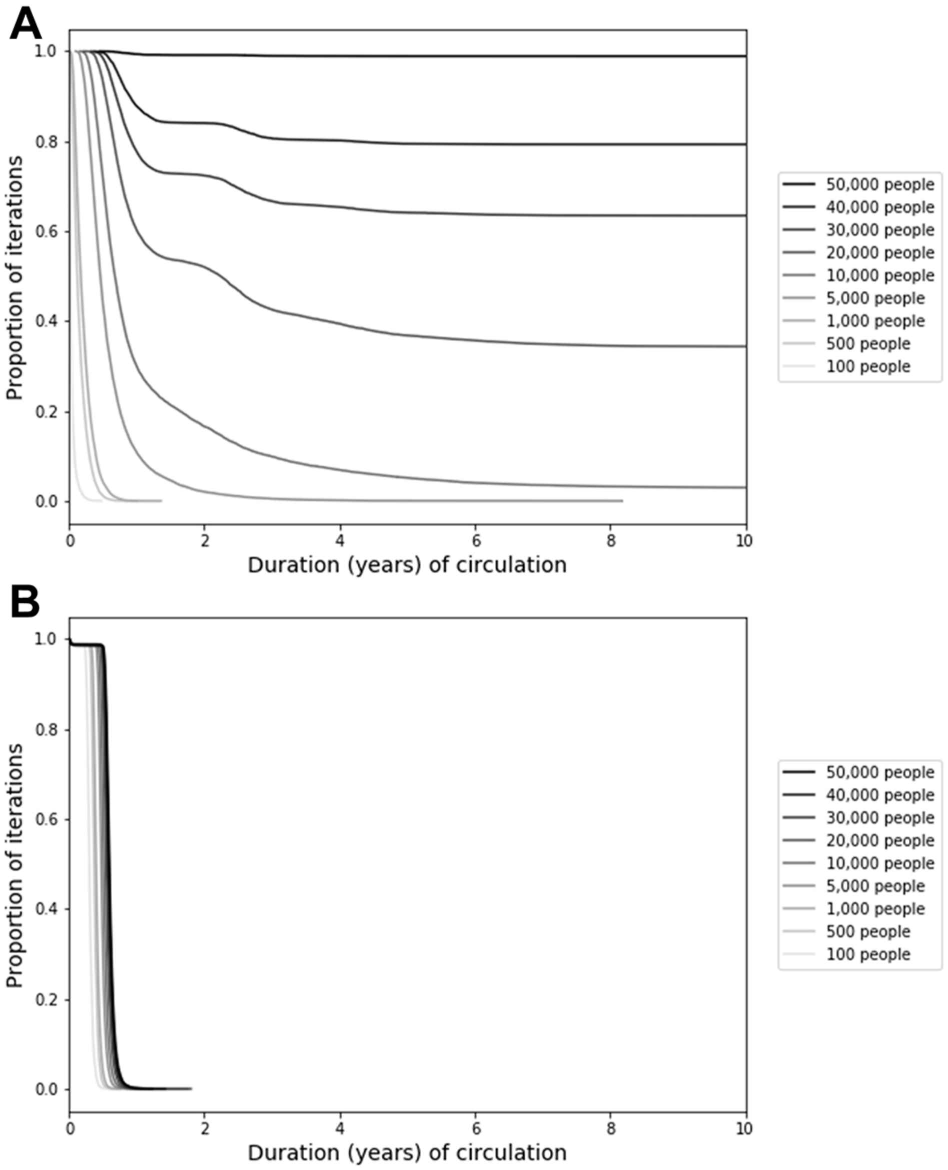

Figure 3: Results from the analysis of the relationship between population size and persistence of circulation of serotype 1 wild poliovirus transmission in the fully stochastic model when (A) the model starts at the endemic equilibrium and (B) the model starts with a single infection in a fully susceptible population

90x113mm (300 x 300 DPI)

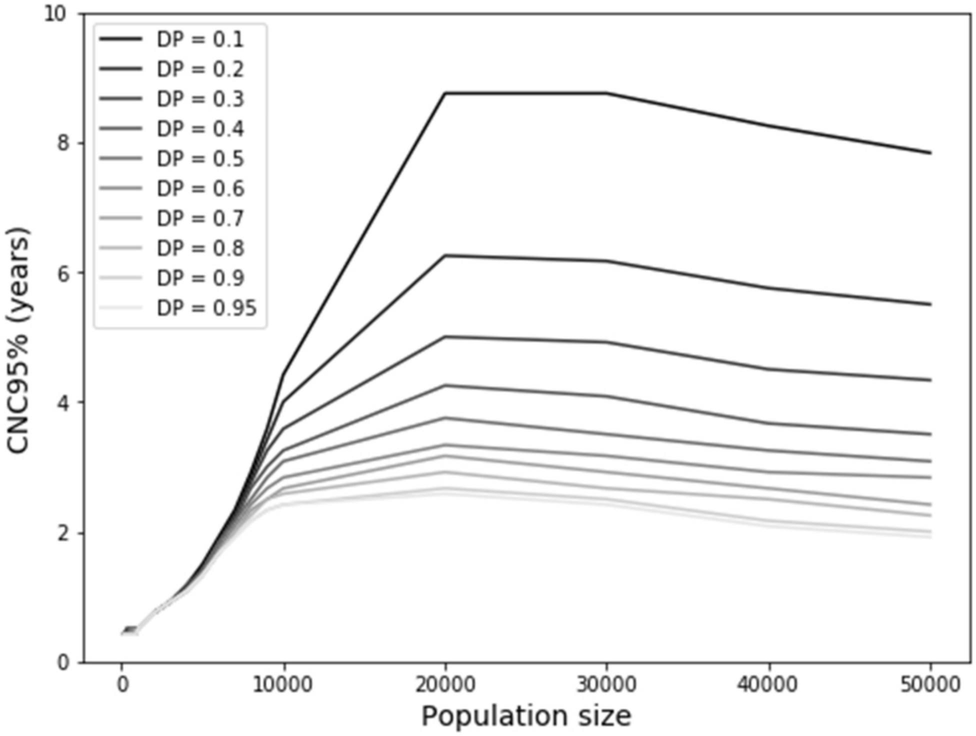

Figure 4: Time until the confidence about no circulation reaches 95% (CNC95%) for small population sizes in the fully stochastic model that starts at the endemic equilibrium, by detection probability (DP)

177x133mm (300 x 300 DPI)

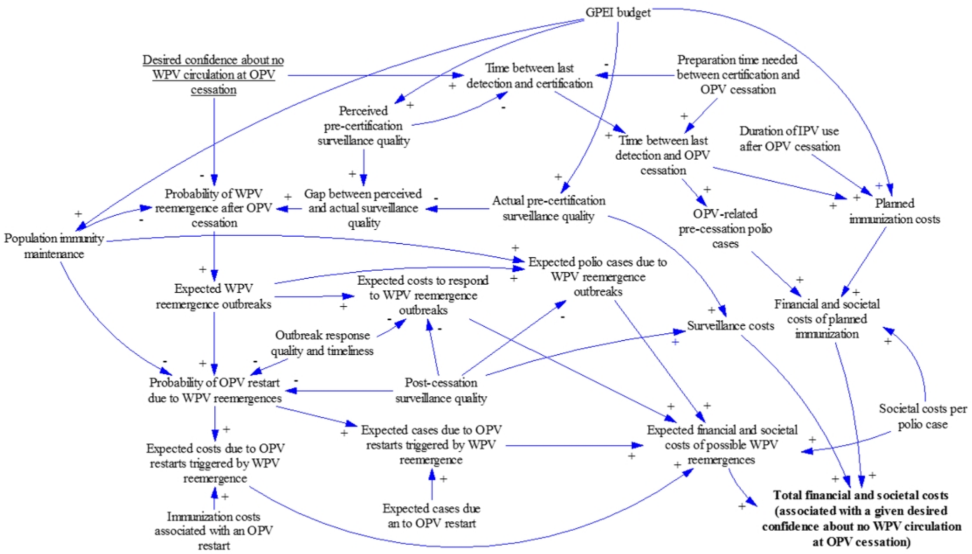

Figure 5: Conceptual diagram for the implications of choices about the timing of certification of eradication of a wild poliovirus serotype on total financial and societal costs

228x135mm (300 x 300 DPI)

APPENDIX for "Certification of global eradication: The role of hard-to-reach subpopulations and confidence about the absence of transmission"

Radboud J. Duintjer Tebbens,[1] Dominika A. Kalkowsa,[1] Kimberly M. Thompson[1]

Differential-equation based model and results

The DEB model we use to examine the role of subpopulations [38] made simplifying assumptions about what a high-risk population might look like and otherwise adopted the comprehensive structure and setting-invariant model inputs of a previously developed and calibrated differential-equation based poliovirus transmission and OPV evolution model.[2 30] The following text from the appendix of a prior publication [45] (with references renumbered) briefly describes the model and Figures A1-2 and Table A1 cited in the text provide the model structure and generic inputs (i.e., model inputs that remain the same for all populations).

"The differential equation-based poliovirus transmission and OPV evolution model (DEB model) [2] tracks the movement of people between demographic age groups (grouped into mixing age groups that mix preferentially amongst themselves), and for each serotype between oropharyngeal and intestinal infection stages (resulting in potential oropharyngeal and fecal-oral transmission, respectively), immunity states, and waning stages. Figure A1 provides an overview of the model structure based on prior work.[2] Figure A1a depicts the immunity states with the flows that move individuals in and out of them and Figure A1b details how effectively vaccinated or infected individuals progress through different stages of infection and, in the event of infection with OPV, through OPV evolution stages. The model assumes that active immunity from prior vaccination or infection results in permanent protection from polio (disease), but only partial protection from subsequent infection and participation in transmission, depending on the nature of immunity (IPV-induced vs. LPV-induced or both) and time since the last exposure (i.e. waning stage). The model includes 5 waning stages, 6 fecal-oral and 6 oropharyngeal infection stages (2 latent and 4 infectious, with varying degrees of infectiousness), and also accounts for a delay between IPV receipt and development of the immune response that moves individuals to the next IPV immunity state. In Figure A1a, we note that the model assumes identical properties for "IPV and LPV" and "≥ 2 LPV infections" and that the recent waning stages of these immunity states represent the highest degree of immunity to transmission in the model. The model further tracks OPV evolution by moving individuals infected with the OPV parent strain (stage 0) through 20 successive reversion stages that can each transmit and that come with increasing paralysis-to-infection ratios and relative basic reproduction numbers (R_0 values) compared to homotypic WPVs. The last reversion stage (stage 19) represents fully-reverted VDPVs with assumed paralysis-to-infection ratio and R_0 equivalent to homotypic WPVs. For WPVs or any OPV reversion stage, the DEB model mimics die-out by setting the force-of-infection for the given strain to 0 whenever its effective prevalence of infections resides below a calibrated threshold of 5 per million people. Consequently, OPV-related viruses can only continue to transmit and thus evolve to cVDPVs through successive infections when low enough population immunity to transmission permits circulation of the OPV viruses introduced in the population through vaccination. We fixed the die-out process, model structure, and numerical

For peer review only - http://bmjopen.bmj.com/site/about/guidelines.xhtml

model inputs that characterize them across all populations we modeled and Table A1 includes the corresponding generic model inputs. […]

"Figure A2 summarizes the results of the model calibration process, based on prior work.[2] With the generic model inputs from Table A1 fixed, we compared our model behavior against i) data on children with non-polio acute flaccid paralysis who reported no receipt of OPV for northern India (modeled separately for Western Uttar Pradesh (WUP) and Bihar) and northwest (NW) Nigeria; ii) data on polio incidence and die-out of endemic WPV transmission for all situations and serotypes (shown in Figure A2 for WPV1 and WPV3 in northern India and northwest Nigeria and for all 3 WPV serotypes in the USA); iii) data from WPV importation outbreak behavior in the Netherlands, Tajikistan, and Albania; iv) data on age distributions of cases for all situations in which meaningful data was available (shown in Figure A2 for the Netherlands, Tajikistan, and Albania); v) available serogical data on the effect of secondary OPV immunity in the USA and Cuba (not shown); vi) indigenous emergence of cVDPVs (shown in Figure A2 for northern India, NW Nigeria (both serotype 2), Haiti, and Madura in Indonesia (both serotype 1); and vii) no indigenous emergence of cVDPVs in all other situations and serotypes (die-out of serotype 1 OPV-related viruses shows in Figure A2 for Cuba and Haiti). We subsequently applied the model to successfully reproduce the asymptomatic transmission of an imported WPV1 in Israel in 2013.[31]" [45, online supplement pp. 1-2]

Most critically in the context of certification questions, the DEB model approximates interruption of live poliovirus transmission (i.e., of an OPV, WPV, vaccine-derived poliovirus (VDPV), or OPV-related strain) in a population to occur when the effective infectiousness-weighted proportion of the population infectious with that poliovirus drops below 5 per million people (i.e., the transmission threshold EPI^{*}).[2] While this simplifies the true die-out behavior, which depends on local heterogeneity and chance, it appears capable of generating WPV die-out times consistent with observations in a broad range of settings.[2 30 31 41] Moreover, when applied to the persistence of OPV-related viruses that evolve to fully transmissible and neurovirulent circulating VDPVs (cVDPVs), the approximation produces cVDPV outbreaks for conditions in which they occurred (e.g., in Hispaniola[46] and Nigeria[47]) and no cVDPV outbreaks for conditions in which they did not occur despite OPV use and cessation (e.g., in Cuba[48] and the USA[49]).[2]

Use of the hypothetical model clarified that under-vaccinated subpopulations can sustain poliovirus transmission independently despite high coverage in the surrounding general population and showed how the minimum coverage needed to interrupt transmission depends on the degree of isolation and the relative size of the under-vaccinated subpopulation.[38] To explore the role of hard-to-reach under-vaccinated subpopulations for certification questions, we modified the hypothetical model in two ways and added a stochastic layer on top of the DEB model to simulate polio case detections. The first modification consisted of desynchronizing the time when vaccination starts in the general and under-vaccinated subpopulations to simulate the concept of a population that remains inaccessible for an extended period of time. Specifically, we run the model, which assumes equal birth and death rates and thus no population growth (Table 1), without vaccination for 30 years to settle into the endemic equilibrium, and then instantly change the routine immunization coverage in the general population with three OPV doses to 0.95, which lies well above the threshold of 0.92 needed to interrupt transmission in a closed population with similar characteristics.[38] However, we assume that the under-vaccinated subpopulation initially remains completely unreached by vaccination, with vaccine introduction

For peer review only - http://bmjopen.bmj.com/site/about/guidelines.xhtml

in the under-vaccinated subpopulation occurring 10 years after vaccine introduction in the general population. Desynchronizing the introduction of vaccination affects the dynamics and effectively makes it more difficult to interrupt transmission after introducing vaccination in the last subpopulation. To offset this effect, we consider a different hypothetical population with a slightly higher average per-dose take rate for OPV of 0.6 instead of 0.5 in the original analysis[38] (e.g., due to lower exposure to enteric viruses that interfere with vaccine take [50]). As in the original analysis,[38] we vary the coverage in the under-vaccinated subpopulation, the relative size of the under-vaccinated subpopulation compared to the total population, and the degree of preferential mixing, characterized by the proportion of potentially infectious contacts of individuals in the under-vaccinated subpopulations with other individuals in the same subpopulation (p_{within}).

Figure A3 shows the behavior of the incidence of infections in fully susceptible individuals and infants born with maternal immunity as a function of the varied DEB model inputs. Generally, the model yields incidence proportional to population size before vaccination starts. After the introduction of vaccination with high coverage in the general population, the initially still unvaccinated subpopulation becomes the main contributor to the total incidence. However, with less than 100% coverage in the general population and some interaction between the two populations (i.e., $p_{within}<1$), some incidence continues to occur in the general population as exported viruses find unvaccinated individuals. Lower values of p_{within} imply more interaction between the two populations and result in more incidence in the general population before vaccination in the under-vaccinated subpopulation begins (middle column of Figure A3). The relative size of the under-vaccinated subpopulation also affects the extent to which the under-vaccinated subpopulation affects the general population (right column of Figure A3). With base case model inputs, the minimum coverage in the under-vaccinated subpopulation to interrupt transmission equals 0.82. Higher coverage values mean interruption occurs sooner after the introduction of vaccination in the under-vaccinated subpopulation, while lower coverage values mean that transmission continues and can eventually rebound and settle into a new equilibrium (left column in Figure A1).

While the prior approach used fully stochastic transmission models to randomly generate infections, die-out, and polio cases and detections,[19-22] for efficiency we use post-hoc processing of DEB model results to randomly generate only the times when polio cases and detections stochastically occur. Specifically, for each setting of the DEB model, we record the deterministic realization of the daily incidence of infections in fully susceptible individuals of any age and 50% of infants less than 3 months of age born with maternal immunity, which represent the only individuals at risk of becoming a polio case in the DEB model.[2] We then randomly determine the number of polio cases resulting from the infection incidence on each day using a Poisson draw with a rate equal to the infection incidence multiplied by the PIR. For each generated case, we use a separate uniform random draw to determine whether it results in a detection based on each of the detection probabilities in Table 1 (e.g., a random uniform draw of 0.45 would mean that the case results in a detection only for detection probabilities of more than 0.45). For each DEB model setting, we repeat the post-hoc stochastic process 10,000 times and we start generating cases 10 years before vaccination starts in the general population, which we assume starts vaccination 10 years earlier than the under-vaccinated subpopulation (see appendix). The precise choice of when to start randomly generating cases exerts negligible

For peer review only - http://bmjopen.bmj.com/site/about/guidelines.xhtml

influence on the results as long as it occurs before cases become rare (i.e., before the interval between cases becomes longer). For simplicity, although prior work showed the significant role of serotype differences and seasonality,[20, 22] the hypothetical model inputs reflect WPV1 and assumes no seasonality. A limitation arises from the direct scaling of the DEB model with absolute population size, such that die-out depends on the effective proportion of infectious individuals rather than the absolute number. Using the post-hoc stochastic analysis, the absolute population size affects the number of infections, which affects the typical interval between detected cases. We show that CNC95% increases substantially for smaller absolute population sizes.

Our initial findings motivated analysis of the minimum population size that can sustain WPV circulation on its own to determine whether the upper bound on the CNC95% of 9 years could occur in real populations. However, for population sizes far below 100,000, the DEB model becomes inadequate because it allows prevalence to remain above the die-out threshold even with only fractional numbers of infections (i.e., less than one infected person). Therefore, we used a fully stochastic model to explore questions of minimum population size. We run the model 10,000 times for different population sizes and initial conditions and report the distribution of the duration of circulation and the CNC95%.

Exploration of the causal interactions relevant to global WPV certification decisions with an influence diagram (Figure 5)

Table A2 provides indicative estimates of the key quantities in Figure 5, based on the literature. Figure 5 assumes that policy makers explicitly or implicitly set a *desired confidence about no WPV circulation at OPV cessation*. In reality, they may focus on the confidence at certification, but given that it takes some fixed *preparation time needed between certification and OPV cessation*, any set confidence at the time of certification corresponds to some *desired confidence about no WPV circulation at OPV cessation.* A higher desired confidence level implies a longer *time between last detection and certification.* This time decreases with increasing investments in immunization and surveillance from the *GPEI budget* through *population immunity maintenance* and the *perceived pre-certification surveillance quality*, respectively. The main drawback of a longer *time between last detection and OPV cessation* comes in the form of longer OPV use in most countries, which results in *planned immunization costs* and *OPV-related pre-cessation polio cases* (i.e., vaccine-associated paralytic polio and VDPVs). In addition, with some globally-recommended or nationally-preferred *duration of IPV after OPV cessation,* later OPV cessation would imply greater overall IPV costs, because global IPV use already started (i.e., only the end, and not the beginning of IPV use depends on the timing of cessation of the last OPV serotypes). These drawbacks together lead to *financial and societal costs of planned immunization*. This includes the monetary equivalent of the *OPV-related polio cases*, which depends on the country income-level-dependent *societal costs per polio case.*

On the left side of Figure 5, we see the benefits of setting a higher *desired confidence about no WPV circulation at OPV cessation.* A higher confidence implies a lower *probability of a WPV reemergence after OPV cessation* (all else being equal). However, this probability does not directly equal the reciprocal of the confidence in the event of a *gap between perceived and actual surveillance quality.* Specifically, if the *perceived pre-certification surveillance quality* exceeds

For peer review only - http://bmjopen.bmj.com/site/about/guidelines.xhtml

the *actual pre-certification surveillance quality*, then the true *probability of WPV reemergence after OPV cessation* equals more than 1 minus the *desired confidence about no WPV circulation at OPV cessation*, and vice versa. This potential discrepancy highlights the importance of continued assessment of surveillance quality and assurance of high surveillance quality. A lower *GPEI budget* also decreases *population immunity maintenance* and thus increases the *probability of WPV reemergence after OPV cessation*, which implies an increase in *expected WPV reemergence outbreaks.* Unlike other possible types of post-cessation outbreaks, a WPV reemergence would almost certainly occur in the most challenging populations. Any such reemergences would lead to *expected polio cases due to WPV reemergence outbreaks* and *expected costs to respond to WPV emergence outbreaks*. The expected costs and cases decrease with higher *post-cessation surveillance quality*, which affects the extent of viral spread at the time of outbreak detection (and beyond), and with a better *outbreak response quality and timeliness,* which both increase the probability of effective outbreak control.[51] However, the occurrence of any outbreaks comes with some probability of uncontrolled outbreaks, either by failing to control the original outbreak virus, or by creating new cVDPV outbreaks with the OPV vaccine used in the response. This implies some *probability of OPV restart due to WPV reemergences*, which would carry very significant *expected costs due to an OPV restart triggered by WPV reemergence* and *expected cases due to an OPV restart triggered by WPV emergence* (Table A2). For moderate or high *probability of OPV restart due to WPV reemergences*, the resulting *expected costs due to OPV restarts triggered by WPV reemergence* and *expected cases due to OPV restarts triggered by WPV reemergence* would likely dwarf the costs and cases associated with any controlled outbreaks due to WPV reemergences and would therefore drive the *expected financial and societal costs of possible WPV reemergences.*

Together with the *surveillance costs*, which act to moderate the costs of delayed OPV cessation or premature OPV cessation, the *expected financial and societal costs of possible WPV reemergences* and the *financial and societal costs of planned immunization* together make up the *total financial and societal costs (associated with any given desired confidence about no WPV circulation at OPV cessation).* The costs of possible WPV emergences and the costs of planned immunization move in opposite directions as a function of the *desired confidence about no circulation at OPV cessation.*

Figure 5 also highlights the consequences of the GPEI already scaling down some of its supplemental immunization and surveillance activities. While scaling down saves costs in the short term, doing so could lead to larger long-term costs by delaying certification and OPV cessation (i.e., requiring higher confidence about no circulation), which would imply that OPV cessation could occur in the context of lower global population immunity to transmission and lower ability to rapidly detect outbreaks. This ultimately implies an increase in the expected *total financial and societal costs (associated with any given desired confidence about no WPV circulation at OPV cessation)*. For visual simplicity, Figure 5 omitted some additional complexity involved in this decision. Furthermore, given that the confidence about no circulation increases with time after the last detection, we could have equivalently centered Figure 5 around finding the optimal time between the last detection and certification or OPV cessation. The amounts in Table A2 highlight the significant financial and humanitarian stakes involved in finding the optimal *desired confidence about no WPV circulation at OPV cessation.*

For peer review only - http://bmjopen.bmj.com/site/about/guidelines.xhtml

Table A1: Generic inputs of the DEB model[2 30] (adopted from the online supplement of Duinter Tebbens et al., 2017[45])

Model input (symbol)	Best estimate	Source
Relative susceptibility (σ) of recent immunity states (for PV1;PV2;PV3)		[52 53]
- Maternally immune	0.78;0.79;0.77	
- 1 successful IPV	0.91;0.92;0.90	
- 2 successful IPV	0.80;0.80;0.79	
- ≥ 3 successful IPV	0.72;0.72;0.71	
- 1 LPV infection	0.42;0.43;0.41	
- ≥ 2 LPV infections	0.21;0.22;0.20	
- IPV and LPV	0.21;0.22;0.20	
Duration of latent period (ξ^{fec} or ξ^{oro}, in days)	~ 3[a]	[52 53]
Duration of fecal infectiousness (γ^{fec}, in days) of recent immunity states (for PV1;PV2;PV3)		[52 53]
- Fully susceptible	28.0;27.8;28.3	
- Maternally immune	24.6;24.6;24.6	
- 1 successful IPV,	24.5;24.4;24.7	
- 2 successful IPV	21.1;20.8;21.3	
- ≥ 3 successful IPV	18.0;17.7;18.2	
- 1 LPV infection	11.6;10.5;10.5	
- ≥ 2 LPV infections	10.1;8.9;8.9	
- IPV and LPV	10.1;8.9;8.9	
Duration of oropharyngeal infectiousness (γ^{oro}, in days) of recent immunity states (no serotype differences)		[52 53]
- Fully susceptible	13.4	
- Maternally immune	11.9	
- 1 successful IPV	9.9	
- 2 successful IPV	6.6	
- ≥ 3 successful IPV	6.1	
- 1 LPV infection	5.0	
- ≥ 2 LPV infections	3.7	
- IPV and LPV	3.7	
Relative fecal infectiousness (π^{fec}) of recent immunity states (for PV1;PV2;PV3)		[52 53]
- Maternally immune	0.96;0.96;0.95	
- 1 successful IPV	0.92;0.92;0.91	
- 2 successful IPV	0.70;0.69;0.68	
- ≥ 3 successful IPV	0.61;0.59;0.59	
- 1 LPV infection	0.39;0.43;0.43	
- ≥ 2 LPV infections	0.20;0.23;0.23	
- IPV and LPV	0.20;0.23;0.23	
Relative oropharyngeal infectiousness (π^{oro}) of recent immunity states (no serotype differences)		[52 53]
- Maternally immune	0.68	
- 1 successful IPV	0.30	
- 2 successful IPV	0.17	
- ≥ 3 successful IPV	0.12	
- 1 LPV infection	0.33	
- ≥ 2 LPV infections	0.21	
- IPV and LPV	0.21	
Number of infection stages		
- Latent period (r)	2	
- Infectious period (s)	4	
Relative weight of infection stages, compared to average weight over the infectious period (θ_j, j=0,…,r+s-1)		[52 53]

For peer review only - http://bmjopen.bmj.com/site/about/guidelines.xhtml

- Infection stage 0 and 1 (latent stages)<br>- Infectious stage 2<br>- Infectious stage 3<br>- Infectious stage 4<br>- Infectious stage 5	0<br>12/17<br>40/17<br>12/17<br>4/17	
IPV immunity delay (φ, in days)	7	54
Number of waning stages (*nw*)	5	
Shape of waning function (z_w)	5	52 53
Average time to reach last waning stage (ρ, in days)<br>- Type 1&2<br>- Type 3	 4×365<br>3×365	52 53
Average time for maternal immunes to wane to fully susceptible (ρ_{MI}, in days)	0.25×365	52 53
Relative susceptibility (σ) for last waning stage (no serotype differences)<br>- 1 successful IPV<br>- 2 successful IPV<br>- ≥ 3 successful IPV<br>- 1 LPV infection<br>- ≥ 2 LPV infections<br>- IPV and LPV	 1.0<br>1.0<br>1.0<br>0.8<br>0.7<br>0.7	52 53
Duration of fecal infectiousness (γ^{fec}, in days) of last waning stage (for PV1;PV2;PV3)<br>- 1 successful IPV<br>- 2 successful IPV<br>- ≥ 3 successful IPV<br>- 1 LPV infection<br>- ≥ 2 LPV infections<br>- IPV and LPV	 26.6;26.4;26.9<br>25.2;25.0;25.5<br>23.8;23.6;24.1<br>14.0;13.9;14.1<br>11.4;11.4;11.6<br>11.4;11.4;11.6	52 53
Duration of oropharyngeal infectiousness (γ^{oro}, in days) of last waning stage (no serotype differences)<br>- 1 successful IPV<br>- 2 successful IPV<br>- ≥ 3 successful IPV<br>- 1 LPV infection<br>- ≥ 2 LPV infections<br>- IPV and LPV	 11.4<br>6.7<br>6.6<br>6.7<br>4.0<br>4.0	52 53
Relative fecal infectiousness (π^{fec}) of last waning stage (no serotype differences)<br>- 1 successful IPV<br>- 2 successful IPV<br>- ≥ 3 successful IPV<br>- 1 LPV infection<br>- ≥ 2 LPV infections<br>- IPV and LPV	 0.95<br>0.9<br>0.85<br>0.5<br>0.3<br>0.3	52 53
Relative oropharyngeal infectiousness (π^{oro}) of last waning stage (no serotype differences)<br>- 1 successful IPV<br>- 2 successful IPV<br>- ≥ 3 successful IPV<br>- 1 LPV infection<br>- ≥ 2 LPV infections<br>- IPV and LPV	 0.43<br>0.25<br>0.13<br>0.5<br>0.3<br>0.3	52 53
Number of reversion stages (*h*)	20	
Shape of reversion function with respect to:<br>- R_0 (z_r)<br>- ln(PIR) (z_p)	 1<br>2.5	

For peer review only - http://bmjopen.bmj.com/site/about/guidelines.xhtml

Average time to reach last reversion stage (ε, in days) (for PV1;PV2;PV3)	620.5; 408; 620.5	[30]
Paralysis-to-infection ratio for fully susceptible individuals infected with OPV (PIR_0) (for PV1; PV2;PV3)	0.26×10^{-6}; 1.2×10^{-6}; 1.8×10^{-6}	
Paralysis-to-infection ratio for fully susceptible individuals infected with FRPV (PIR_{h-1}) (for PV1; PV2;PV3)	0.005; 0.0005; 0.001	[2 14 54]
Relative R_0 of OPV vs. FRPV (τ_0) (for PV1; PV2; PV3)	0.37;0.55;0.25	[2 52 53]
Effective infectious proportion below which we assume 0 force-of-infection (transmission threshold *EPI**)	5/1,000,000	
Relative PIR for maternally immunes compared to fully susceptible individuals ($RPIR_{MI}$)	0.5	
Ratio of R_0 by serotype in the same setting (PV1:PV2:PV3)	1:0.9:0.75	[30]
Average incubation period (δ, in days)	10	[54 55]
Demographics for all situations	Time series 1950-2100	[56]

Acronyms: CDC = (U.S.) Centers for Disease Control and prevention; cVDPV = circulating vaccine-derived poliovirus; DEB = differential equation-based FRPV = fully-reverted poliovirus; GPLN = Global Polio Laboratory Network; IPV = inactivated poliovirus vaccine; LPV = live poliovirus; OPV = oral poliovirus vaccine; PIR = paralysis-to-infection ratio; PV(1,2,3) = poliovirus (type 1, 2, or 3, respectively); R_0 = basic reproductive number; UN = United Nations; USA = United States of America; VAPP = vaccine-associated paralytic poliomyelitis; VP1 = viral protein 1; WPV(1,2,3) = wild poliovirus (type 1, 2, or 3, respectively)

Notes: [a] Mean estimates obtained from experts and used in the model for the different immunity states, serotypes, and excretion modes vary between 2.85 and 3.37 days

For peer review only

Table A2: Indicative estimates of key variables from Figure 5

Variable	Estimate	Notes and sources
Preparation time needed between certification and OPV cessation	Approximately 1 year	Depends on when setting of the OPV cessation date occurs relative to certification [57]
Planned immunization costs	$1 billion in external GPEI funds per year, plus internal contributions	Most of the $1.1 billion GPEI budget for 2016 was for immunization and coordination of activities;[58] Countries may internally contribute at a similar rate as the external contributions;[59] The current GPEI budget projects a decrease from 2018 forward, which would imply some offset of costs for maintenance of activities, or alternatively the activities previously supported by external contributions may end, which would imply declines in programmatic activities and quality
OPV-related polio cases	Hundreds per year	Vaccine-associated paralytic polio cases,[60] which depends on timing of IPV doses,[61] and presumably local cVDPV outbreaks[62]
Surveillance costs	Around $100 million per year	The 2016 GPEI budget included $67 million in external support for surveillance and laboratories,[58] with additional significant internal contributions by countries [59 63]
Probability of OPV restart due to WPV reemergence	Unknown	Prior studies estimated an approximately 5% chance of an OPV restart due primarily to OPV-associated risks, although the actual implementation of risk management policies was not as good as suggested by these models.[59 64]
Immunization costs associated with an OPV restart	$ billions (hundreds of millions per year)	An OPV restart would involve reintroduction of OPV vaccination in most countries in perpetuity, with supplemental immunization activities needed in countries with insufficient routine immunization coverage.[59] Significant uncertainty exists about what an OPV restart would look like in practice.
Expected cases due to an OPV restart	Up to thousands per year	Reintroduction of OPV in most countries would result in hundreds of vaccine-associated paralytic polio cases per year and could result in continued cVDPV outbreaks in countries with insufficient routine immunization coverage that do no conduct regular preventive supplemental immunization activities.[59 64]

For peer review only - http://bmjopen.bmj.com/site/about/guidelines.xhtml

Figure A1: Schematic of the DEB model structure, adopted from Duintjer Tebbens et al. (2013)[2, p. 706]

(a) Immunity states and flows between them due to epidemiological events

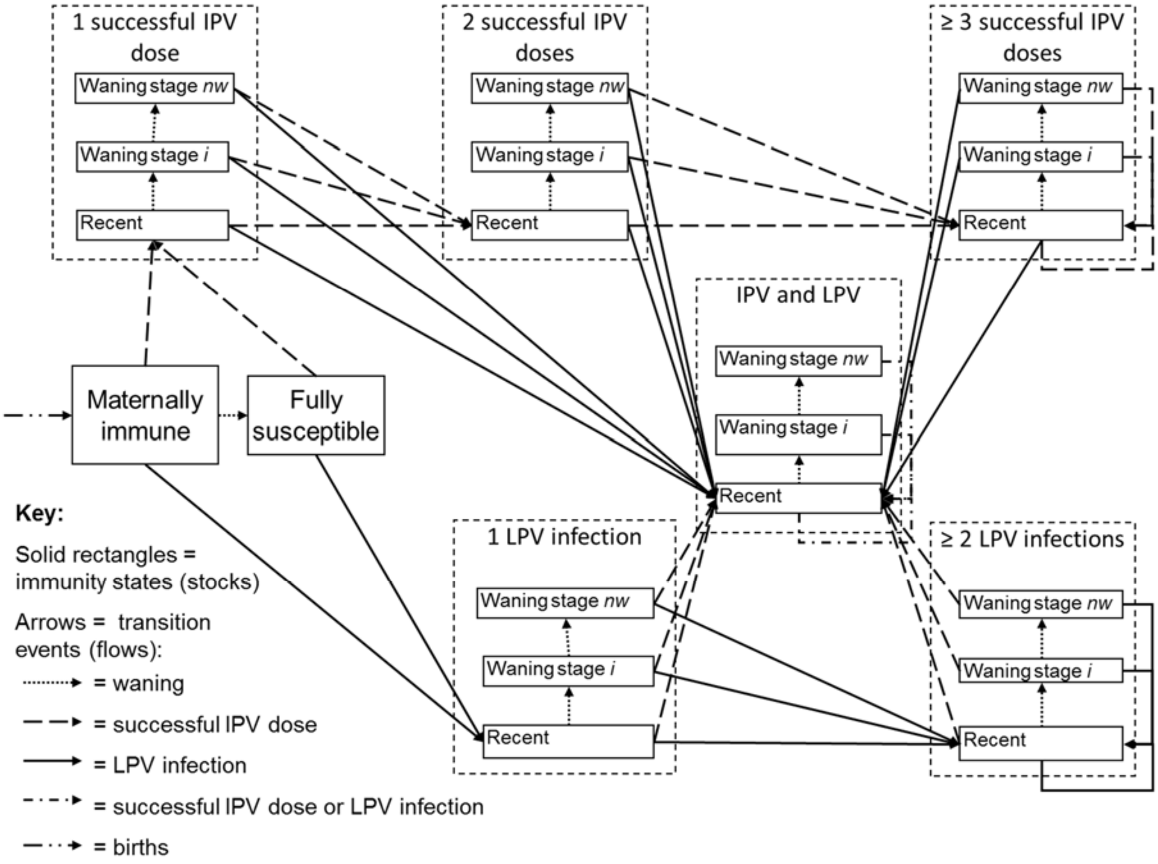

For peer review only - http://bmjopen.bmj.com/site/about/guidelines.xhtml

(b) Progression through infection and reversion stages

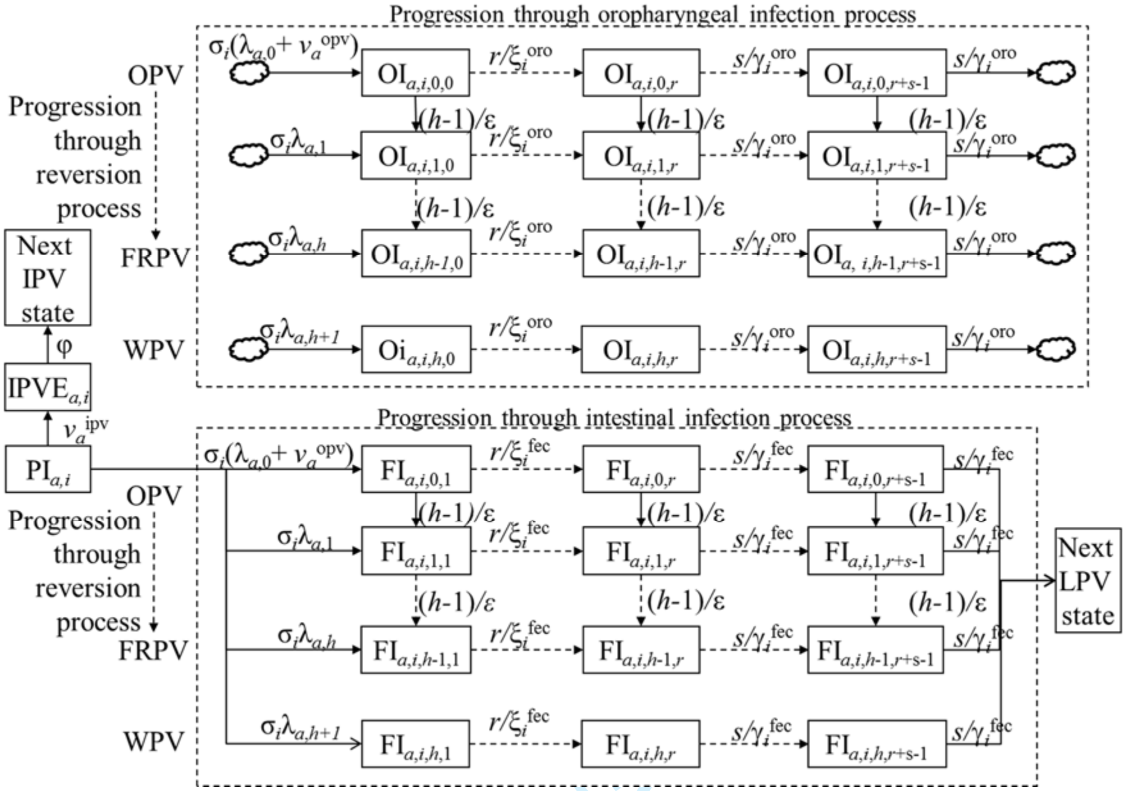

“Acronyms: FRPV = fully-reverted poliovirus; IPV = inactivated poliovirus vaccine; OPV = oral poliovirus vaccine; WPV = wild poliovirus**; Symbols:** $PI_{a,i}$ = partially infectible in age group *a* and immunity state *I*; $IPVE_{a,i}$ = IPV-exposed individual from immunity state *i* and age group *a*; $FI_{a,i,j,k}$ ($OI_{a,i,j,k}$) = individual in age group a from immunity state *i*, infected with virus strain *j* and in fecal (oropharyngeal) infection stage *k*; $\lambda_{a,j}$ = force-of–infection to age group *a* for virus strain *j;* ν_a^{ipv} (ν_a^{opv}) = force-of-IPV(OPV)-vaccination to age group *a* as a result of routine and supplementary immunization; σ_i = relative susceptibility for immunity state *i*; ξ_i^{fec} (ξ_i^{oro}) = average duration of the fecal (oropharyngeal) latent period for immunity state *i*; γ_i^{fec} ($\gamma_i^{oro)}$ = average duration of the fecal (oropharyngeal) infectious period for immunity state *i*; *φ* = IPV immunity delay; *h* = number of reversion stages; *r* = number of latent stages; *s* = number of infectious stages” [2, p. 706]

For peer review only - http://bmjopen.bmj.com/site/about/guidelines.xhtml

Figure A2: Summary results from the model calibration process, adapted from Duintjer Tebbens et al. (2013)[2]

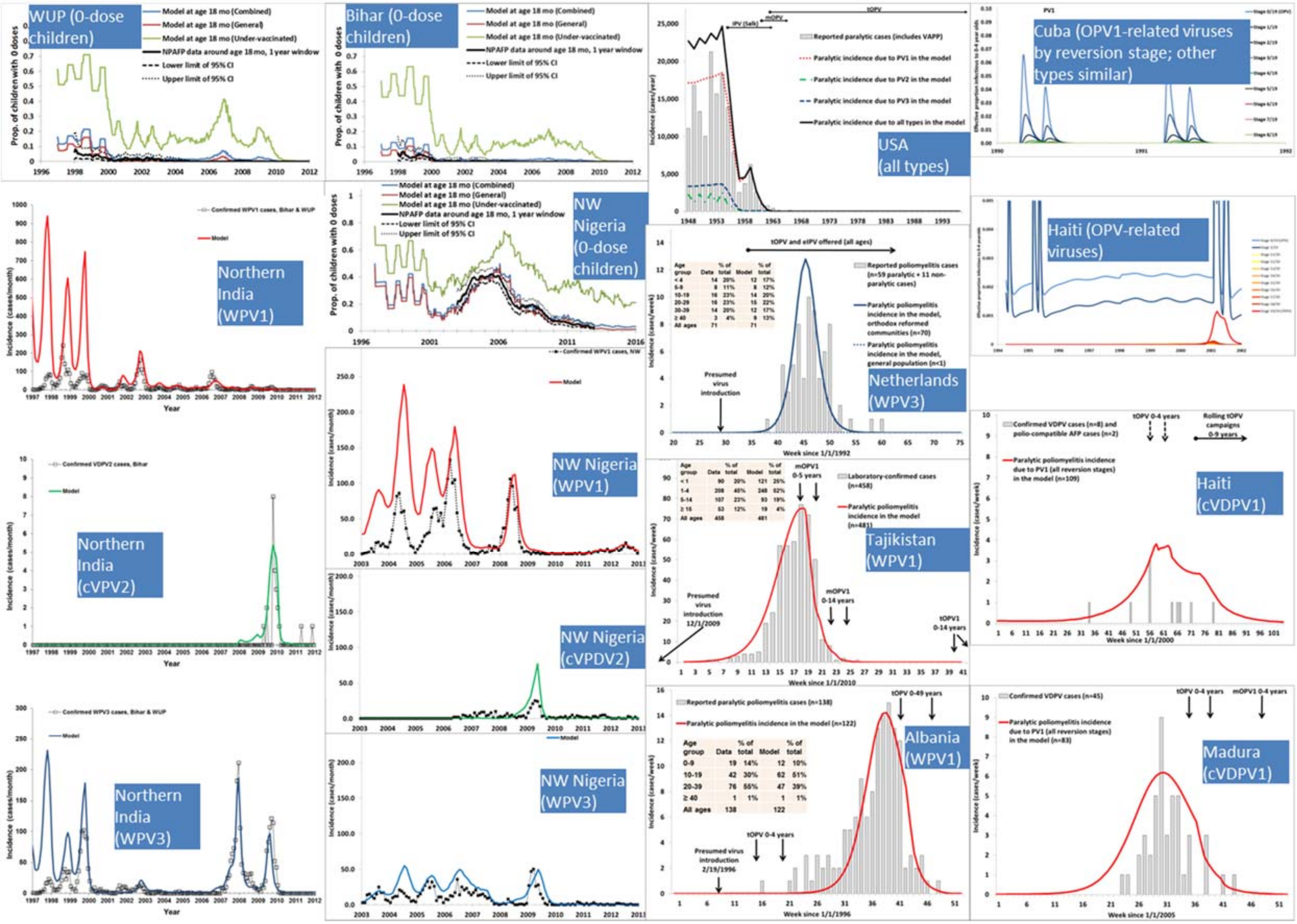

12

Figure A3: Differential-equation based model results for base case model inputs and varied coverage (left column), varied degree of isolation with coverage 0.82 (middle column), and varied relative size with coverage of 0.82 (right column). The y-axis scales linearly with total population size (all figures assume a total population size of 1 million).

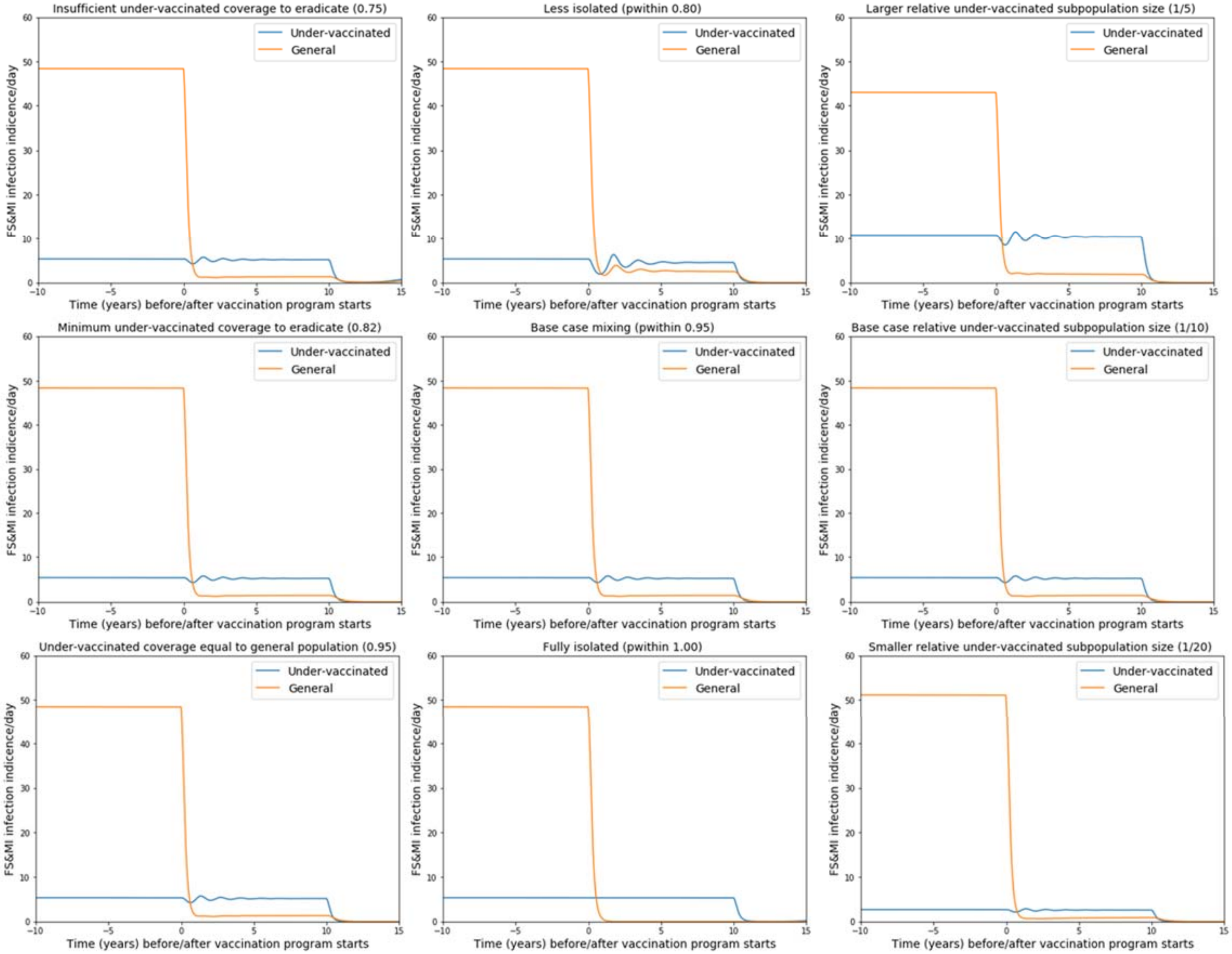

13
